# Design, Synthesis, and Biological Evaluation of *N*,*N*-Diphenylaniline-Based Derivatives as Antiproliferative Agents and ABL TK Inhibitors Against CML

**DOI:** 10.3390/ph19030416

**Published:** 2026-03-04

**Authors:** Belgin Sever, Halilibrahim Ciftci

**Affiliations:** 1 Department of Pharmaceutical Chemistry, Faculty of Pharmacy, Anadolu University, Eskisehir 26470, Türkiye; belginsever@anadolu.edu.tr; 2 Department of Molecular Biology and Genetics, Burdur Mehmet Akif Ersoy University, Istiklal Campus, Burdur 15200, Türkiye

**Keywords:** *N*,*N*-diphenylaniline, chronic myeloid leukemia, Abl TK, cytotoxicity, apoptosis, molecular docking

## Abstract

**Background/Objectives**: Targeting ABL tyrosine kinase (TK) remains a cornerstone of chronic myeloid leukemia (CML) therapy. **Methods:** In this study, a series of novel 4-((2-(4-(aryl)thiazol-2-yl)hydrazineylidene)methyl)-*N*,*N*-diphenylaniline derivatives (**1–12**) were synthesized through the reaction of 2-(4-(diphenylamino)benzylidene)hydrazine-1-carbothioamide (intermediate **A**) with substituted 2-bromo-1-arylethanones. Cytotoxic activity was evaluated in K562 CML cells using the MTT assay. The most active compound was further assessed in HL-60 acute myeloid leukemia (AML) cells and healthy peripheral blood mononuclear cells (PBMCs). Apoptosis induction was analyzed by Annexin V/ethidium homodimer staining, while ABL TK inhibition was determined using the ADP-Glo kinase assay. Molecular docking studies were performed to investigate binding interactions within the ATP-binding site of ABL TK, and pharmacokinetic properties were also predicted. **Results:** Intermediate **A** demonstrated superior antiproliferative activity compared to derivatives **1–12** and exhibited cytotoxicity comparable to imatinib in K562 cells (IC_50_ = 6.15 ± 1.26 µM vs. 5.14 ± 1.44 µM, respectively). In HL-60 cells, intermediate **A** showed an IC_50_ of 12.04 ± 1.70 µM, similar to imatinib. Notably, intermediate **A** displayed enhanced selectivity toward K562 cells over PBMCs (SI = 12.9) relative to imatinib (SI = 6.2). The compound significantly induced apoptosis in K562 cells and inhibited ABL TK activity. Docking studies revealed a distinct binding orientation within the ATP-binding pocket of ABL TK. The compound showed acceptable predicted physicochemical and ADME characteristics based on in silico analysis. **Conclusions:** Intermediate **A** emerges as a significant anti-CML candidate exhibiting potent cytotoxic, apoptotic, and moderate ABL TK inhibitory activity, together with a favorable selectivity profile.

## 1. Introduction

Leukemia is the most common cancer in children, accounting for 28% of all cases, followed closely by central nervous system (CNS) tumors at 27%, approximately one-third of which are benign or borderline malignant. In adolescents, the distribution differs: CNS tumors are the most prevalent (22%), with more than half classified as benign or borderline malignant, followed by lymphoma (19%) and leukemia (13%) [1].

Leukemia is broadly classified into two major types: lymphoid and myeloid. Lymphoid leukemias include acute lymphoblastic leukemia (ALL) and chronic lymphocytic leukemia (CLL), whereas myeloid leukemias comprise acute myeloid leukemia (AML) and chronic myeloid leukemia (CML). ALL and CLL arise from lymphoid precursor cells that differentiate into immune cells such as T and B lymphocytes. On the other hand, AML and CML originate from myeloid progenitor cells responsible for producing red blood cells, platelets, and various types of white blood cells [2,3]. CML accounts for approximately 15–20% of all leukemia cases, with an incidence of 1–2 cases per 100.000 individuals annually [4,5,6].

A hallmark event in CML pathogenesis is the fusion of the ABL1 gene on chromosome 9 with the BCR gene on chromosome 22, resulting in the formation of the BCR::ABL1 oncoprotein. This constitutively active tyrosine kinase (TK) drives uncontrolled cellular proliferation and survival through activation of multiple signaling pathways, including Ras/mitogen-activated protein kinase (MAPK), phosphoinositide 3-kinase (PI3K), and signal transducer and activator of transcription 5 (STAT5) [7,8,9,10,11,12,13].

**Figure 1:**
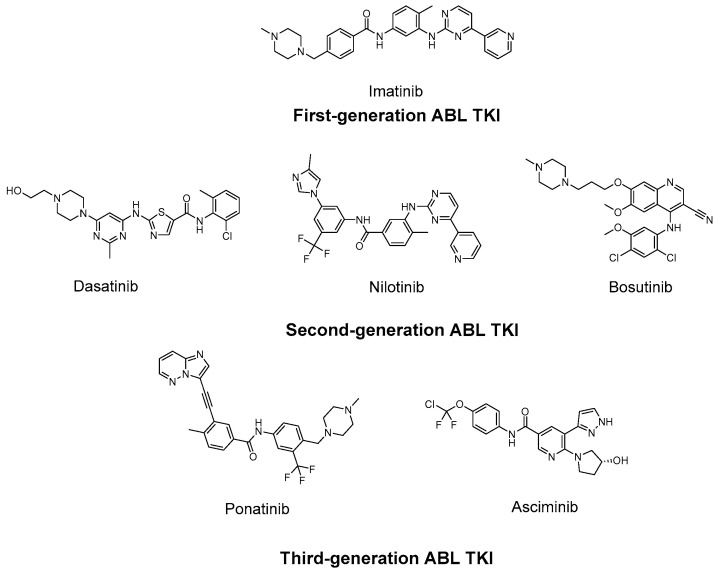
The chemical structures of first-, second- and third-generation ABL TKIs.

The introduction of BCR-ABL tyrosine kinase inhibitors (TKIs) has dramatically improved long-term outcomes for patients with CML. Imatinib (Figure 1), the first TKI approved for CML, significantly enhanced survival and revolutionized disease management. Subsequently, second-generation TKIs, dasatinib (Figure 1), nilotinib (Figure 1), and bosutinib (Figure 1), and third-generation TKIs, ponatinib (Figure 1) and asciminib (Figure 1), have further increased response rates and broadened therapeutic options, enabling individualized treatment strategies [14,15,16,17].

Dasatinib is approximately 350 times more potent than imatinib in vitro. Nilotinib, a structurally modified derivative of imatinib with high affinity for the ATP-binding site, exhibits 30–50 times greater potency. Bosutinib acts as a dual Src/ABL TK inhibitor. More recently, asciminib received FDA approval for CML treatment. Unlike other TKIs, asciminib functions as a selective allosteric ABL TK inhibitor by targeting the myristoyl-binding pocket, thereby acting independently of ATP competition. According to the European LeukemiaNet (ELN) guidelines, imatinib, dasatinib, nilotinib, and bosutinib are recommended as preferred first-line therapies for newly diagnosed CML patients. Selection of the most appropriate first-line TKI requires careful assessment of patient comorbidities and the specific toxicity profile of each agent [18,19,20].

Despite the transformative impact of TKIs, several clinical challenges remain. These agents are associated with an increased risk of cardiovascular complications, which may contribute to or exacerbate renal dysfunction, particularly in patients with common risk factors such as hypertension, diabetes, and obesity. Additionally, resistance to TKIs may develop, and disease relapse can occur following treatment discontinuation. Relapse and disease progression are largely attributed to the persistence of leukemic stem cells, which exhibit dysregulation of critical biological processes, including autophagy, metabolic pathways, and epigenetic regulation [21,22,23,24,25].

Beyond achieving optimal therapeutic response, two increasingly important goals in CML management are minimizing TKI-related adverse effects and ensuring equitable access to effective and affordable treatments. Together, these factors define the overall clinical value of TKI therapy [26]. Consequently, the development of novel therapeutic agents remains a dynamic and evolving research field, aiming to overcome the limitations of current TKIs, reduce toxicity, and ultimately improve patient quality of life [27].

**Figure 2:**
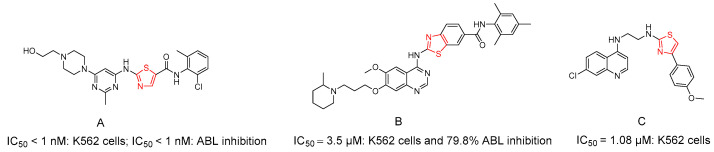
The chemical structures of thiazole-based ABL TKIs.

Several studies have demonstrated that the sulfur atom within the thiazole ring enhances a compound’s lipophilicity. Owing to its notable anticancer properties, thiazole has long served as a valuable scaffold in drug design, and numerous thiazole derivatives have shown inhibitory activity against ABL TK [28,29,30,31,32]. Dasatinib (**A**) (Figure 2) is a well-established thiazole-based ABL TKI (IC_50_ < 1 nM: K562 cells; IC_50_ < 1 nM: ABL inhibition) [30]. Furthermore, structural diversification of thiazole-containing systems has repeatedly yielded compounds with significant anti-CML activity. For instance, benzothiazole-quinazoline derivatives of dasatinib reported by Cai et al. [33] demonstrated notable activity, with compound **3**f (**B**) (Figure 2), exhibiting an IC_50_ of 3.5 μM in K562 cells and 79.8% ABL inhibition. Quinoline-thiazole hybrids have also displayed significant anti-CML activity, with a 4-methoxy-substituted analogue (compound **C**) [34] (Figure 2), achieving an IC_50_ of 1.08 μM and confirmed binding within the ABL TK active site.

**Figure 3:**
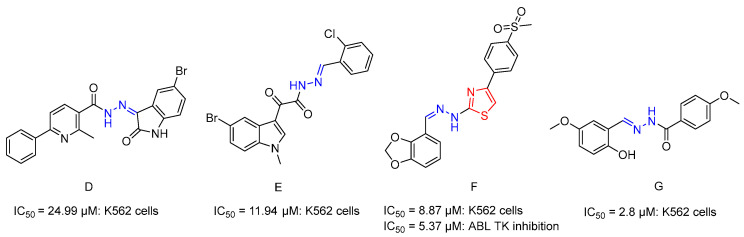
The chemical structures of hydrazone-based ABL TKIs.

Hydrazones exhibit greater stability in biological systems than structurally related imines, primarily due to resonance effects that reduce the reactivity of the adjacent carbon atom. They are particularly valued for their sensitivity to acidic environments, enabling targeted drug release within the acidic tumor microenvironment. Moreover, the presence of both an NH group and a carbonyl group allows hydrazones to function as hydrogen-bond donors and acceptors, thereby strengthening interactions with biological targets. Because of these advantageous properties, hydrazide-hydrazone and hydrazone linkers are widely employed in anticancer drug development. Over the past five decades, hydrazone derivatives incorporating diverse heterocyclic frameworks have emerged as significant chemotherapeutic agents. As potent inhibitors of cancer-related enzymes, they represent a versatile chemical class capable of modulating multiple oncogenic pathways, offering significant potential for the development of next-generation anticancer therapies [35,36,37]. Several hydrazide-hydrazone analogues [38,39,40,41] (Figure 3) have demonstrated efficacy against CML cells. Abdel-Aziz et al. reported a series of 6-aryl-2-methylnicotinic acid hydrazones, in which the 2-chloro-substituted analogue (compound **D**) [38] (Figure 3) exhibited moderate cytotoxicity against K562 cells (IC_50_ = 24.99 μM). In another study, novel indole-aryl fused keto hydrazide-hydrazone analogues were evaluated against eight human cancer cell lines, including K562 cells. Among them, the 2-chloro-substituted compound **E** [39] demonstrated enhanced antiproliferative activity in K562 cells (IC_50_ = 11.94 μM). Furthermore, a thiazole–hydrazone hybrid bearing a methylsulfonyl substituent (compound **F**) [40] (Figure 3) exhibited both cytotoxic activity in K562 cells (IC_50_ = 8.87 μM) and notable ABL TK inhibition (IC_50_ = 5.37 μM), highlighting the dual functional potential of such hybrid systems. More recently, a methoxy-substituted salicylaldehyde benzoylhydrazone (compound **G**) [41] (Figure 3) demonstrated high selectivity (IC_50_ = 2.8 μM), further supporting the pharmacological relevance of hydrazone-based scaffolds in leukemia research.

In the present study, novel *N*,*N*-diphenylaniline-based thiazolyl-hydrazone derivatives (**1**–**12**) were designed and synthesized via the reaction of 2-(4-(diphenylamino)benzylidene)hydrazine-1-carbothioamide (intermediate **A**) with appropriately substituted 2-bromo-1-arylethanones. The synthesized compounds were evaluated for their cytotoxic activity against K562 CML cells using the MTT assay. The most potent compound was subsequently tested against HL-60 AML cells and healthy peripheral blood mononuclear cells (PBMCs). Apoptotic effects in K562 cells were assessed by Annexin V/ethidium homodimer staining, while ABL TKI activity was measured using the ADP-Glo kinase assay. Molecular docking studies were conducted using Maestro software to examine binding interactions within the ATP-binding site of Abl TK. In addition, pharmacokinetic parameters and drug-likeness properties were predicted using SwissADME.

## 2. Results

### 2.1. Chemistry

**Scheme 1:**
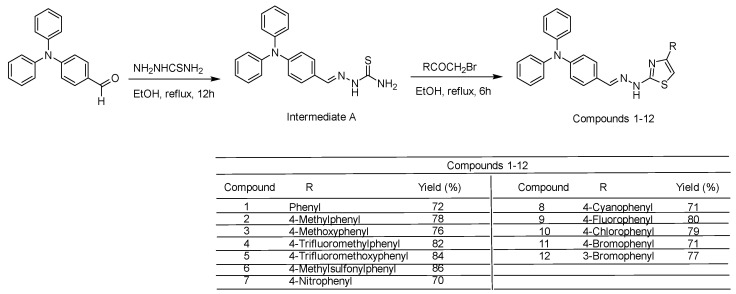
Synthetic route illustrating the preparation of intermediate **A** and its derivatives (**1**–**12**).

A series of novel *N*,*N*-diphenylaniline-derived thiazolyl-hydrazone compounds (**1**–**12**) were synthesized following the procedure illustrated in Scheme 1. The synthetic route commenced with the preparation of intermediate **A** [42,43], through the condensation of 4-(*N*,*N*-diphenylamino)benzaldehyde with thiosemicarbazide in refluxing ethanol. This intermediate subsequently underwent reaction with various substituted 2-bromo-1-arylethanones, leading to cyclization and formation of the targeted thiazole derivatives.

In the design of the target derivatives, para-substituted phenyl moieties were preferentially selected based on both literature precedents and our previous structure-activity relationship findings [34,40,41]. Para substitution was considered advantageous for maintaining molecular planarity and allowing clearer electronic modulation, while minimizing steric interference that may arise from ortho substitution. This orientation was anticipated to support favorable accommodation within the ATP-binding pocket and to facilitate interpretable structure-activity relationship trends. Notably, compound **12** incorporates a meta-substituted phenyl group in order to preliminarily explore positional effects within the same scaffold framework.

The chemical structures of all synthesized compounds were confirmed by ^1^H Nuclear Magnetic Resonance (NMR), ^13^C NMR, and high-resolution mass spectrometry (HRMS). The spectral data were fully consistent with the proposed thiazole frameworks, verifying the successful synthesis of the intended products.

### 2.2. Anticancer Evaluation and Mechanistic Investigation

The cytotoxic effects of intermediate **A** and compounds **1**–**12** were assessed in K562 CML cells using the MTT assay, with imatinib serving as the reference drug. All compounds were evaluated at a concentration of 10 µM.

**Figure 4:**
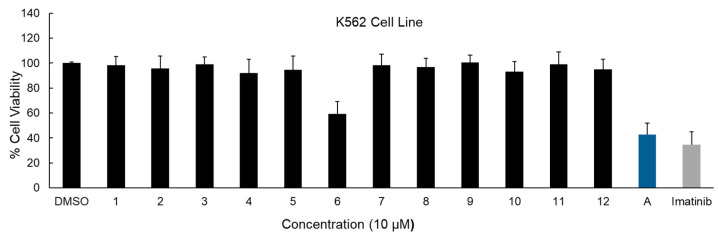
Cytotoxic activity of intermediate **A** and compounds **1**–**12** against the K562 cell line at a concentration of 10 µM, compared with imatinib.

Intermediate **A** demonstrated the highest reduction in K562 cell viability (approximately 57%), compared with imatinib, which achieved around 65% inhibition. Notably, the final synthesized compounds showed comparatively lower activity; among compounds **1**–**12**, only compound **6** exhibited the most pronounced cytotoxic effect, reducing cell viability by 40% (Figure 4).

**Figure 5:**
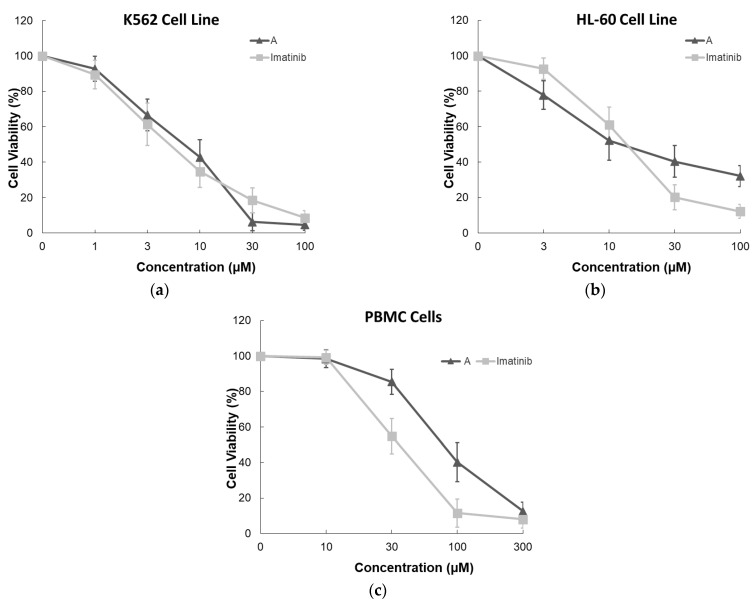
Cytotoxic activity of intermediate **A** in K562 (**a**), HL-60 (**b**) cells and PBMCs (**c**) at varying concentrations compared with imatinib.

Given its pronounced cytotoxic effect at 10 µM, intermediate **A** was further evaluated over a broader concentration range to characterize its dose–response behavior in K562 CML cells, using imatinib as the reference drug. Intermediate **A** demonstrated a clear dose-dependent reduction in cell viability. At lower concentrations (1–3 µM), both intermediate **A** and imatinib produced moderate and comparable decreases in viability. Notably, at 30 µM, intermediate **A** reduced cell viability to below 10%, approaching near-complete growth inhibition, with slightly greater potency than imatinib at the same concentration. At 100 µM, both compounds almost completely abolished cell viability, confirming strong antiproliferative activity in K562 cells (Figure 5a).

Consistent with these findings, intermediate **A** exhibited significant antiproliferative potency, with an IC_50_ value of 6.15 ± 1.26 µM, closely comparable to that of imatinib (IC_50_ = 5.14 ± 1.14 µM), indicating similar inhibitory effectiveness in BCR-ABL-positive leukemia cells (Table 1).

To investigate whether this activity extends beyond CML, the cytotoxic effects of intermediate **A** were further evaluated in HL-60 AML cells. Both intermediate **A** and imatinib displayed dose-dependent growth inhibition; however, a distinct response pattern emerged. At 3 and 10 µM, imatinib showed greater cytotoxic potency. At higher concentrations (30 and 100 µM), imatinib induced more pronounced inhibition (~80% and ~88%, respectively), whereas intermediate **A** produced moderate inhibition (~60% and ~68%, respectively) (Figure 5b). The IC_50_ value of intermediate **A** in HL-60 cells was 12.04 ± 1.70 µM, comparable to that of imatinib (12.55 ± 1.89 µM) (Table 1). These findings suggest that while intermediate **A** retains activity in AML cells, its potency appears more pronounced in the CML model.

The selectivity profile of intermediate **A** was subsequently assessed by comparing its cytotoxic effects in K562 cells and healthy PBMCs. At 10 µM, both compounds maintained high PBMC viability (~95–100%), indicating minimal toxicity toward normal cells. At 30 µM, imatinib caused a more substantial reduction in PBMC viability (~45%) compared to intermediate **A** (~15%). Although cytotoxicity increased at 100 and 300 µM for both compounds, intermediate **A** consistently preserved greater PBMC viability than imatinib (Figure 5c).

To quantitatively evaluate selectivity, the selectivity index (SI) was calculated as the ratio of IC_50_ in PBMCs to IC_50_ in K562 cells. Intermediate **A** exhibited an SI value of 12.9, whereas imatinib showed an SI of 6.2 (Table 1). These results indicate that intermediate **A** possesses a superior therapeutic window, demonstrating greater preferential cytotoxicity toward CML cells over healthy PBMCs compared to the reference drug.

**Figure 6:**
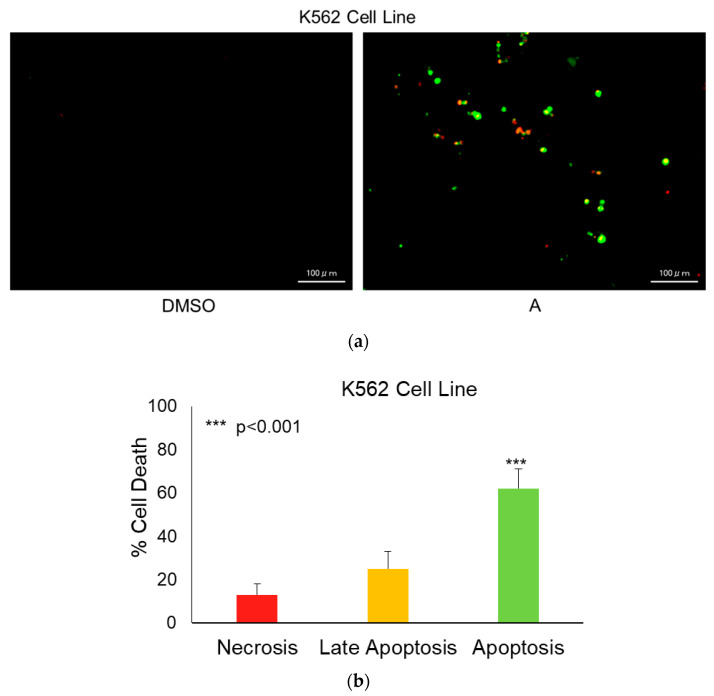
Induction of apoptosis in K562 cells following 12 h treatment with intermediate **A**. (**a**) Representative fluorescence images showing early apoptotic (green), late apoptotic (yellow), and necrotic (red) cells. (**b**) Quantitative analysis of the percentages of early apoptosis, late apoptosis, and necrosis. Data are presented as mean ± SD. *** *p* < 0.001, highly significant compared with the control group.

Apoptotic cell death induced by intermediate **A** in K562 cells was assessed using fluorescence-based Annexin V/ethidium homodimer staining. The analysis demonstrated a pronounced induction of apoptosis following treatment. Quantitative evaluation revealed that 62% of the cell population was in early apoptosis, while 25% progressed to late apoptosis. On the other hand, necrotic cells constituted only 13% of the total population. The predominance of early and late apoptotic cells (combined apoptotic fraction: 87%) indicates that intermediate **A** primarily triggers programmed cell death rather than nonspecific necrotic damage, suggesting apoptosis as the principal mechanism underlying its cytotoxic activity in K562 cells (Figure 6a,b).

**Figure 7:**
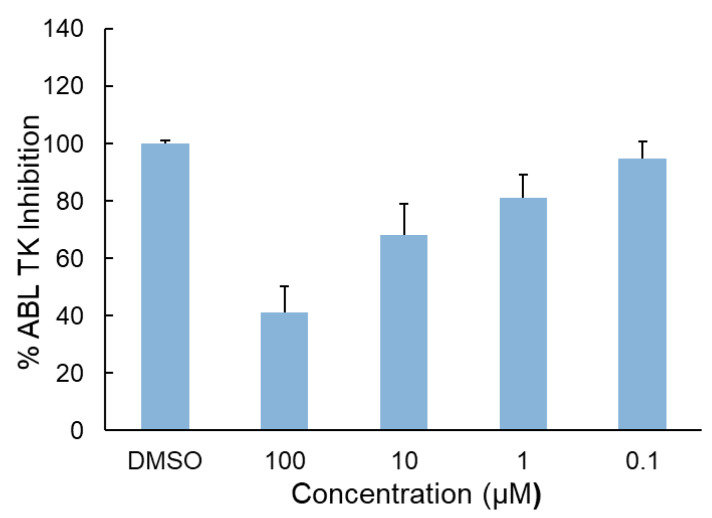
ABL TK inhibition by intermediate **A** at different concentrations.

The inhibitory activity of intermediate **A** against ABL TK was evaluated to determine its direct target engagement. As illustrated in Figure 7, intermediate **A** demonstrated a clear concentration-dependent inhibition of ABL TK activity. Increasing concentrations of the compound led to progressively enhanced kinase suppression, confirming a dose-responsive interaction with the enzyme. At 100 µM and 10 µM, intermediate **A** inhibited ABL TK activity by 59% and 32%, respectively. The calculated IC_50_ value was 49.05 ± 6.36 µM (Table 2), indicating moderate inhibitory potency toward the kinase.

### 2.3. In Silico Studies

**Figure 8:**
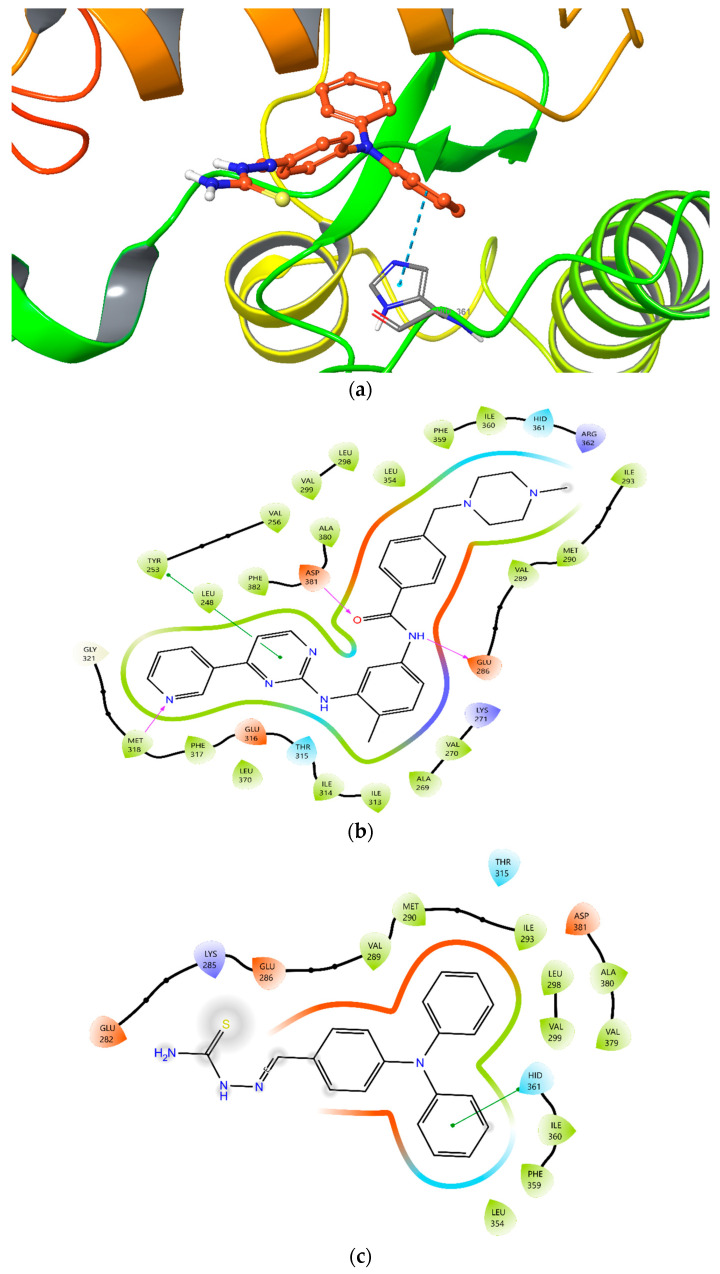
(**a**) Three-dimensional (3D) interaction diagram of intermediate **A** bound to ABL TK (Blue dashed lines represent π–π interactions) (**b**) Two-dimensional (2D) interaction diagram of imatinib (Purple lines represent hydrogen bonds; green lines represent π–π interactions) (**c**) 2D interaction diagram of intermediate **A** (Green lines represent π–π interactions).

A molecular docking study was performed to evaluate the binding mode of intermediate **A** within the ATP-binding site of ABL TK (PDB ID: 2HYY) [44], using the Maestro software platform [45], and compared with the reference inhibitor imatinib. The docking results indicated that intermediate **A** successfully occupied the ATP-binding pocket of ABL TK with a favorable binding orientation (Figure 8a). However, unlike imatinib, intermediate **A** did not establish several key interactions that are critical for high-affinity ABL TK inhibition, including contacts with Met318, Tyr253, Asp381, and Glu286 (Figure 8b). Instead, intermediate **A** formed a π-π stacking interaction with His361 through one of the phenyl rings of its diphenylamino moiety (Figure 8c). The docking score of intermediate **A** was calculated as −7.261 kcal/mol, whereas imatinib showed a significantly more favorable docking score of −12.273 kcal/mol, consistent with its established high binding affinity toward ABL TK. Intermediate **A** established a single aromatic interaction within the binding pocket: π–π interaction with His361 (Distance: 5.16 Å; Angle: 72.54°) (Table 3). No classical hydrogen bond interactions were observed for intermediate **A** under the selected docking conditions. Imatinib formed multiple stabilizing interactions: Hydrogen bond with Met318 (Distance: 1.54 Å; Angle: 113.76°), hydrogen bond with Asp381 (Distance: 2.24 Å; Angle: 114.88°), hydrogen bond with Glu286 (Distance: 1.98 Å; Angle: 150.21°) and a π-π interaction with Tyr253 (Distance: 4.91 Å; Angle: 78.84°) (Table 3). These interactions are consistent with the well-characterized ATP-competitive binding mode of imatinib.

The drug-likeness and pharmacokinetic properties of intermediate **A** were predicted using the SwissADME platform [46]. The compound displays a favorable physicochemical profile, with a molecular weight of 346.45 g/mol, TPSA of 85.74 Å^2^, two hydrogen-bond donors, and one hydrogen-bond acceptor, fully complying with Lipinski’s rule of five (0 violations). In addition, the molecule satisfies the Ghose, Veber, and Egan criteria, and its bioavailability score (0.55) supports acceptable oral bioavailability potential (Table 4).

**Figure 9:**
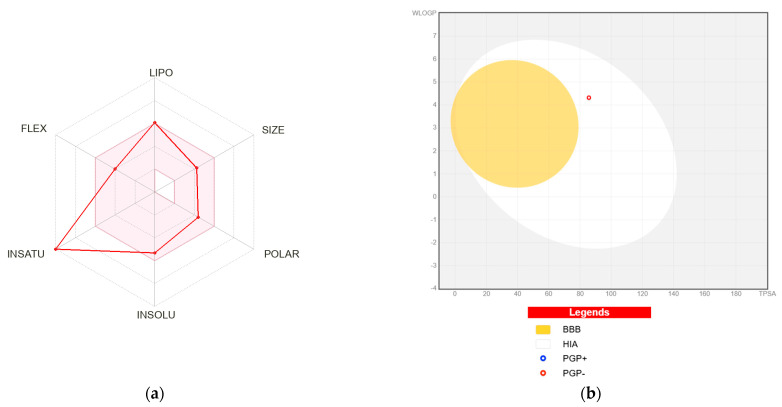
(**a**) SwissADME bioavailability radar and (**b**) BOILED-Egg diagram for intermediate **A**.

The SwissADME bioavailability radar further illustrates the compound’s drug-like characteristics across six key parameters: lipophilicity (LIPO), size (SIZE), polarity (POLAR), solubility (INSOLU), flexibility (FLEX), and saturation (INSATU) (Figure 9a). Most properties fall within the optimal pink region, indicating a generally suitable balance of physicochemical features for oral administration. The size and polarity parameters align well with the molecular weight and TPSA values, reinforcing the prediction of favorable gastrointestinal absorption.

Lipophilicity remains within acceptable limits (consensus LogP = 4.09), although the XLOGP3 value (5.10) approaches the upper boundary, accounting for the single violation in the Muegge and lead-likeness filters. The INSATU parameter extends beyond the recommended range due to the fully aromatic scaffold (fraction Csp^3^ = 0.00), indicating a planar and highly unsaturated structure. This high aromaticity may influence metabolic stability and solubility. Consistently, predicted aqueous solubility (logS values ranging from −5.34 to −6.67) suggests moderate-to-poor solubility, implying that formulation strategies or structural refinement may be necessary to improve dissolution behavior (Table 4).

Pharmacokinetic predictions indicate high gastrointestinal absorption and absence of blood–brain barrier (BBB) permeation (Figure 9b), which may be advantageous for non-CNS therapeutic applications. The compound is not predicted to be a P-gp substrate, potentially supporting adequate intracellular exposure. However, in silico CYP profiling suggests inhibition of CYP1A2, CYP2C19, and CYP2C9, highlighting possible drug–drug interaction risks that require experimental confirmation.

Importantly, no PAINS alerts were identified, supporting structural integrity and reducing the likelihood of assay interference. Although two Brenk alerts (imine and thiocarbonyl functionalities) were detected, the low synthetic accessibility score (2.89) suggests that the molecule is readily amenable to further medicinal chemistry optimization.

## 3. Discussion

Despite the transformative success of BCR::ABL1 TKIs in converting CML into a manageable chronic disease, important clinical limitations remain. Resistance development, long-term toxicity, cardiovascular adverse events, and persistence of leukemic stem cells continue to compromise durable therapeutic outcomes. These challenges highlight the need for structurally innovative ABL TK-targeting agents that combine efficacy with improved safety and alternative or complementary mechanisms of action.

Previous studies have shown that heterocyclic substitution within thiazole and hydrazone frameworks can enhance anti-CML activity and ABL TK inhibition. Based on this rationale, we designed *N*,*N*-diphenylaniline-based thiazolyl-hydrazone hybrids to combine the kinase-targeting potential of the thiazole ring with the versatile hydrogen-bonding capacity and favorable pharmacological properties of hydrazone linkers. The *N*,*N*-diphenyl aniline (diarylamine) scaffold was selected as a rational hydrophobic and aromatic module to fulfill the pharmacophoric requirements of ATP-site ABL TKIs. Clinically approved ABL TKIs, such as imatinib, nilotinib, ponatinib, and dasatinib, share a common design principle: a hinge-binding moiety linked to an extended hydrophobic region that occupies lipophilic subpockets within or adjacent to the ATP-binding site. The diarylamine core provides an extensive π-surface for hydrophobic and π-π interactions, conformational flexibility through rotatable aryl-*N* bonds, and multiple substitution sites suitable for systematic structure–activity relationship (SAR) optimization. Rather than directly mimicking existing drugs, this scaffold serves as a compact and modular aromatic anchor capable of probing key lipophilic regions of the ABL TK domain, while enabling rapid structural diversification to enhance potency and overcome resistance-related challenges.

For intermediate **A**, the presence of two -NH_2_ singlets at 6.47 and 7.18 ppm, an -NH signal at 10.09 ppm, and a -CH=N- singlet at 7.87 ppm confirmed the thiosemicarbazone framework. The ^13^C NMR spectrum supported this assignment, displaying a characteristic -CH=N- carbon at 150.2 ppm and a diagnostic C=S resonance at 177.9 ppm.

Successful cyclization to yield thiazole derivatives (**1**–**12**) was evidenced by disappearance of the -NH_2_ signals and loss of the C=S carbon resonance. Thiazole carbons appeared within their expected ranges (C_2_: 168.6–170.4 ppm; C_4_: 146.6–149.7 ppm; C_5_: 101.2–107.3 ppm), while -CH=N- carbons were observed at 141.5–143.8 ppm. Substituent-specific resonances further validated the proposed structures: methyl (compound **2**), methoxy (compound **3**), methylsulfonyl (compound **6**), and cyano (compound **8**). HRMS data were fully consistent with calculated molecular masses, confirming the structural integrity of all synthesized derivatives.

Intermediate **A** has previously been reported as a dual-channel fluorescent probe for Hg^2+^ and Ag^+^ detection in DMSO/H_2_O systems (pH 4.5) [42]. Additionally, its Cu(I) complex was shown to emit blue fluorescence and rapidly penetrate T24 human bladder cancer cells, where it localizes primarily in mitochondria, induces oxidative stress, and promotes mitochondrial dysfunction [43].

To our knowledge, the present work is the first to evaluate intermediate **A** in leukemia models and to investigate its apoptotic and ABL TK inhibitory effects in CML cells.

Among all synthesized molecules, intermediate **A** emerged as the most active compound. At 10 µM, it produced substantial cytotoxicity in K562 cells and demonstrated a clear dose-dependent antiproliferative effect, with an IC_50_ value of 6.15 ± 1.26 µM. Notably, intermediate **A** exhibited greater potency than the cyclized thiazolyl-hydrazone derivatives (**1**–**12**), suggesting that the thiosemicarbazone moiety plays a central role in mediating cellular growth inhibition. This finding indicates that structural rigidification through thiazole ring formation did not enhance biological activity within this series.

Intermediate **A** retained activity in HL-60 AML cells, albeit at reduced potency, suggesting partial selectivity toward BCR-ABL-dependent signaling pathways. The calculated selectivity index (SI = 12.9) further supports this observation, as intermediate **A** displayed lower toxicity toward healthy PBMCs compared with imatinib. Preservation of PBMC viability at intermediate concentrations suggests a potentially improved therapeutic window.

Flow cytometric analysis revealed that approximately 87% of treated K562 cells underwent early or late apoptosis, with minimal necrosis observed. This indicates that cytotoxicity is primarily mediated through regulated apoptotic pathways rather than nonspecific cellular damage. Such a mechanism aligns with the expected behavior of targeted anticancer agents and supports the biological relevance of intermediate **A**.

Although intermediate **A** demonstrated concentration-dependent inhibition of ABL TK activity, its enzymatic IC_50_ value (49.05 ± 6.36 µM) indicates moderate potency relative to classical ATP-competitive TKIs.

Molecular docking studies provided structural insight into the observed enzymatic activity profile. Intermediate **A** occupied the ATP-binding pocket; however, it failed to establish key hinge-region and catalytic hydrogen bonds typically observed for high-affinity ABL TKIs, particularly those involving Met318, Tyr253, Asp381, and Glu286. Instead, binding stabilization was primarily driven by a single π-π stacking interaction between the phenylamino moiety and His361. This limited interaction pattern is reflected in the docking score difference between intermediate **A** (−7.261 kcal/mol) and imatinib (−12.273 kcal/mol), indicating substantially weaker predicted binding affinity. These structural findings are consistent with the enzymatic inhibition data, where intermediate **A** demonstrated reduced ABL TK inhibitory potency relative to imatinib. Collectively, the docking results suggest that although intermediate **A** can access the ATP-binding pocket, the absence of critical hinge and catalytic residue interactions likely limits its binding efficiency, supporting the conclusion that ABL TK inhibition may not represent its primary mechanism of cytotoxic activity.

Intermediate **A** satisfied major drug-likeness criteria (Lipinski, Ghose, Veber, and Egan rules) and exhibited a favorable predicted bioavailability score (0.55). The bioavailability radar indicated that most physicochemical parameters reside within optimal ranges for orally active compounds.

However, elevated lipophilicity and high aromatic content contribute to limited predicted aqueous solubility (logS), which may negatively influence dissolution and pharmacokinetic behavior. These properties indicate opportunities for structural optimization, particularly by increasing molecular saturation and polarity while preserving target affinity. Although SwissADME predictions provide useful preliminary insight, they remain theoretical and require experimental validation. The compound was not predicted to be a P-gp substrate, which may favor intracellular exposure. Nevertheless, predicted inhibition of CYP1A2, CYP2C19, and CYP2C9 suggests potential metabolic liabilities and possible drug–drug interaction risks, whereas no inhibition was predicted for CYP2D6 and CYP3A4. Therefore, the ADME findings should be considered supportive but not confirmatory evidence of pharmacokinetic suitability. Importantly, no PAINS alerts were identified, and synthetic accessibility remains favorable.

Collectively, the present findings demonstrate that intermediate **A** exerts potent and selective cytotoxic effects in BCR-ABL-positive CML cells, primarily through apoptosis induction, while maintaining a comparatively favorable safety profile in normal PBMCs. Although direct ABL TK inhibition is moderate, its overall biological profile suggests a multifactorial mechanism of action.

Future optimization efforts should focus on: enhancing hinge-region hydrogen bonding to improve kinase affinity, increasing molecular saturation to reduce excessive lipophilicity and improving aqueous solubility while preserving cellular selectivity.

Overall, thiazolyl-hydrazone-related scaffolds, particularly thiosemicarbazone-containing intermediate, represent significant starting points for the development of next-generation CML therapeutics aimed at overcoming resistance and safety limitations associated with current TKIs.

## 4. Materials and Methods

### 4.1. Chemistry

All reagents and solvents were obtained from reputable commercial suppliers and used without further purification unless otherwise noted. Melting points were determined using a Mettler Toledo MP90 digital apparatus (Toledo, OH, USA) and are reported uncorrected. The progress of reactions and the purity of synthesized compounds were monitored by thin-layer chromatography (TLC) on Merck silica gel 60 F254 plates (Darmstadt, Germany). Proton (^1^H) and carbon (^13^C) NMR spectra were recorded on a Bruker NMR spectrometer (Bruker, Billerica, MA, USA). HRMS data were acquired using a JEOL JMS-700 Station/JMS-BU-20-GCmate system (JEOL, Akishima, Tokyo, Japan).

#### 4.1.1. Synthesis of Intermediate **A**

An equimolar mixture of 4-(*N*,*N*-diphenylamino)benzaldehyde (0.025 mol) and thiosemicarbazide (0.025 mol) was dissolved in 40 mL of ethanol and refluxed for 12 h. After completion of the reaction, the solution was allowed to cool to rt, resulting in the formation of a solid precipitate. The precipitate was collected by filtration, thoroughly washed, and dried under reduced pressure. The crude product was further purified by recrystallization from ethanol to yield intermediate **A** in analytically pure form, in accordance with previously reported methods [47].

2-(4-(Diphenylamino)benzylidene)hydrazine-1-carbothioamide (A) [42,43]: Yellow solid. Yield: 87%. M.p. 204–205 °C. ^1^H NMR (500 MHz, *CDCl*_3_) *δ* (ppm): 6.47 (s, 1H), 7.00 (d, *J* = 9.1 Hz, 2H), 7.08–7.13 (m, 6H), 7.18 (s, 1 H), 7.26–7.30 (m, 4H), 7.47 (d, *J* = 8.5 Hz, 2 H), 7.87 (s, 1H), 10.09 (s, 1H). ^13^C NMR (125 MHz, *CDCl*_3_) *δ* (ppm): 121.7 (2CH), 124.3 (2CH), 125.4 (4CH), 128.6 (C), 129.6 (6CH), 144.1 (C), 146.9 (2C), 150.2 (CH), 177.9 (C). HRMS (FAB) calcd. for C_20_H_18_N_4_S [M + H]^+^: (*m*/*z*) = 347.1330; found: 347.1323. (Spectral Data: Supplementary Information. Figures S1–S3).

#### 4.1.2. Synthesis of Compounds **1**–**12**

Intermediate **A** (0.001 mol) was reacted with the corresponding 2-bromo-1-arylethanone derivative (0.001 mol) in 20 mL of ethanol, and the mixture was refluxed for 6 h. After cooling to room temperature, a solid precipitate formed and was collected by filtration. The crude product was then purified by recrystallization from ethanol to afford the desired thiazole derivative in analytically pure form, following previously reported procedures [47].

*N*,*N*-Diphenyl-4-((2-(4-phenylthiazol-2-yl)hydrazineylidene)methyl)aniline (**1**): Brown solid. Yield: 72%. M.p. 261–262 °C. ^1^H NMR (500 MHz, DMSO-*d*_6_) *δ* (ppm): 6.92 (d, *J* = 7.8 Hz, 1H), 6.95–7.01 (m, 3H), 7.04 (d, *J* = 7.2 Hz, 2H), 7.09 (d, *J* = 7.2 Hz, 2H), 7.11 (t, *J* = 6.6 Hz, 1H), 7.18 (d, *J* = 8.8 Hz, 1H), 7.22–7.28 (m, 2H), 7.29–7.33 (m, 3H), 7.40 (d, *J* = 7.8 Hz, 1H), 7.42 (t, *J* = 7.8 Hz, 1H), 7.49 (d, *J* = 8.7 Hz, 1H), 7.55 (d, *J* = 8.3 Hz, 1H), 7.85 (d, *J* = 8.5 Hz, 1H), 7.99 (s, 1H). ^13^C NMR (125 MHz, *CDCl*_3_) *δ* (ppm): 102.9 (CH), 122.3 (2CH), 123.6 (2CH), 125.1 (4CH), 126.4 (2CH), 127.8 (2CH), 129.1 (2CH), 129.4 (5CH), 133.6 (C), 133.6 (C), 142.8 (CH), 147.3 (3C), 149.1 (C), 170.1 (C). HRMS (FAB) calcd. for C_28_H_22_N_4_S [M + H]^+^: (*m*/*z*) = 447.1643; found: 447.1629. (Spectral Data: Supplementary Information, Figures S4–S6).

*N*,*N*-Diphenyl-4-((2-(4-(p-tolyl)thiazol-2-yl)hydrazineylidene)methyl)aniline (**2**): Brown solid. Yield: 78%. M.p. 251–252 °C. ^1^H NMR (500 MHz, *CDCl*_3_) *δ* (ppm): 2.33 (s, 3H), 6.69 (s, 1H), 6.95 (d, *J* = 9.1 Hz, 2H), 7.05 (t, *J* = 7.2 Hz, 3H), 7.09 (d, *J* = 8.7 Hz, 4H), 7.17–7.19 (m, 3H), 7.26 (t, *J* = 7.5 Hz, 4H), 7.33 (s, 1H), 7.67 (d, *J* = 7.9 Hz, 2H). ^13^C NMR (125 MHz, *CDCl*_3_) *δ* (ppm): 21.7 (CH_3_), 102.1 (CH), 122.3 (2CH), 123.7 (2CH), 125.1 (6CH), 126.5 (2CH), 127.8 (2CH), 129.4 (4CH), 129.7 (C), 131.0 (C), 138.5 (C), 143.8 (CH), 147.2 (2C), 149.2 (C), 149.4 (C), 170.1 (C). HRMS (FAB) calcd. for C_29_H_24_N_4_S [M + H]^+^: (*m*/*z*) = 461.1800; found: 461.1787. (Spectral Data: Supplementary Information, Figures S7–S9).

*N*,*N*-Diphenyl-4-((2-(4-(4-methoxyphenyl)thiazol-2-yl)hydrazineylidene)methyl)aniline (**3**): Dark red solid. Yield: 76%. M.p. 266–267 °C. ^1^H NMR (500 MHz, *CDCl*_3_) *δ* (ppm): 3.70 (s, 3H), 6.61 (s, 1H), 6.87 (d, *J* = 9.9 Hz, 2H), 6.94 (d, *J* = 9.4 Hz, 2H), 7.04 (t, *J* = 7.2 Hz, 2H), 7.08 (d, *J* = 8.9 Hz, 4H), 7.17 (d, *J* = 9.4 Hz, 2H), 7.24 (d, *J* = 9.9 Hz, 3H), 7.27 (s, 2H), 7.70 (d, *J* = 8.8 Hz, 2H). ^13^C NMR (125 MHz, *CDCl*_3_) *δ* (ppm): 55.3 (CH_3_), 101.2 (CH), 114.2 (2CH), 122.4 (2CH), 123.7 (2CH), 125.0 (6CH), 125.9 (C), 126.8 (C), 127.9 (2CH), 129.5 (4CH), 143.5 (CH), 147.3 (2C), 149.2 (C), 149.4 (C), 159.7 (C), 170.3 (C). HRMS (FAB) calcd. for C_29_H_24_N_4_OS [M + H]^+^: (*m*/*z*) = 477.1749; found: 477.1743. (Spectral Data: Supplementary Information, Figures S10–S12).

*N*,*N*-diphenyl-4-((2-(4-(4-(trifluoromethyl)phenyl)thiazol-2-yl)hydrazineylidene)methyl)aniline (**4**): Green solid. Yield: 82%. M.p. 247–248 °C. ^1^H NMR (500 MHz, *CDCl*_3_) *δ* (ppm): 6.9 (s, 1H), 6.90 (d, *J* = 9.1 Hz, 2H), 7.05 (d, *J* = 7.5 Hz, 2H), 7.07–7.11 (m, 3H), 7.15 (d, *J* = 8.4 Hz, 2H), 7.22 (d, *J* = 7.5 Hz, 2H), 7.26 (t, *J* = 7.7 Hz, 4H), 7.60 (d, *J* = 7.9 Hz, 2H), 7.89 (d, *J* = 7.9 Hz, 2H). ^13^C NMR (125 MHz, *CDCl*_3_) *δ* (ppm): 105.2 (CH), 122.6 (2CH), 123.7 (2CH), 124.1 (C), 125.1 (4CH), 125.5 (2CH), 125.8 (2CH), 126.5 (2CH), 127.6 (C), 129.5 (4CH), 133.3 (C), 137.9 (C), 142.9 (CH), 147.2 (3C), 149.3 (C), 170.4 (C). HRMS (FAB) calcd. for C_29_H_21_F_3_N_4_S [M + H]^+^: (*m*/*z*) = 514.1439; found: 514.1442. (Spectral Data: Supplementary Information, Figures S13–S15).

*N*,*N*-Diphenyl-4-((2-(4-(4-(trifluoromethoxy)phenyl)thiazol-2-yl)hydrazineylidene)methyl)aniline (**5**): Dark red solid. Yield: 84%. M.p. 246–247 °C. ^1^H NMR (500 MHz, *CDCl*_3_) *δ* (ppm): 6.82 (s, 1H), 6.96 (d, *J* = 8.6 Hz, 2H), 7.05 (d, *J* = 7.5 Hz, 2H), 7.07–7.10 (m, 4H), 7.19–7.23 (m, 5 H), 7.26 (t, *J* = 7.1 Hz, 3H), 7.30 (s, 1H), 7.82 (d, *J* = 8.8 Hz, 2H). ^13^C NMR (125 MHz, *CDCl*_3_) *δ* (ppm): 103.7 (CH), 121.2 (2CH), 122.5 (2CH), 123.6 (2CH), 124.7 (C), 125.1 (4CH), 127.3 (C), 127.5 (2CH), 127.6 (2CH), 129.4 (4CH), 133.4 (C), 142.7 (CH), 147.1 (3C), 149.2 (C), 149.4 (C), 170.2 (C). HRMS (FAB) calcd. for C_29_H_21_F_3_N_4_OS [M + H]^+^: (*m*/*z*) = 530.1388; found: 530.1390. (Spectral Data: Supplementary Information, Figures S16–S18).

*N*,*N*-Diphenyl-4-((2-(4-(4-(methylsulfonyl)phenyl)thiazol-2-yl)hydrazineylidene)methyl)aniline (**6**): Pale green solid. Yield: 86%. M.p. 205–206 °C. ^1^H NMR (500 MHz, DMSO-*d_6_*) *δ* (ppm): 3.38 (s, 3H), 6.97 (d, *J* = 8.3 Hz, 2H), 7.08 (d, *J* = 9.2 Hz, 4H), 7.12 (t, *J* = 8.2 Hz, 2H), 7.35 (t, *J* = 8.2 Hz, 4H), 7.56 (d, *J* = 8.9 Hz, 2H), 7.60 (s, 1H), 7.97 (d, *J* = 8.2 Hz, 2H), 7.99 (s, 1H), 8.11 (d, *J* = 8.6 Hz, 2H), 12.16 (s, 1H). ^13^C NMR (125 MHz, DMSO-*d_6_*) *δ* (ppm): 43.7 (CH_3_), 107.2 (CH), 121.9 (2CH), 123.7 (2CH), 124.7 (5CH), 126.1 (2CH), 127.5 (CH), 127.7 (CH), 127.8 (C), 129.7 (5CH), 139.1 (2C), 141.5 (CH), 146.6 (C), 148.3 (3C), 168.6 (C). HRMS (FAB) calcd. for C_29_H_24_N_4_O_2_S_2_ [M + H]^+^: (*m*/*z*) = 524.1341; found: 524.1325. (Spectral Data: Supplementary Information, Figures S19–S21).

*N*,*N*-Diphenyl-4-((2-(4-(4-nitrophenyl)thiazol-2-yl)hydrazineylidene)methyl)aniline (**7**): Brown solid. Yield: 70%. M.p. 292–293 °C. ^1^H NMR (500 MHz, *CDCl*_3_) *δ* (ppm): 6.97 (d, *J* = 8.8 Hz, 2H), 7.06–7.11 (m, 7H), 7.25–7.30 (m, 6H), 7.46 (s, 1H), 7.94 (d, *J* = 9.1 Hz, 2H), 8.21 (d, *J* = 9.6 Hz, 2H). ^13^C NMR (125 MHz, *CDCl*_3_) *δ* (ppm): 107.3 (CH), 122.1 (2CH), 123.8 (2CH), 124.2 (2CH), 125.3 (5CH), 126.7 (2CH), 127.6 (C), 129.5 (5CH), 140.6 (C), 142.7 (CH), 147.0 (2C), 148.9 (C), 149.5 (C), 151.4 (C), 169.6 (C). HRMS (FAB) calcd. for C_28_H_21_N_5_O_2_S [M + H]^+^: (*m*/*z*) = 491.1416; found: 491.1395. (Spectral Data: Supplementary Information, Figures S22–S24).

*N*,*N*-Diphenyl-4-((2-(4-(4-cyanophenyl)thiazol-2-yl)hydrazineylidene)methyl)aniline (**8**): Green solid. Yield: 71%. M.p. 273–274 °C. ^1^H NMR (500 MHz, DMSO-*d*_6_) *δ* (ppm): 6.97 (d, *J* = 9.3 Hz, 2H), 7.08 (d, *J* = 7.5 Hz, 4H), 7.12 (t, *J* = 8.4 Hz, 2H), 7.35 (t, *J* = 7.9 Hz, 4H), 7.55 (d, *J* = 9.8 Hz, 2H), 7.61 (s, 1H), 7.87 (d, *J* = 9.3 Hz, 2H), 7.99 (s, 1H), 8.04 (d, *J* = 8.9 Hz, 2H), 12.10 (s, 1H). ^13^C NMR (125 MHz, DMSO-*d_6_*) *δ* (ppm): 107.3 (CH), 109.8 (C), 119.1 (C), 121.9 (2CH), 123.8 (2CH), 124.7 (5CH), 126.1 (2CH), 127.6 (2CH), 129.7 (5CH), 132.7 (C), 138.8 (C), 141.6 (CH), 146.7 (3C), 148.3 (C), 168.7 (C). HRMS (FAB) calcd. for C_29_H_21_N_5_S [M + H]^+^: (*m*/*z*) = 471.1518; found: 471.1507. (Spectral Data: Supplementary Information, Figures S25–S27).

*N*,*N*-Diphenyl-4-((2-(4-(4-fluorophenyl)thiazol-2-yl)hydrazineylidene)methyl)aniline (**9**): Dark red solid. Yield: 80%. M.p. 254–255 °C. ^1^H NMR (500 MHz, *CDCl*_3_) *δ* (ppm): 6.72 (s, 1H), 6.95 (d, *J* = 8.7 Hz, 2H), 7.05 (t, *J* = 7.3 Hz, 4H), 7.09 (d, *J* = 6.9 Hz, 4H), 7.16 (d, *J* = 8.4 Hz, 2H), 7.22 (s, 2H), 7.26 (t, *J* = 7.3 Hz, 3H), 7.75–7.78 (m, 2H). ^13^C NMR (125 MHz, *CDCl*_3_) *δ* (ppm): 102.8 (CH), 115.8 (d, *J* = 21.0 Hz, 2CH), 122.3 (2CH), 123.6 (2CH), 125.1 (4CH), 127.4 (C), 127.6 (3CH), 128.1 (d, *J* = 12.9 Hz, C), 129.4 (5CH), 142.6 (CH), 147.3 (2C), 149.1 (C), 149.6 (C), 161.6 (C), 170.2 (C). HRMS (FAB) calcd. for C_28_H_21_FN_4_S [M + H]^+^: (*m*/*z*) = 465.1549; found: 465.1544. (Spectral Data: Supplementary Information, Figures S28–S30).

*N*,*N*-Diphenyl-4-((2-(4-(4-chlorophenyl)thiazol-2-yl)hydrazineylidene)methyl)aniline (**10**): Brown solid. Yield: %79. M.p. 259–260 °C. ^1^H NMR (500 MHz, *CDCl*_3_) *δ* (ppm): 6.79 (s, 1H), 6.98 (d, *J* = 9.5 Hz, 2H), 7.03 (t, *J* = 6.8 Hz, 2H), 7.10 (d, *J* = 8.9 Hz, 5H), 7.17 (d, *J* = 8.4 Hz, 2H), 7.26 (t, *J* = 7.4 Hz, 4H), 7.33 (d, *J* = 9.5 Hz, 2H), 7.73 (d, *J* = 7.7 Hz, 2H). ^13^C NMR (125 MHz, *CDCl*_3_) *δ* (ppm): 103.9 (CH), 122.6 (2CH), 123.9 (2CH), 124.9 (4CH), 127.3 (C), 127.6 (2CH), 127.7 (2CH), 129.0 (2CH), 129.4 (4CH), 133.3 (C), 133.8 (C), 142.5 (CH), 147.3 (2C), 149.1 (C), 149.7 (C), 170.3 (C). HRMS (FAB) calcd. for C_28_H_21_ClN_4_S [M + H]^+^: (*m*/*z*) = 480.1175; found: 480.1171. (Spectral Data: Supplementary Information, Figures S31–S33).

*N*,*N*-Diphenyl-4-((2-(4-(4-bromophenyl)thiazol-2-yl)hydrazineylidene)methyl)aniline (**11**): Brown solid. Yield: 71%. M.p. 256–257 °C. ^1^H NMR (500 MHz, *CDCl*_3_) *δ* (ppm): 6.78 (s, 1H), 6.98 (d, *J* = 9.7 Hz, 2H), 7.05 (d, *J* = 8.4 Hz, 2H), 7.10 (d, *J* = 8.4 Hz, 4H), 7.18 (d, *J* = 8.5 Hz, 2H), 7.22–7.25 (m, 3H), 7.27 (d, *J* = 7.2 Hz, 2H), 7.47 (d, *J* = 8.5 Hz, 2H), 7.64 (d, *J* = 9.3 Hz, 2H). ^13^C NMR (125 MHz, *CDCl*_3_) *δ* (ppm): 103.7 (CH), 122.5 (2CH), 123.8 (C), 125.2 (4CH), 127.1 (C), 127.8 (2CH), 127.9 (2CH), 126.5 (2CH), 127.6 (2CH), 147.3 (3C), 129.5 (4CH), 132.0 (C), 143.4 (CH), 149.1 (C), 170.2 (C). HRMS (FAB) calcd. for C_28_H_21_BrN_4_S [M + H]^+^: (*m*/*z*) = 525.0749; found: 525.0697. (Spectral Data: Supplementary Information, Figures S34–S36).

*N*,*N*-Diphenyl-4-((2-(4-(3-bromophenyl)thiazol-2-yl)hydrazineylidene)methyl)aniline (**12**): Red solid. Yield: 77%. M.p. 260–261 °C. ^1^H NMR (500 MHz, *CDCl*_3_) *δ* (ppm): 6.82 (s, 1H), 6.98 (d, *J* = 9.0 Hz, 2H), 7.06 (t, *J* = 7.3 Hz, 2H), 7.10 (d, *J* = 8.4 Hz, 4H), 7.22–7.29 (m, 7H), 7.35 (s, 1H), 7.40 (d, *J* = 7.7 Hz, 1H), 7.73 (d, *J* = 7.7 Hz, 1H), 7.94 (s, 1H). ^13^C NMR (125 MHz, *CDCl*_3_) *δ* (ppm): 104.4 (CH), 122.3 (C), 123.7 (2CH), 124.8 (2CH), 125.2 (5CH), 127.4 (C), 127.8 (2CH), 129.5 (5CH), 130.5 (CH), 131.0 (CH), 136.7 (C), 142.7 (CH), 147.3 (2C), 149.1 (C), 149.2 (C), 170.0 (C). HRMS (FAB) calcd. for C_28_H_21_BrN_4_S [M + H]^+^: (*m*/*z*) = 525.0749; found: 525.0704. (Spectral Data: Supplementary Information, Figures S37–S39).

### 4.2. Biologic Activity

#### 4.2.1. Cytotoxicity

Cell culture preparation and treatment with test compounds: K562 (ATCC, Manassas, VA, USA) and HL-60 (ATCC, Manassas, VA, USA) cell lines, along with peripheral blood mononuclear cells (PBMCs) obtained from Precision Bioservices (Frederick, MD, USA), were cultured in RPMI 1640 medium (Wako Pure Chemical Industries, Osaka, Japan). The medium was supplemented with 5% fetal bovine serum (FBS) (Sigma-Aldrich, St. Louis, MO, USA) and 89 μg/mL streptomycin (Meiji Seika Pharma, Tokyo, Japan). Cells were maintained at 37 °C in a humidified incubator containing 95% air and 5% CO_2_. Exponentially growing cancer cells were seeded into 96-well plates (for PBMCs) and 24-well tissue culture plates at densities of 4 × 10^4^ cells/mL for K562 and HL-60 cells, and 1 × 10^6^ cells/mL for PBMCs. After treatment with the test compounds, cells were incubated for 48 h. Optimal cell densities and incubation periods for cytotoxicity assessment had been established in advance. Stock solutions of the synthesized compounds and imatinib (0.1, 0.3, 1, 3, and 10 mM) were prepared in DMSO (Wako Pure Chemical Industries) and subsequently diluted with fresh culture medium. The final concentration of DMSO in all samples was maintained at 1% [48,49,50].

Cytotoxicity assessment: Cell viability was evaluated using the MTT assay, based on the reduction of 3-(4,5-dimethylthiazol-2-yl)-2,5-diphenyltetrazolium bromide, as previously described in the literature [48,49,50,51], with slight modifications. Cells were exposed to various concentrations of the test compounds for 48 h. Following treatment, MTT solution was added and the cells were incubated for an additional 4 h at 37 °C. The medium was then carefully removed, and the resulting formazan crystals were dissolved in 100 µL of DMSO. Absorbance was measured at 570 nm using an Infinite M1000 multimode microplate reader (Tecan, Grödig, Austria).

All experiments were performed in triplicate. IC_50_ values were calculated as the concentration of compound required to reduce absorbance by 50%. Imatinib, an ABL TKI with established anticancer activity, served as the positive control.

#### 4.2.2. Apoptosis Assay

K562 cells were exposed to intermediate **A** at its IC_50_ concentration and incubated for 12 h. After treatment, the cells were harvested using 0.05% trypsin and washed twice with 1× binding buffer prior to fluorescence-based apoptosis analysis. The cell pellet was resuspended in a staining solution containing 50 µL of 1× binding buffer and 4 µL each of FITC-Annexin V, ethidium homodimer III, and Hoechst 33,342. The mixture was incubated for 20 min at room temperature in the dark to allow adequate staining. Following incubation, the cells were washed again with binding buffer, fixed in 2% paraformaldehyde, and briefly rinsed with phosphate-buffered saline (PBS). Apoptotic and necrotic cells were then visualized and documented using a Biorevo BZ-9000 fluorescence microscope (Keyence, Osaka, Japan), according to previously reported procedures [48,49,50].

#### 4.2.3. ABL TK Inhibition Assay

The inhibitory activity of intermediate **A** against ABL TK was evaluated and compared with that of imatinib. The assay was performed using the Promega ABL TK enzyme system (Promega V1901, Promega Corporation, Madison, WI, USA). All kinase reactions were conducted in a reaction buffer containing 40 mM Tris (pH 7.5), 20 mM MgCl_2_, and 0.1 mg/mL BSA, supplemented with 10 mM ATP and 2 mM DTT as a cofactor. An ABL TK stock solution was prepared at a final concentration of 4 ng/µL using 2.5× kinase buffer and purified water. An Abltide substrate (EAIYAAPFAKKK) stock solution was also prepared at a final concentration of 0.2 µg/µL substrate with 25 µM ATP, using 2.5× kinase buffer and a 100 µM ATP solution. Kinase reactions were assembled in 384-well plates by combining 4 µL of ABL TK working solution, 4 µL of ATP/substrate working solution, and 2 µL of either compound solution (at 10 µM or 1 µM) or 5% DMSO as a control. The reaction mixtures were incubated at room temperature for 2 h. ABL TK activity was subsequently measured using the ADP-Glo™ Kinase Detection Assay (Promega Corporation, Madison, WI, USA) following the manufacturer’s instructions. Briefly, 5 µL of ADP-Glo reagent was added to each well and incubated for 40 min at room temperature to terminate the kinase reaction and deplete residual ATP. Next, 10 µL of Kinase Detection Reagent was added, followed by an additional 30 min incubation to convert ADP to ATP and generate a luminescent signal. In dose–response experiments, kinase inhibition by intermediate **A** and imatinib was determined at concentrations of 100 µM, 10 µM, 1 µM, and 0.1 µM using an Infinite M1000 multimode microplate reader (Tecan, Grödig, Austria). The percentage inhibition of ABL TK activity was then calculated [48,49,50].

### 4.3. In Silico Studies

The crystal structure of ABL TK was obtained from the RCSB Protein Data Bank (PDB ID: 2HYY) [44]. Protein preparation was performed using the PrepWizard module in Maestro [45], where missing chains were added with Prime and protonation states at physiological pH were assigned using PropKa. The receptor-ligand complex was then energy-minimized employing the OPLS 2005 force field. Intermediate **A** and imatinib were drawn and geometrically optimized within the Maestro interface, followed by preparation using the LigPrep module at physiological pH. Energy minimization was carried out using the same OPLS 2005 force field. Grid generation and molecular docking studies were conducted with Glide in standard precision (SP) mode for intermediate **A** and imatinib [45,48,50]. Additionally, the SwissADME online tool [46] was used to estimate the pharmacokinetic properties of intermediate **A**.

## 5. Conclusions

The present study identifies *N*,*N*-diphenylaniline-based intermediate (**A**) and its new thiazolyl-hydrazone derivatives (**1**–**12**) as significant scaffolds for the development of novel anti-CML agents. Among the synthesized compounds, intermediate **A** exhibited pronounced antiproliferative activity in BCR-ABL-positive leukemia cells, accompanied by significant apoptosis induction and enhanced selectivity over healthy PBMCs, suggesting a favorable therapeutic window. Although its direct ABL TK inhibition was moderate relative to classical ATP-competitive inhibitors, docking analysis indicated a feasible binding orientation within the ATP-binding pocket. However, the notable discrepancy between enzymatic and cellular IC_50_ values suggests that ABL TK inhibition may not be the sole determinant of the compound’s cellular activity. Consequently, the integration of *N*,*N*-diphenylaniline moiety provides a multifunctional framework with drug-like characteristics and clear opportunities for further structural optimization. These findings establish intermediate **A** as a valuable candidate for the development of next-generation therapeutics aimed at overcoming current limitations in CML treatment.

## Figures and Tables

**Table 1:** IC_50_ values and selectivity indices (SI) of intermediate **A** and imatinib.

Compound	IC_50_ Values (μM)			SI *
	K562	HL-60	PBMCs	
Intermediate **A**	6.15 ± 1.26	12.04 ± 1.70	79.31 ± 10.05	12.9
Imatinib	5.14 ± 1.14	12.55 ± 1.89	32.02 ± 5.42	6.2

* Selectivity Index (SI) = IC_50_ for PBMC/IC_50_ for K562 cell line.

**Table 2:** IC_50_ value of intermediate **A** for ABL TK inhibition.

Compound	IC_50_ Values (µM)
	ABL-1 Inhibition
Intermediate **A**	49.05 ± 6.36

**Table 3:** Ligand-residue interactions of intermediate **A** and imatinib.

Ligand	Residue	Interaction Type	Distance (Å)	Angle (°)
Intermediate **A**	His361	π–π	5.16	72.54
Imatinib	Met318	H-bond	1.54	113.76
Imatinib	Asp381	H-bond	2.24	114.88
Imatinib	Glu286	H-bond	1.98	150.21
Imatinib	Tyr253	π–π	4.91	78.84

**Table 4:** SwissADME-predicted physicochemical and pharmacokinetic properties of intermediate **A**.

Category	Parameter	Value	Parameter	Value	Parameter	Value
Physicochemical	Molar Refractivity	107.88	Fraction Csp3	0.00	Heavy Atoms	25
	Aromatic Heavy Atoms	18	H-Bond Donors	2	Rotatable Bonds	6
	H-Bond Acceptors	1			TPSA	85.74 Å^2^
Lipophilicity	iLOGP	3.32	XLOGP3	5.10	WLOGP	4.32
	MLOGP	3.77	SILICOS-IT	3.94	Consensus LogP	4.09
Solubility	ESOL LogS	−5.34 (Mod.)	Ali LogS	−6.64 (Poor)	SILICOS-IT LogS	−6.67 (Poor)
Pharmacokinetics	GI Absorption	High	BBB Permeation	No	P-gp Substrate	No
	CYP1A2 Inhibitor	Yes	CYP2C19 Inhibitor	Yes	CYP2C9 Inhibitor	Yes
	CYP2D6 Inhibitor	No	CYP3A4 Inhibitor	No	Log Kp	−4.79 cm/s
Drug-likeness	Lipinski	Yes	Ghose	Yes	Veber	Yes
	Egan	Yes	Muegge	No (XLOGP3 > 5)	Bioavailability Score	0.55
Medicinal Chemistry	PAINS	0	Brenk Alerts	2	Lead-likeness	No
	Synthetic Accessibility	2.89				

## Data Availability

The original contributions presented in this study are included in the article/Supplementary Material. Further inquiries can be directed to the corresponding author.

## Supplementary Materials

The following supporting information can be downloaded at: https://www.mdpi.com/article/10.3390/ph19030416/s1, Figure S1: ^1^H NMR Spectrum of intermediate **A**; Figure S2: ^13^C NMR Spectrum of intermediate **A**; Figure S3: Mass Spectrum of intermediate **A**; Figure S4: ^1^H NMR Spectrum of compound **1**; Figure S5: ^13^C NMR Spectrum of compound **1**; Figure S6: Mass Spectrum of compound **1**; Figure S7: ^1^H NMR Spectrum of compound **2**; Figure S8: ^13^C NMR Spectrum of compound **2**; Figure S9: Mass Spectrum of compound **2**; Figure S10: ^1^H NMR Spectrum of compound **3**; Figure S11: ^13^C NMR Spectrum of compound **3**; Figure S12: Mass Spectrum of compound **3**; Figure S13: ^1^H NMR Spectrum of compound **4**; Figure S14: ^13^C NMR Spectrum of compound **4**; Figure S15: Mass Spectrum of compound **4**; Figure S16: ^1^H NMR Spectrum of compound **5**; Figure S17: ^13^C NMR Spectrum of compound **5**; Figure S18: Mass Spectrum of compound **5**; Figure S19: ^1^H NMR Spectrum of compound **6**; Figure S20: ^13^C NMR Spectrum of compound **6**; Figure S21: Mass Spectrum of compound **6**; Figure S22: ^1^H NMR Spectrum of compound **7**; Figure S23: ^13^C NMR Spectrum of compound **7**; Figure S24: Mass Spectrum of compound **7**; Figure S25: ^1^H NMR Spectrum of compound **8**; Figure S26: ^13^C NMR Spectrum of compound **8**; Figure S27: Mass Spectrum of compound **8**; Figure S28: ^1^H NMR Spectrum of compound **9**; Figure S29: ^13^C NMR Spectrum of compound **9**; Figure S30: Mass Spectrum of compound **9**; Figure S31: ^1^H NMR Spectrum of compound **10**; Figure S32: ^13^C NMR Spectrum of compound **10**; Figure S33: Mass Spectrum of compound **10**; Figure S34: ^1^H NMR Spectrum of compound **11**; Figure S35: ^13^C NMR Spectrum of compound **11**; Figure S36: Mass Spectrum of compound **11**; Figure S37: ^1^H NMR Spectrum of compound **12**; Figure S38: ^13^C NMR Spectrum of compound **12**; Figure S39: Mass Spectrum of compound **12**.
