# Supplementary material for: Design, Synthesis, and Biological Evaluation of N,N-Diphenylaniline-Based Derivatives as Antiproliferative Agents and ABL TK Inhibitors Against CML
Source: Pharmaceuticals (Basel). 2026 Mar 4;19(3):416. doi: 10.3390/ph19030416 (PMC13028716; doi:10.3390/ph19030416)

Supplementary Information

Design, Synthesis, and Biological Evaluation of *N,N*-Diphenylaniline-Based Derivatives as Antiproliferative Agents and ABL TK Inhibitors against CML

Belgin Sever [1,2] and Halilibrahim Ciftci [2,*]

[1] Department of Pharmaceutical Chemistry, Faculty of Pharmacy, Anadolu University, Eskisehir 26470, Türkiye; belginsever@anadolu.edu.tr (B.S.)
[2] Department of Molecular Biology and Genetics, Burdur Mehmet Akif Ersoy University, Istiklal Campus, Burdur 15200, Türkiye; hciftci@mehmetakif.edu.tr (H.C.)

* Correspondence: hciftci@mehmetakif.edu.tr (H.C.)

.

Supplementary Figures

Figure S1: ^{1}H NMR Spectrum of intermediate **A**
Figure S2: ^{13}C NMR Spectrum of intermediate **A**
Figure S3: Mass Spectrum of intermediate **A**
Figure S4: ^{1}H NMR Spectrum of compound **1**
Figure S5: ^{13}C NMR Spectrum of compound **1**
Figure S6: Mass Spectrum of compound **1**
Figure S7: ^{1}H NMR Spectrum of compound **2**
Figure S8: ^{13}C NMR Spectrum of compound **2**
Figure S9: Mass Spectrum of compound **2**
Figure S10: ^{1}H NMR Spectrum of compound **3**
Figure S11: ^{13}C NMR Spectrum of compound **3**
Figure S12: Mass Spectrum of compound **3**
Figure S13: ^{1}H NMR Spectrum of compound **4**
Figure S14: ^{13}C NMR Spectrum of compound **4**
Figure S15: Mass Spectrum of compound **4**
Figure S16: ^{1}H NMR Spectrum of compound **5**
Figure S17: ^{13}C NMR Spectrum of compound **5**
Figure S18: Mass Spectrum of compound **5**
Figure S19: ^{1}H NMR Spectrum of compound **6**
Figure S20: ^{13}C NMR Spectrum of compound **6**
Figure S21: Mass Spectrum of compound **6**
Figure S22: ^{1}H NMR Spectrum of compound **7**
Figure S23: ^{13}C NMR Spectrum of compound **7**
Figure S24: Mass Spectrum of compound **7**
Figure S25: ^{1}H NMR Spectrum of compound **8**
Figure S26: ^{13}C NMR Spectrum of compound **8**
Figure S27: Mass Spectrum of compound **8**
Figure S28: ^{1}H NMR Spectrum of compound **9**
Figure S29: ^{13}C NMR Spectrum of compound **9**
Figure S30: Mass Spectrum of compound **9**
Figure S31: ^{1}H NMR Spectrum of compound **10**
Figure S32: ^{13}C NMR Spectrum of compound **10**
Figure S33: Mass Spectrum of compound **10**
Figure S34: ^{1}H NMR Spectrum of compound **11**
Figure S35: ^{13}C NMR Spectrum of compound **11**
Figure S36: Mass Spectrum of compound **11**
Figure S37: ^{1}H NMR Spectrum of compound **12**
Figure S38: ^{13}C NMR Spectrum of compound **12**
Figure S39: Mass Spectrum of compound **12**

Figure S1. ^{1}H NMR Spectrum of intermediate **A**

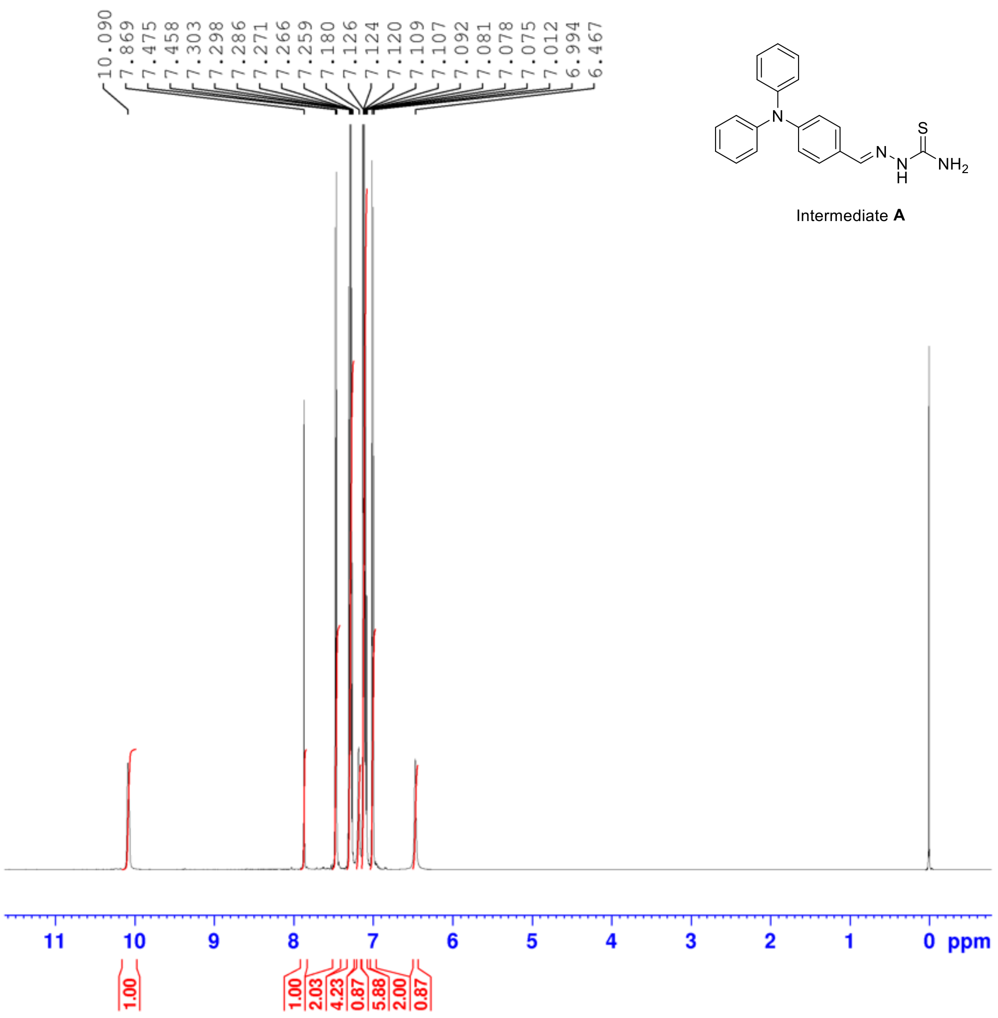

Figure S2. ^{13}C NMR Spectrum of **A**

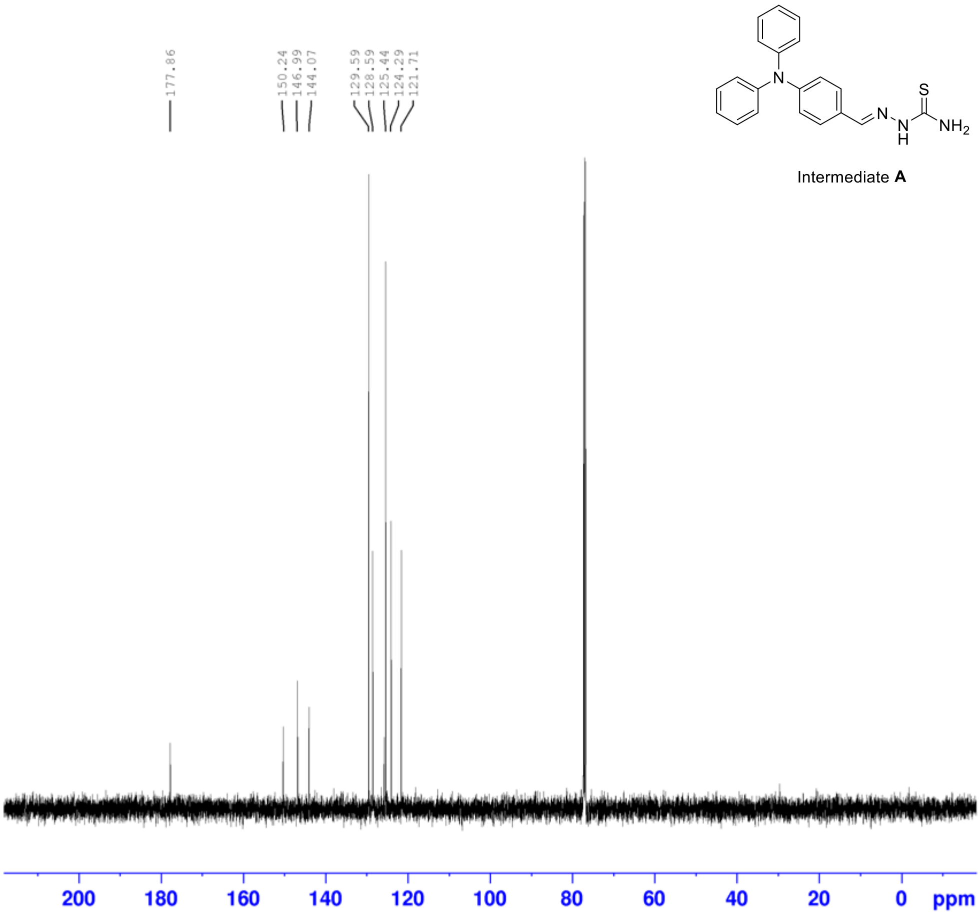

Figure S3: Mass Spectrum of intermediate **A**

```
Note : NBA
Inlet : Direct                                  Ion Mode : FAB+
RT : 1.13 min                                   Scan#: (3,8)
Elements : C 200/0, H 200/0, N 5/3, S 2/0
Mass Tolerance        : 20ppm, 10mmu if m/z < 500, 20mmu if m/z > 1000
Unsaturation (U.S.) : -0.5 - 100.0
```

Observed m/z	Int%	Err[ppm / mmu]	U.S.	Composition
346.1245	54.7	-28.6 / -9.9	18.5	C 24 H 16 N 3
		+7.7 / +2.7	19.0	C 23 H 14 N 4
		-2.0 / -0.7	15.0	C 20 H 18 N 4 S
		-11.8 / -4.1	11.0	C 17 H 22 N 4 S 2
		+24.5 / +8.5	11.5	C 16 H 20 N 5 S 2
347.1323	100.0	-28.7 / -10.0	18.0	C 24 H 17 N 3
		+7.6 / +2.6	18.5	C 23 H 15 N 4
		-2.2 / -0.7	14.5	C 20 H 19 N 4 S
		-11.9 / -4.1	10.5	C 17 H 23 N 4 S 2
		+24.4 / +8.5	11.0	C 16 H 21 N 5 S 2

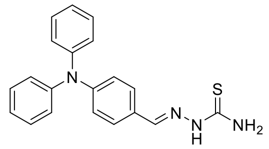

Intermediate **A**

```
[ Theoretical Ion Distribution ]                                    Page: 1
Molecular Formula : C20 H19 N4 S
                      (m/z   347.1330, MW   347.4637, U.S. 14.5)
Base Peak :    347.1330, Averaged MW :    347.4636(a),    347.4647(w)
```

m/z	INT.	
347.1330	100.0000	**
348.1359	24.7884	**************
349.1328	7.3734	****
350.1335	1.2860	*
351.1347	0.1551	
352.1358	0.0145	
353.1372	0.0011	

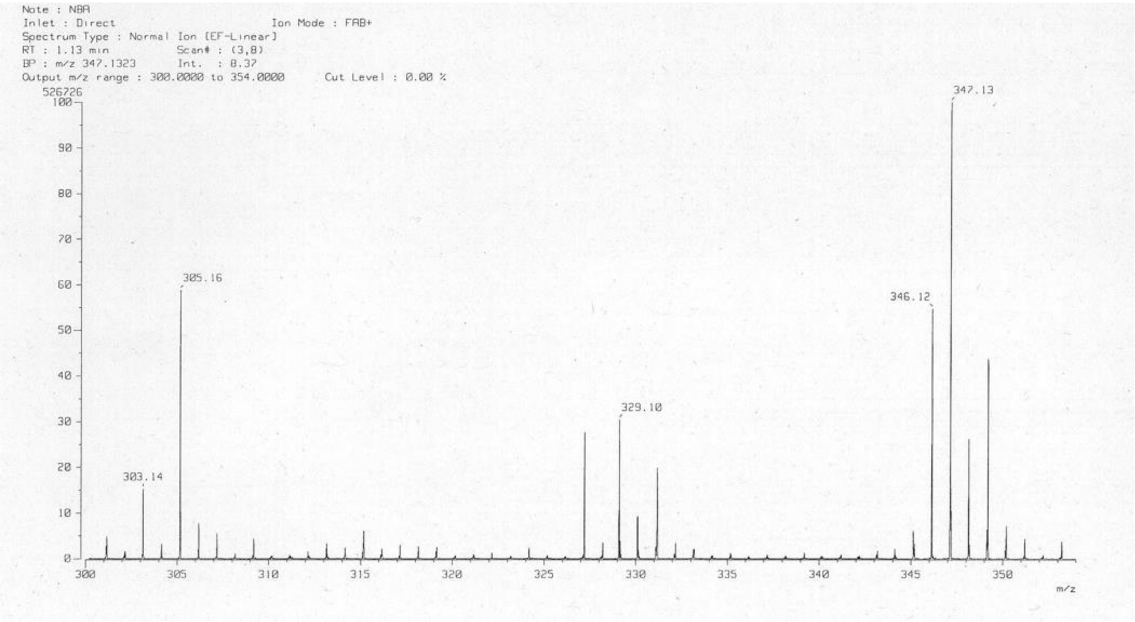

Figure S4: ^{1}H NMR Spectrum of compound **1**

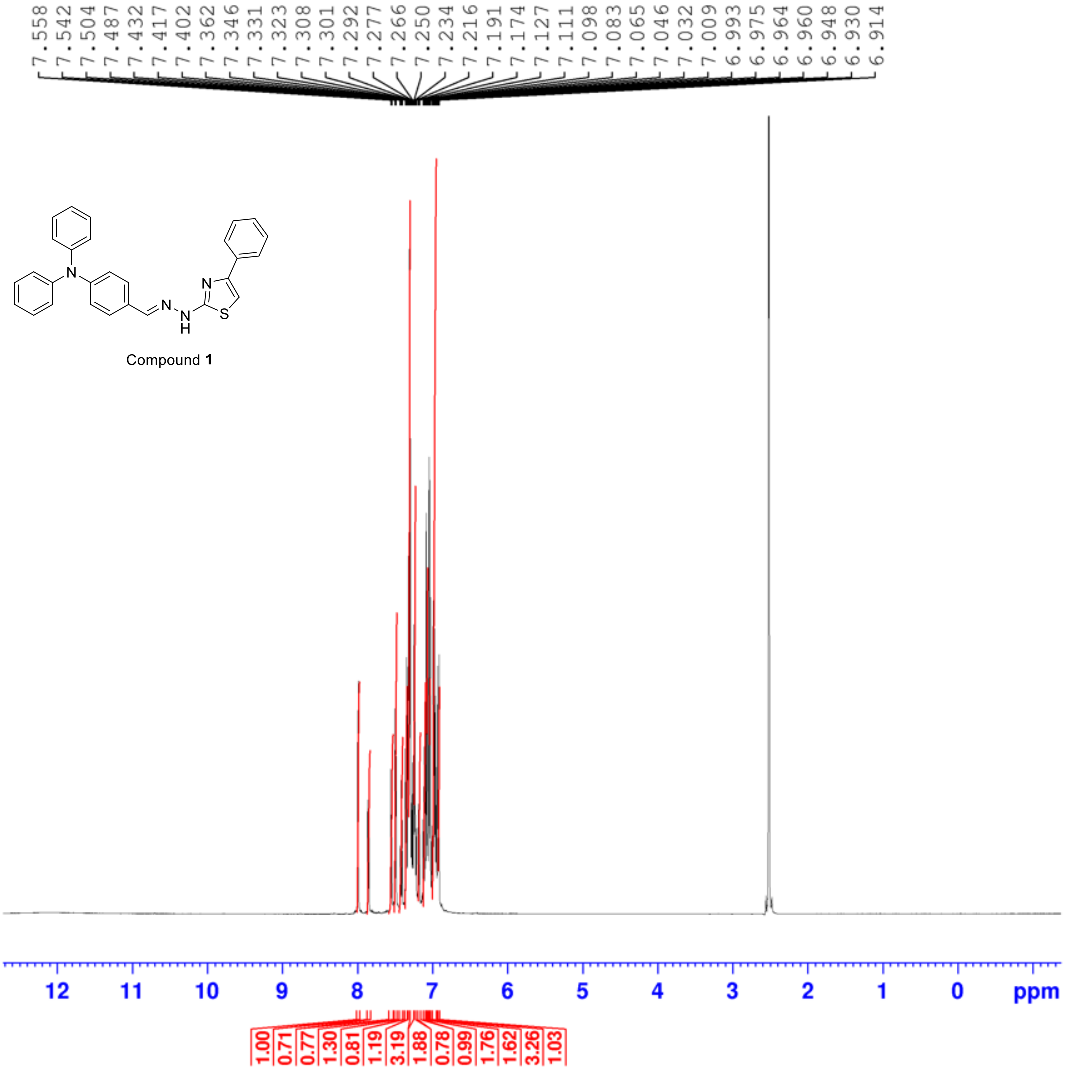

Figure S5: ^{13}C NMR Spectrum of compound **1**

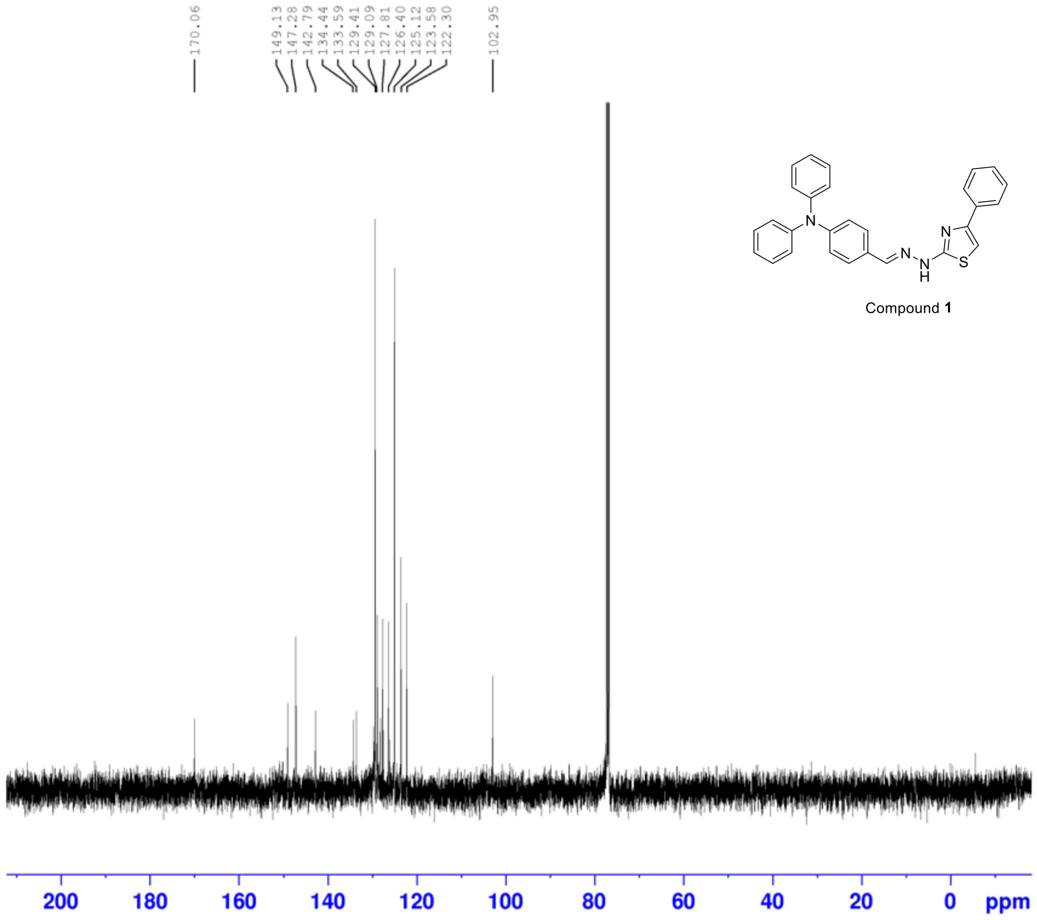

Figure S6: Mass Spectrum of compound **1**

Note : CHCl3+NBA
Inlet : Direct   Ion Mode : FAB+
RT : 0.88 min   Scan#: (3,6)
Elements : C 200/0, H 200/0, N 5/3, S 2/0
Mass Tolerance   : 20ppm, 10mmu if m/z < 500, 20mmu if m/z > 1000
Unsaturation (U.S.) : -0.5 - 100.0

Observed m/z	Int%	Err[ppm / mmu]	U.S.	Composition
446.1562	92.0	-21.4 / -9.5	24.5	C 32 H 20 N 3
		+6.8 / +3.0	25.0	C 31 H 18 N 4
		-0.7 / -0.3	21.0	C 28 H 22 N 4 S
		-8.3 / -3.7	17.0	C 25 H 26 N 4 S 2
		+19.9 / +8.9	17.5	C 24 H 24 N 5 S 2
447.1629	100.0	+4.3 / +1.9	24.5	C 31 H 19 N 4
		-3.3 / -1.5	20.5	C 28 H 23 N 4 S
		-10.8 / -4.8	16.5	C 25 H 27 N 4 S 2
		+17.3 / +7.7	17.0	C 24 H 25 N 5 S 2

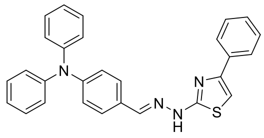

Compound **1**

[Theoretical Ion Distribution]   Page: 1
Molecular Formula : C28 H23 N4 S
                    (m/z   447.1643, MW   447.5834, U.S. 20.5)
Base Peak :   447.1643, Averaged MW :   447.5838(a),   447.5849(w)

m/z	INT.	
447.1643	100.0000	**
448.1674	33.7463	********************
449.1658	9.9457	******
450.1661	2.0416	*
451.1673	0.2983	
452.1688	0.0335	
453.1703	0.0030	
454.1722	0.0002	

Note : CHCl3+NBA
Inlet : Direct   Ion Mode : FAB+
Spectrum Type : Normal Ion [EF-Linear]
RT : 0.88 min   Scan# : (3,6)
BP : m/z 447.1629   Int. : 4.06
Output m/z range : 432.0000 to 486.0000   Cut Level : 0.00 %

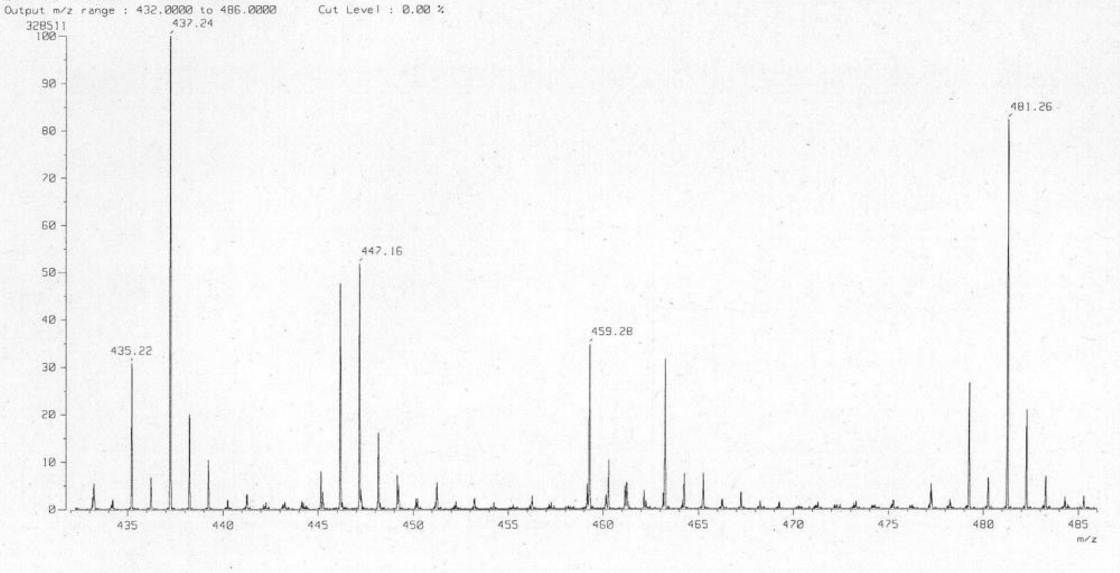

Figure S7: ^{1}H NMR Spectrum of compound **2**

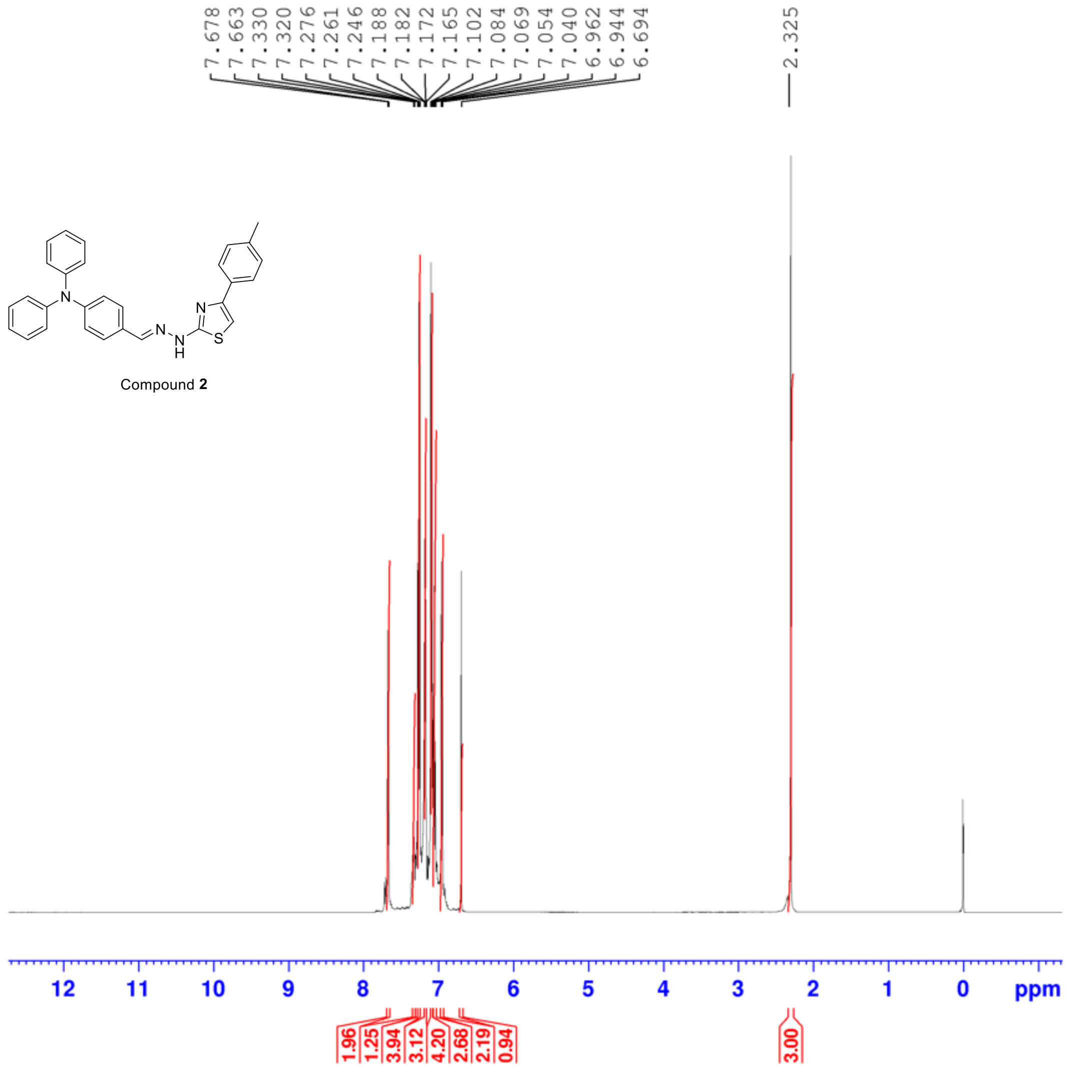

Figure S8: ^{13}C NMR Spectrum of compound **2**

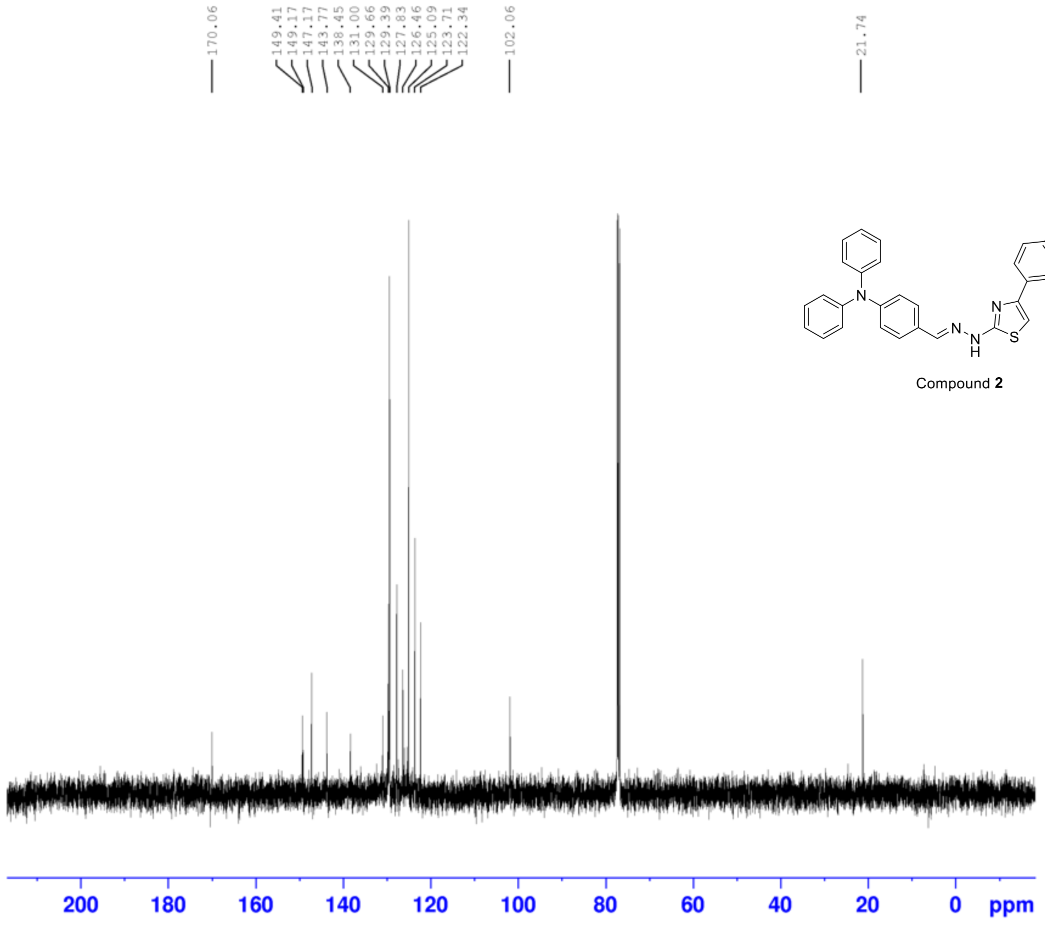

Figure S9: Mass Spectrum of compound **2**

Note : NBA
Inlet : Direct   Ion Mode : FAB+
RT : 0.88 min   Scan#: (3,6)
Elements : C 200/0, H 200/0, N 5/3, S 2/0
Mass Tolerance : 20ppm, 10mmu if m/z < 500, 20mmu if m/z > 1000
Unsaturation (U.S.) : -0.5 - 100.0

Observed m/z	Int%	Err[ppm / mmu]	U.S.	Composition
461.1787	100.0	+4.5 / +2.1	24.5	C 32 H 21 N 4
		-2.8 / -1.3	20.5	C 29 H 25 N 4 S
		-10.2 / -4.7	16.5	C 26 H 29 N 4 S 2
		+17.1 / +7.9	17.0	C 25 H 27 N 5 S 2
460.1724	89.6	-19.5 / -9.0	24.5	C 33 H 22 N 3
		+7.8 / +3.6	25.0	C 32 H 20 N 4
		+0.5 / +0.2	21.0	C 29 H 24 N 4 S
		-6.8 / -3.1	17.0	C 26 H 28 N 4 S 2
		+20.5 / +9.4	17.5	C 25 H 26 N 5 S 2

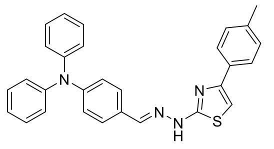

Compound **2**

[Theoretical Ion Distribution]   Page: 1
Molecular Formula : C29 H25 N4 S
(m/z 461.1800, MW 461.6103, U.S. 20.5)
Base Peak : 461.1800, Averaged MW : 461.6108(a), 461.6118(w)

m/z	INT.	
461.1800	100.0000	**
462.1830	34.8885	********************
463.1816	10.3315	******
464.1819	2.1553	*
465.1831	0.3217	
466.1846	0.0369	
467.1862	0.0034	
468.1881	0.0003	

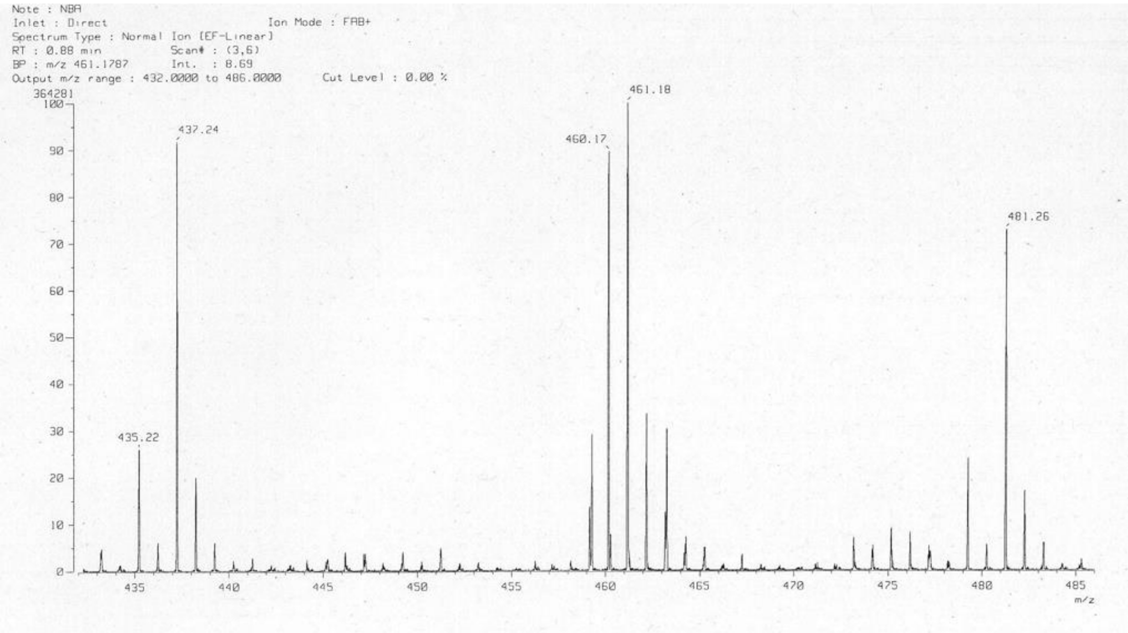

Figure S10: ^{1}H NMR Spectrum of compound **3**

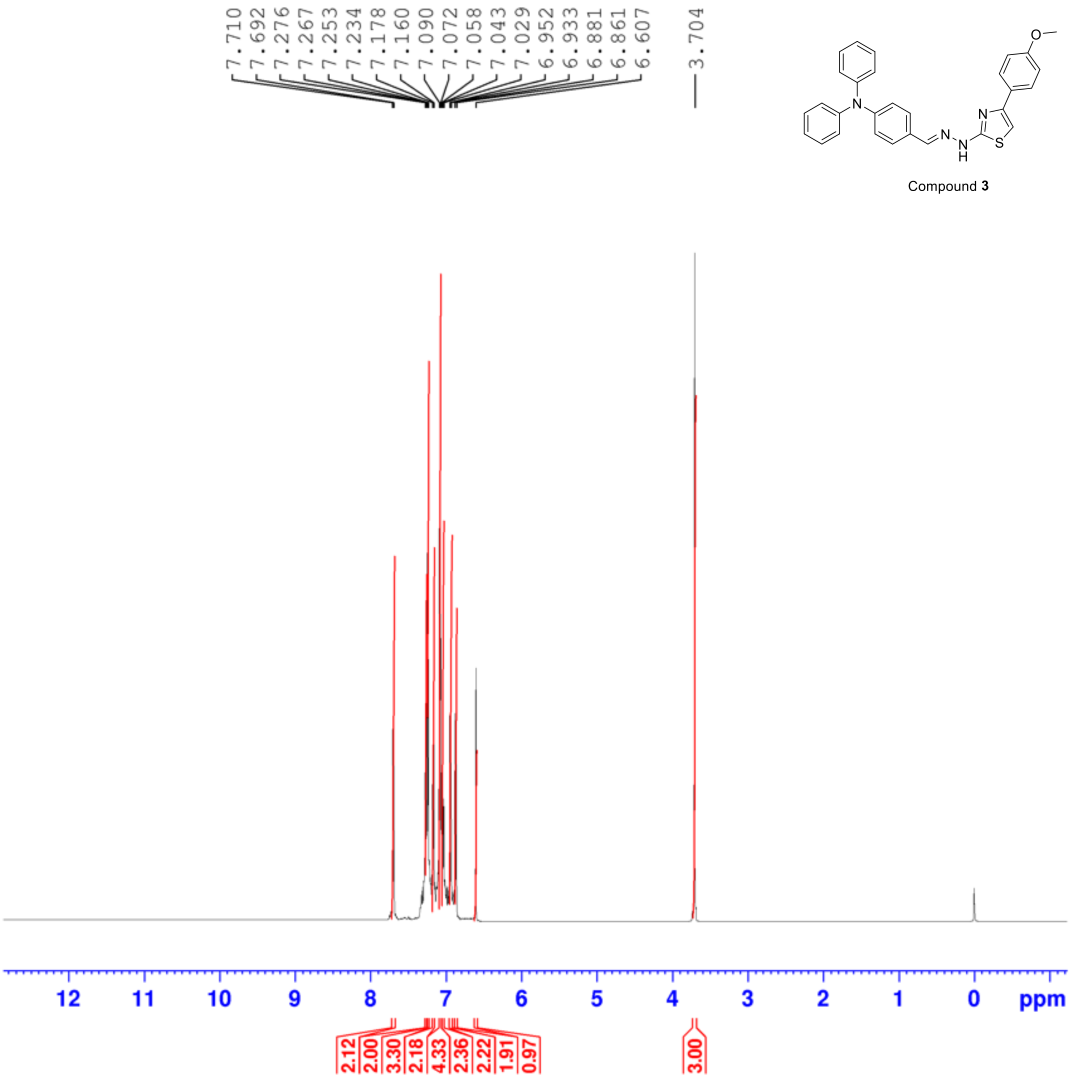

Figure S11: ^{13}C NMR Spectrum of compound **3**

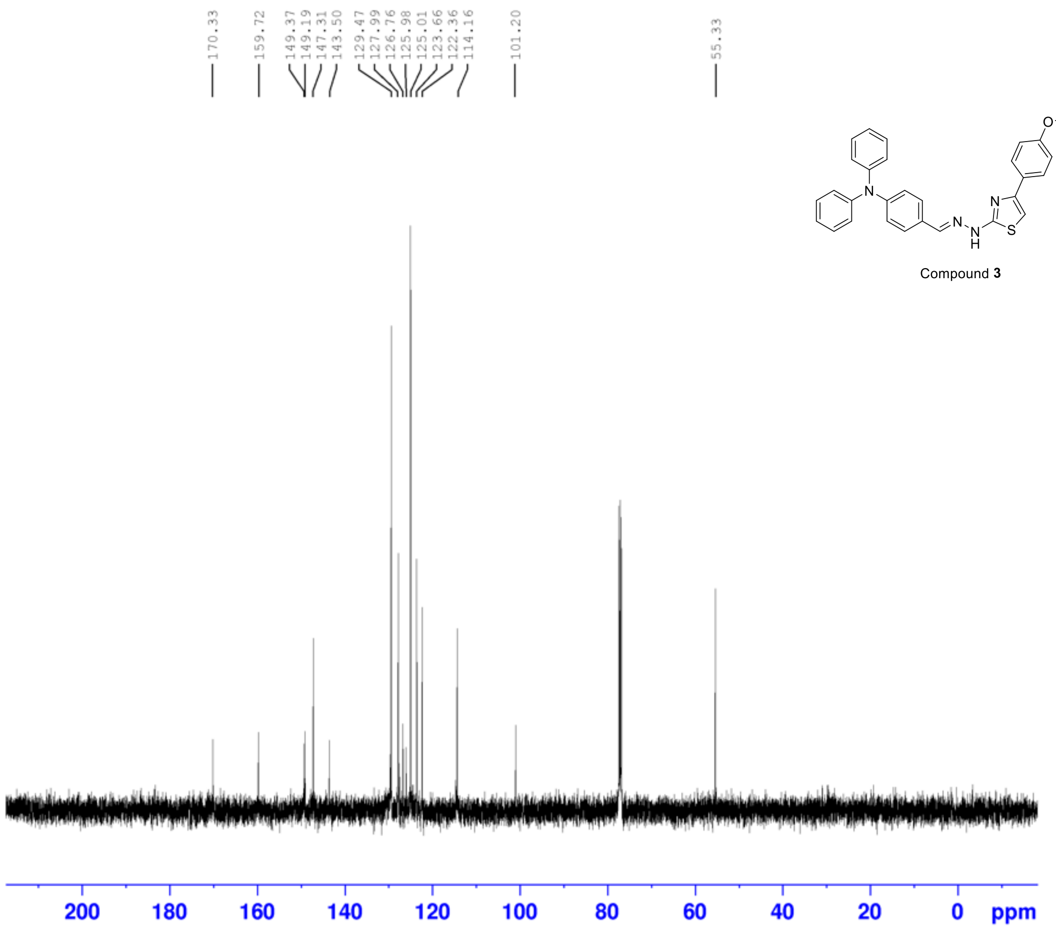

Figure S12: Mass Spectrum of compound **3**

Note : NBA
Inlet : Direct Ion Mode : FAB+
RT : 0.88 min Scan#: (3,6)
Elements : C 200/0, H 200/0, O 2/0, N 5/3, S 2/0
Mass Tolerance : 20ppm, 10mmu if m/z < 500, 20mmu if m/z > 1000
Unsaturation (U.S.) : -0.5 - 100.0

Observed m/z	Int%	Err[ppm / mmu]	U.S.	Composition
476.1678	82.0	-17.9 / -8.5	24.5	C 33 H 22 O N 3
		+8.5 / +4.0	25.0	C 32 H 20 O N 4
		+1.4 / +0.7	21.0	C 29 H 24 O N 4 S
		-5.7 / -2.7	17.0	C 26 H 28 O N 4 S 2
		+20.7 / +9.9	17.5	C 25 H 26 O N 5 S 2
477.1743	100.0	-20.5 / -9.8	24.0	C 33 H 23 O N 3
		+5.9 / +2.8	24.5	C 32 H 21 O N 4
		-1.2 / -0.6	20.5	C 29 H 25 O N 4 S
		-8.2 / -3.9	16.5	C 26 H 29 O N 4 S 2
		+18.1 / +8.6	17.0	C 25 H 27 O N 5 S 2

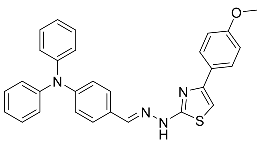

Compound **3**

[Theoretical Ion Distribution] Page: 1
Molecular Formula : C29 H25 O N4 S
(m/z 477.1749, MW 477.6097, U.S. 20.5)
Base Peak : 477.1749, Averaged MW : 477.6101(a), 477.6111(w)

m/z	INT.	
477.1749	100.0000	**
478.1779	34.9266	********************
479.1766	10.5452	******
480.1770	2.2292	*
481.1782	0.3432	
482.1797	0.0413	
483.1813	0.0041	
484.1832	0.0003	

Note : NBA
Inlet : Direct Ion Mode : FAB+
Spectrum Type : Normal Ion [EF-Linear]
RT : 0.88 min Scan# : (3,6)
BP : m/z 477.1743 Int. : 11.61
Output m/z range : 432.0000 to 486.0000 Cut Level : 0.00 %

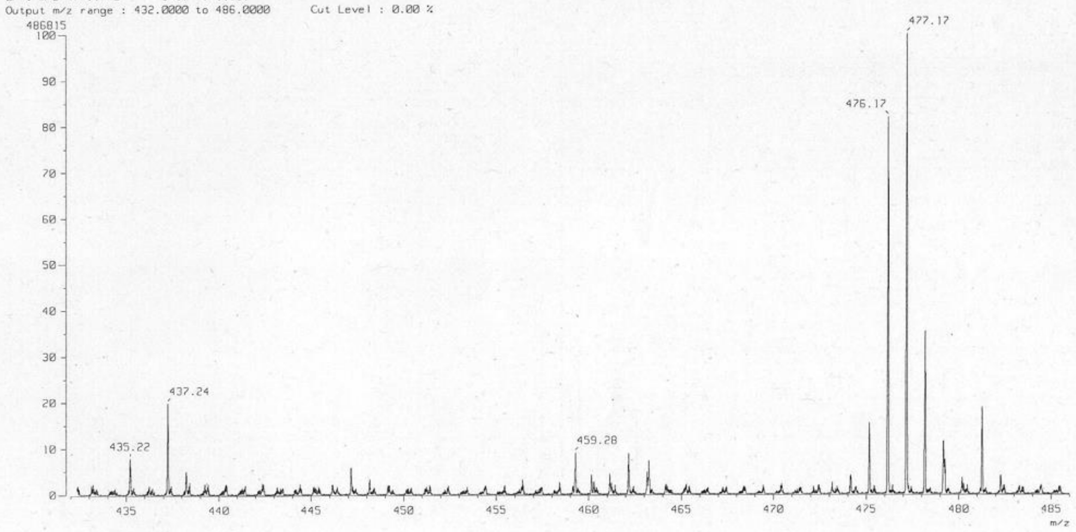

Figure S13: ^{1}H NMR Spectrum of compound **4**

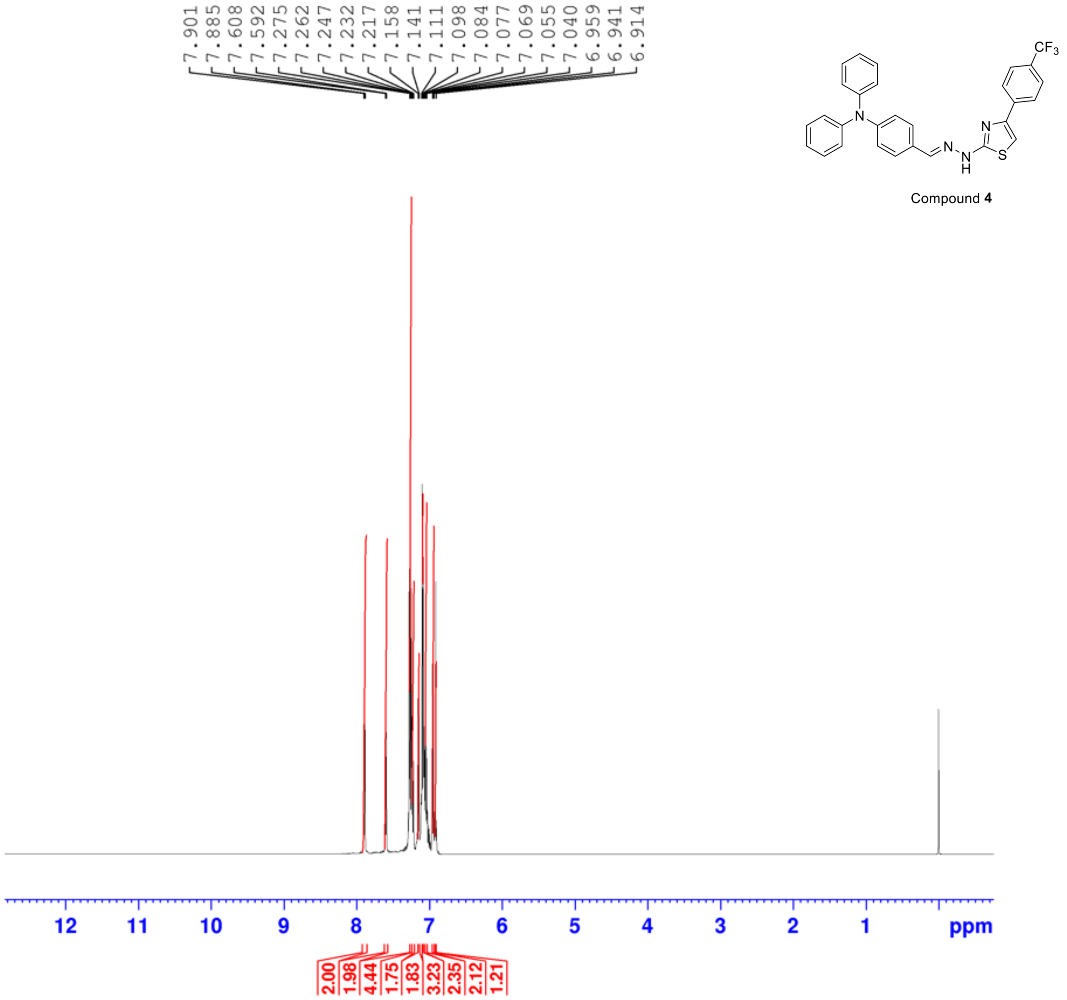

Compound **4**

Figure S14: ^{13}C NMR Spectrum of compound **4**

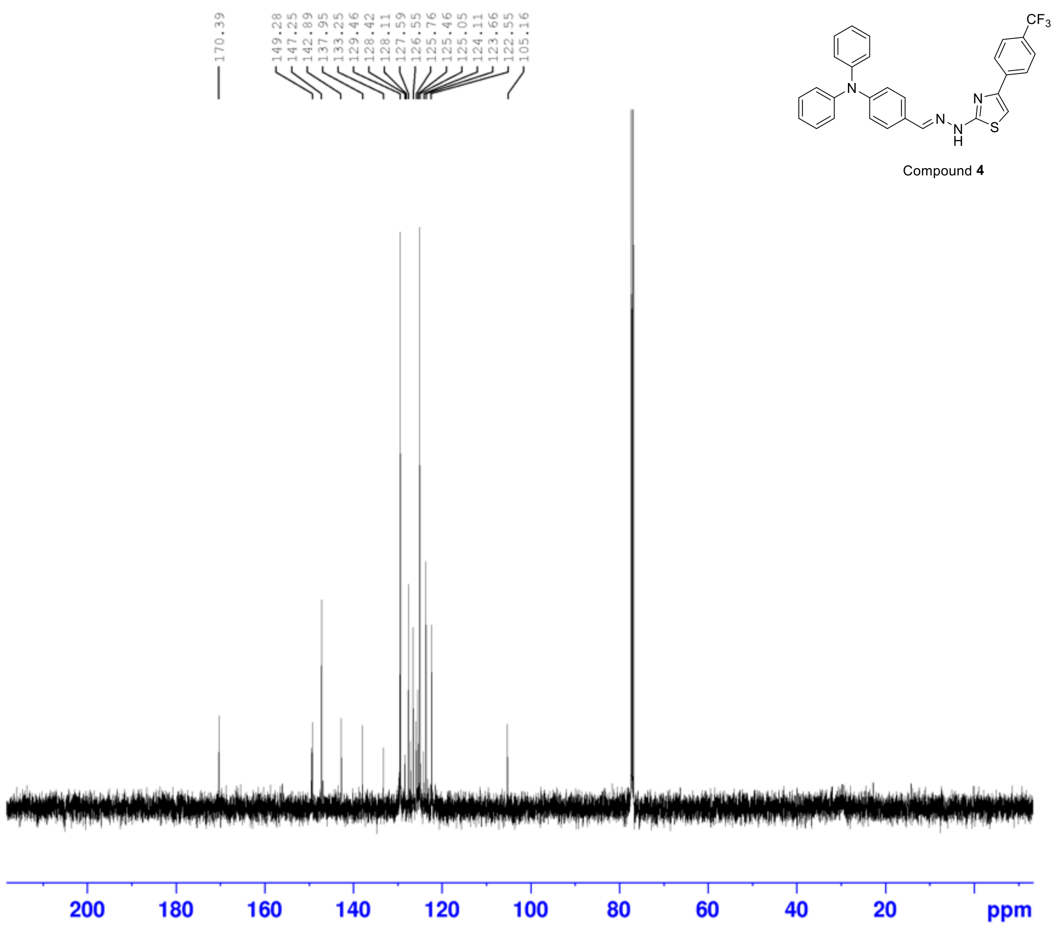

Figure S15: Mass Spectrum of compound **4**

```
Note : NBA
Inlet : Direct                          Ion Mode : FAB+
RT : 1.25 min                           Scan#: (4,8)
Elements : C 200/0, H 200/0, N 5/3, F 4/2, S 2/0
Mass Tolerance      : 20ppm, 10mmu if m/z < 500, 20mmu if m/z > 1000
Unsaturation (U.S.) : -0.5 - 100.0

Observed m/z Int%   Err[ppm / mmu]    U.S.  Composition
  514.1442   100.0    -17.3 /  -8.9   24.5  C 33 H 19 N 3 F 3
                       +7.2 /  +3.7   25.0  C 32 H 17 N 4 F 3
                       +0.6 /  +0.3   21.0  C 29 H 21 N 4 F 3 S
                       -5.9 /  -3.0   17.0  C 26 H 25 N 4 F 3 S 2
                      +18.5 /  +9.5   17.5  C 25 H 23 N 5 F 3 S 2

  515.1500    81.3     +3.1 /  +1.6   24.5  C 32 H 18 N 4 F 3
                       -3.4 /  -1.8   20.5  C 29 H 22 N 4 F 3 S
                      -10.0 /  -5:1   16.5  C 26 H 26 N 4 F 3 S 2
                      +14.4 /  +7.4   17.0  C 25 H 24 N 5 F 3 S 2
```

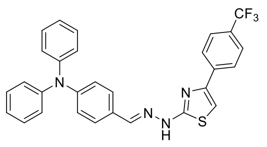

Compound **4**

```
[ Theoretical Ion Distribution ]                              Page: 1
Molecular Formula : C29 H21 N4 F3 S
                    (m/z   514.1439, MW   514.5737, U.S. 21.0)
Base Peak :   514.1439, Averaged MW :   514.5741(a),   514.5750(w)

   m/z        INT.
 514.1439 100.0000 *************************************************************
 515.1469  34.8285 *********************
 516.1455  10.3106 ******
 517.1458   2.1491 *
 518.1470   0.3204
 519.1485   0.0367
 520.1501   0.0034
 521.1519   0.0003
```

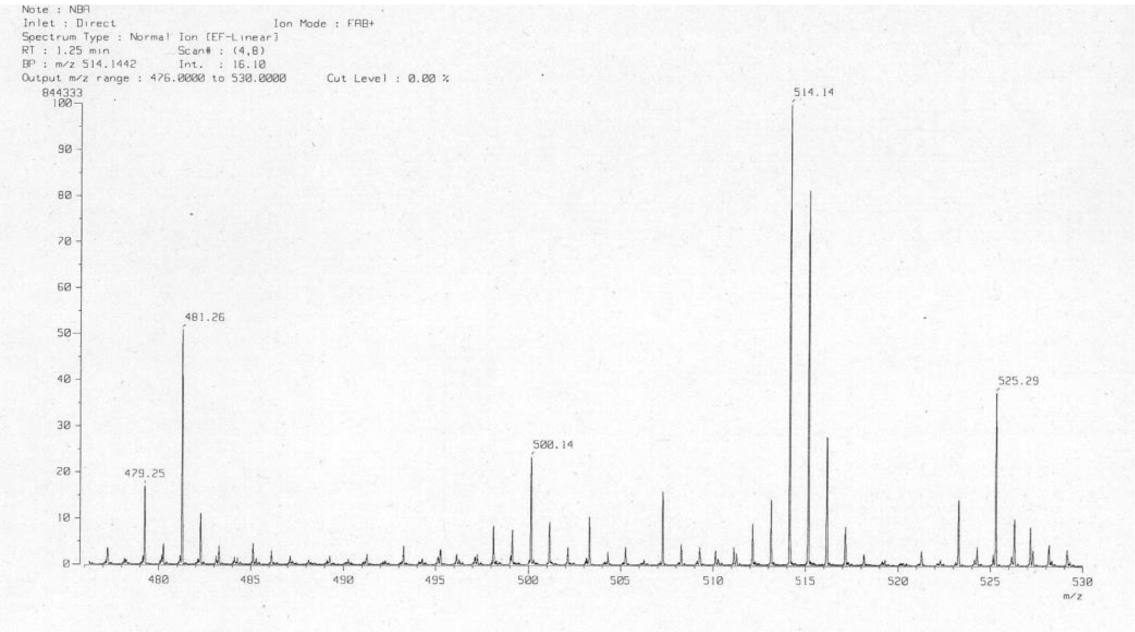

Figure S16: ^{1}H NMR Spectrum of compound **5**

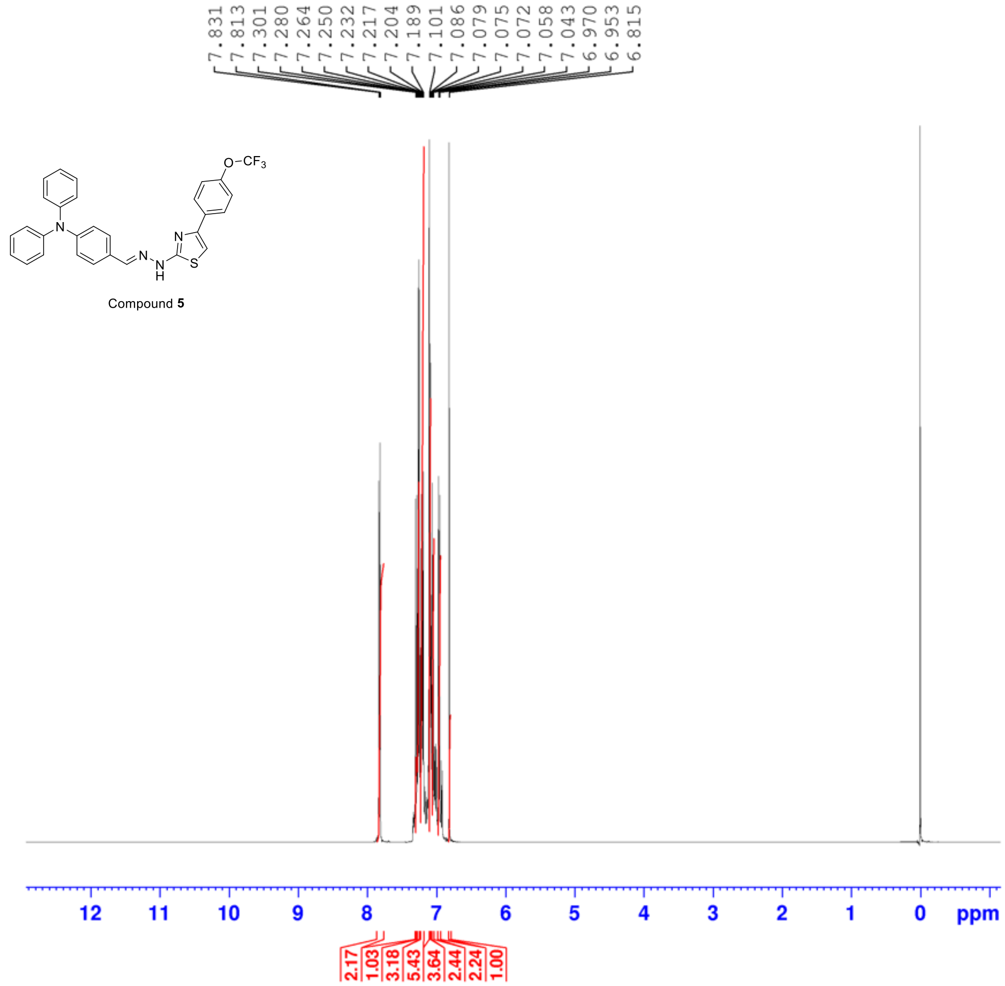

Figure S17: ^{13}C NMR Spectrum of compound **5**

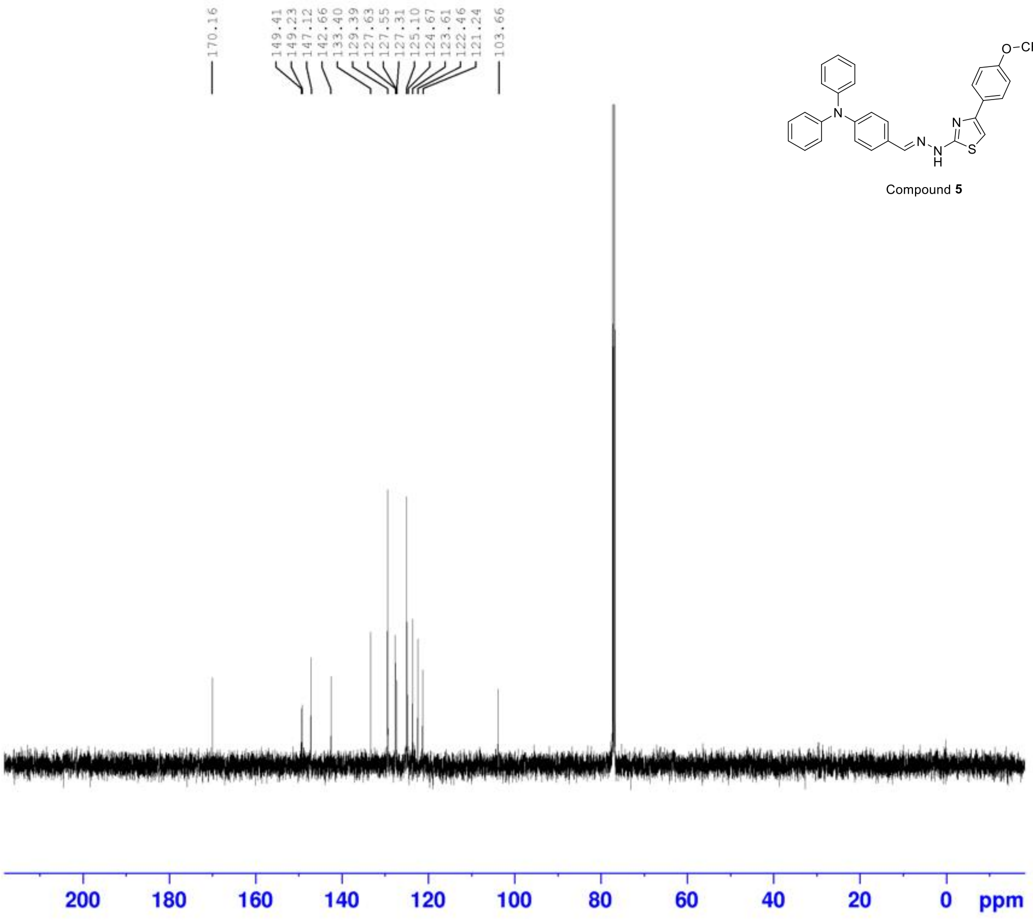

Figure S18: Mass Spectrum of compound **5**

Note : NBA
Inlet : Direct   Ion Mode : FAB+
RT : 1.38 min   Scan#: (4,9)
Elements : C 200/0, H 200/0, O 2/0, N 5/3, F 4/2, S 2/0
Mass Tolerance : 20ppm, 10mmu if m/z < 500, 20mmu if m/z > 1000
Unsaturation (U.S.) : -0.5 - 100.0

Observed m/z	Int%	Err[ppm / mmu]	U.S.	Composition
530.1390	100.0	-14.8 / -7.8	28.5	C 36 H 18 N 3 F 2
		+8.9 / +4.7	29.0	C 35 H 16 N 4 F 2
		-7.2 / -3.8	24.5	C 31 H 18 O 2 N 5 F 2
		-16.9 / -9.0	24.5	C 33 H 19 O N 3 F 3
		+6.8 / +3.6	25.0	C 32 H 17 O N 4 F 3
		-19.1 / -10.1	20.5	C 30 H 20 O 2 N 3 F 4
		+4.6 / +2.4	21.0	C 29 H 18 O 2 N 4 F 4
		+2.6 / +1.4	25.0	C 32 H 20 N 4 F 2 S
		-13.6 / -7.2	20.5	C 28 H 22 O 2 N 5 F 2 S
		+0.4 / +0.2	21.0	C 29 H 21 O N 4 F 3 S
		+14.4 / +7.6	21.5	C 30 H 20 N 3 F 4 S
		-1.7 / -0.9	17.0	C 26 H 22 O 2 N 4 F 4 S
		-3.8 / -2.0	21.0	C 29 H 24 N 4 F 2 S 2
		+19.9 / +10.6	21.5	C 28 H 22 N 5 F 2 S 2
		-19.9 / -10.6	16.5	C 25 H 26 O 2 N 5 F 2 S 2
		-5.9 / -3.2	17.0	C 26 H 25 O N 4 F 3 S 2
		+17.8 / +9.4	17.5	C 25 H 23 O N 5 F 3 S 2
		+8.0 / +4.3	17.5	C 27 H 24 N 3 F 4 S 2
		-8.1 / -4.3	13.0	C 23 H 26 O 2 N 4 F 4 S 2
		+15.6 / +8.3	13.5	C 22 H 24 O 2 N 5 F 4 S 2
531.1450	90.5	-18.2 / -9.7	28.0	C 36 H 19 N 3 F 2
		+5.4 / +2.9	28.5	C 35 H 17 N 4 F 2
		-10.7 / -5.7	24.0	C 31 H 19 O 2 N 5 F 2
		+3.3 / +1.7	24.5	C 32 H 18 O N 4 F 3
		+17.2 / +9.2	25.0	C 33 H 17 N 3 F 4
		+1.1 / +0.6	20.5	C 29 H 19 O 2 N 4 F 4
		-0.9 / -0.5	24.5	C 32 H 21 N 4 F 2 S
		-17.0 / -9.0	20.0	C 28 H 23 O 2 N 5 F 2 S
		-3.1 / -1.6	20.5	C 29 H 22 O N 4 F 3 S
		+10.9 / +5.8	21.0	C 30 H 21 N 3 F 4 S
		-5.2 / -2.8	16.5	C 26 H 23 O 2 N 4 F 4 S
		+18.5 / +9.8	17.0	C 25 H 21 O 2 N 5 F 4 S
		-7.3 / -3.9	20.5	C 29 H 25 N 4 F 2 S 2
		+16.4 / +8.7	21.0	C 28 H 23 N 5 F 2 S 2
		-9.4 / -5.0	16.5	C 26 H 26 O N 4 F 3 S 2
		+14.3 / +7.6	17.0	C 25 H 24 O N 5 F 3 S 2
		+4.5 / +2.4	17.0	C 27 H 25 N 3 F 4 S 2
		-11.6 / -6.1	12.5	C 23 H 27 O 2 N 4 F 4 S 2
		+12.1 / +6.4	13.0	C 22 H 25 O 2 N 5 F 4 S 2

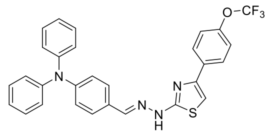

Compound **5**

[Theoretical Ion Distribution]   Page: 1
Molecular Formula : C29 H21 O N4 F3 S
(m/z 530.1388, MW 530.5731, U.S. 21.0)
Base Peak : 530.1388, Averaged MW : 530.5734(a), 530.5743(w)

m/z	INT.	
530.1388	100.0000	**
531.1418	34.8666	********************
532.1405	10.5243	******
533.1409	2.2229	*
534.1421	0.3419	
535.1436	0.0411	
536.1452	0.0040	
537.1470	0.0003	

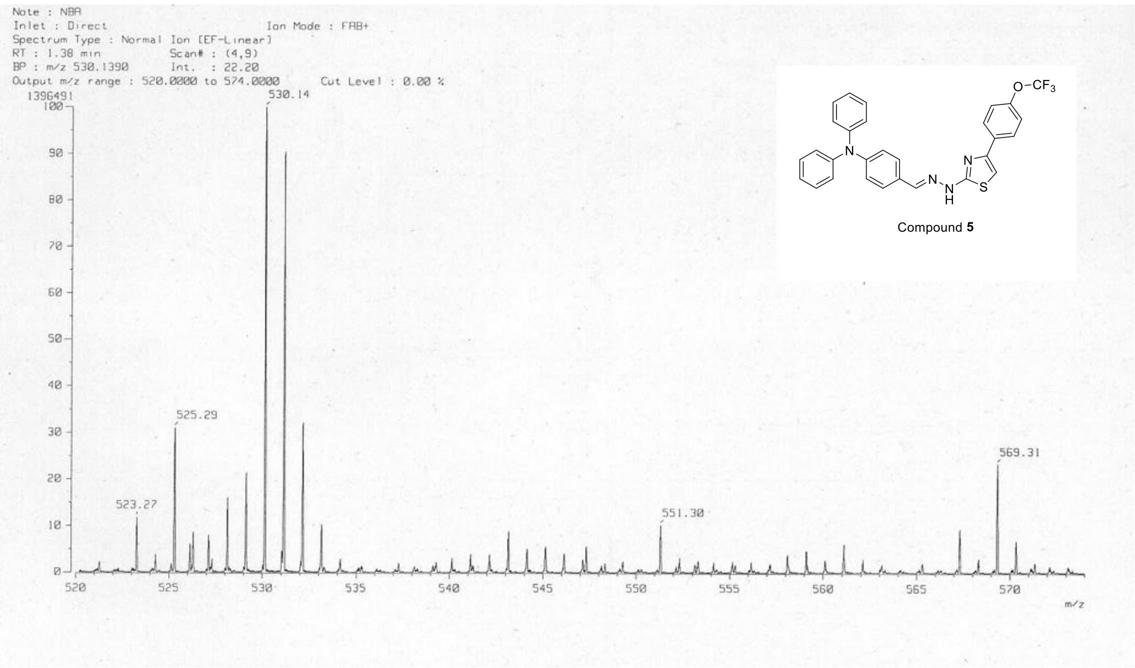
Note : NBA
Inlet : Direct
Ion Mode : FAB+
Spectrum Type : Normal Ion [EF-Linear]
RT : 1.38 min
Scan# : (4,9)
BP : m/z 530.1390
Int. : 22.20
Output m/z range : 520.0000 to 574.0000
Cut Level : 0.00 %
1396491
530.14
525.29
523.27
551.30
569.31
m/z
Compound 5

Figure S19: ^{1}H NMR Spectrum of compound **6**

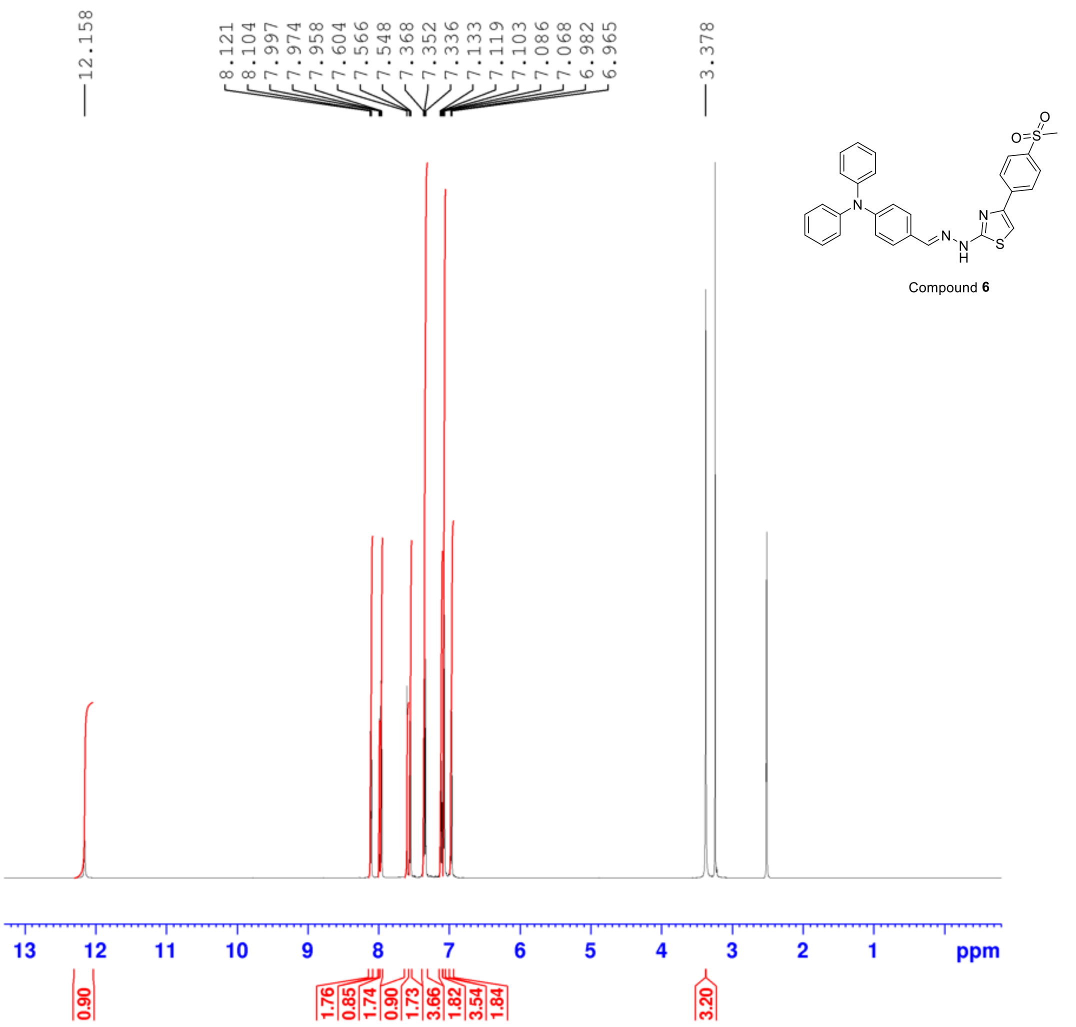

Figure S20: ^{13}C NMR Spectrum of compound **6**

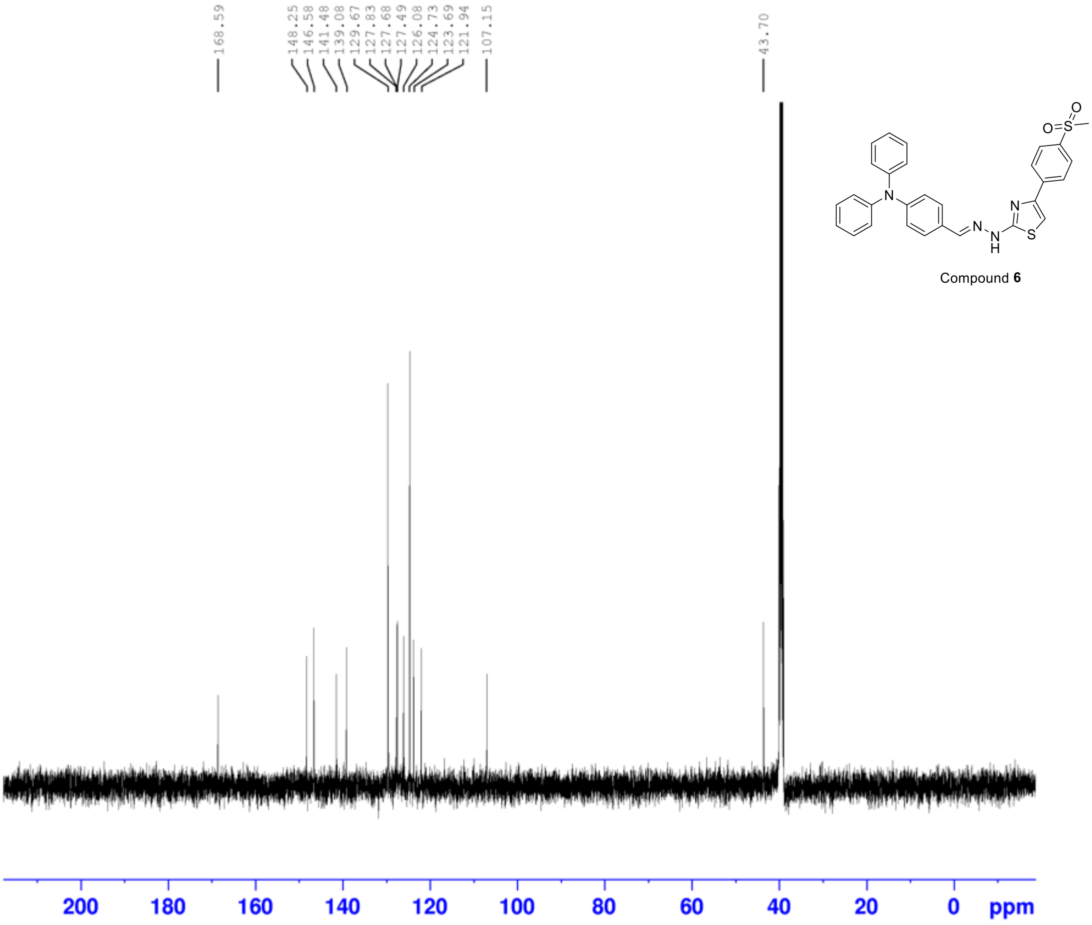

Figure S21: Mass Spectrum of compound **6**

Note : NBA
Inlet : Direct   Ion Mode : FAB+
RT : 1.88 min   Scan#: (6,11)
Elements : C 200/0, H 200/0, O 3/1, N 5/3, S 3/1
Mass Tolerance : 20ppm, 10mmu if m/z < 500, 20mmu if m/z > 1000
Unsaturation (U.S.) : -0.5 - 100.0

Observed m/z	Int%	Err[ppm / mmu]	U.S.	Composition
524.1325	94.5	+3.5 / +1.8	26.0	C 32 H 20 O 2 N 4 S
		-3.0 / -1.6	22.0	C 29 H 24 O 2 N 4 S 2
		-9.4 / -4.9	18.0	C 26 H 28 O 2 N 4 S 3
		+14.6 / +7.6	18.5	C 25 H 26 O 2 N 5 S 3
525.1397	78.2	+2.3 / +1.2	25.5	C 32 H 21 O 2 N 4 S
		-4.2 / -2.2	21.5	C 29 H 25 O 2 N 4 S 2
		+19.8 / +10.4	22.0	C 28 H 23 O 2 N 5 S 2
		-10.6 / -5.6	17.5	C 26 H 29 O 2 N 4 S 3
		+13.4 / +7.0	18.0	C 25 H 27 O 2 N 5 S 3

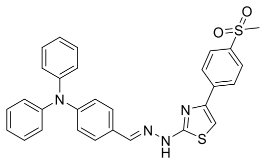

Compound **6**

[Theoretical Ion Distribution]   Page: 1
Molecular Formula : C29 H24 O2 N4 S2
(m/z 524.1341, MW 524.6672, U.S. 22.0)
Base Peak : 524.1341, Averaged MW : 524.6658(a), 524.6671(w)

m/z	INT.	
524.1341	100.0000	**
525.1370	35.7390	**********************
526.1341	15.4603	*********
527.1350	3.9350	**
528.1341	0.8805	*
529.1342	0.1581	
530.1347	0.0235	
531.1354	0.0030	
532.1363	0.0003	

Note : NBA
Inlet : Direct   Ion Mode : FAB+
Spectrum Type : Normal Ion [EF-Linear]
RT : 1.88 min   Scan# : (6,11)
BP : m/z 479.2478   Int. : 2.63
Output m/z range : 476.0000 to 530.0000   Cut Level : 0.00 %

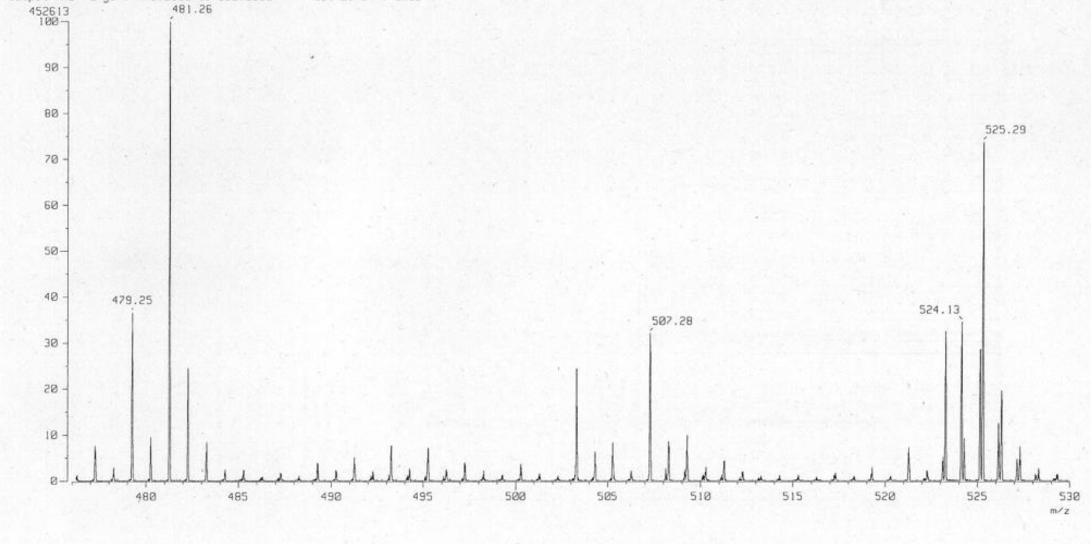

Figure S22: ^{1}H NMR Spectrum of compound **7**

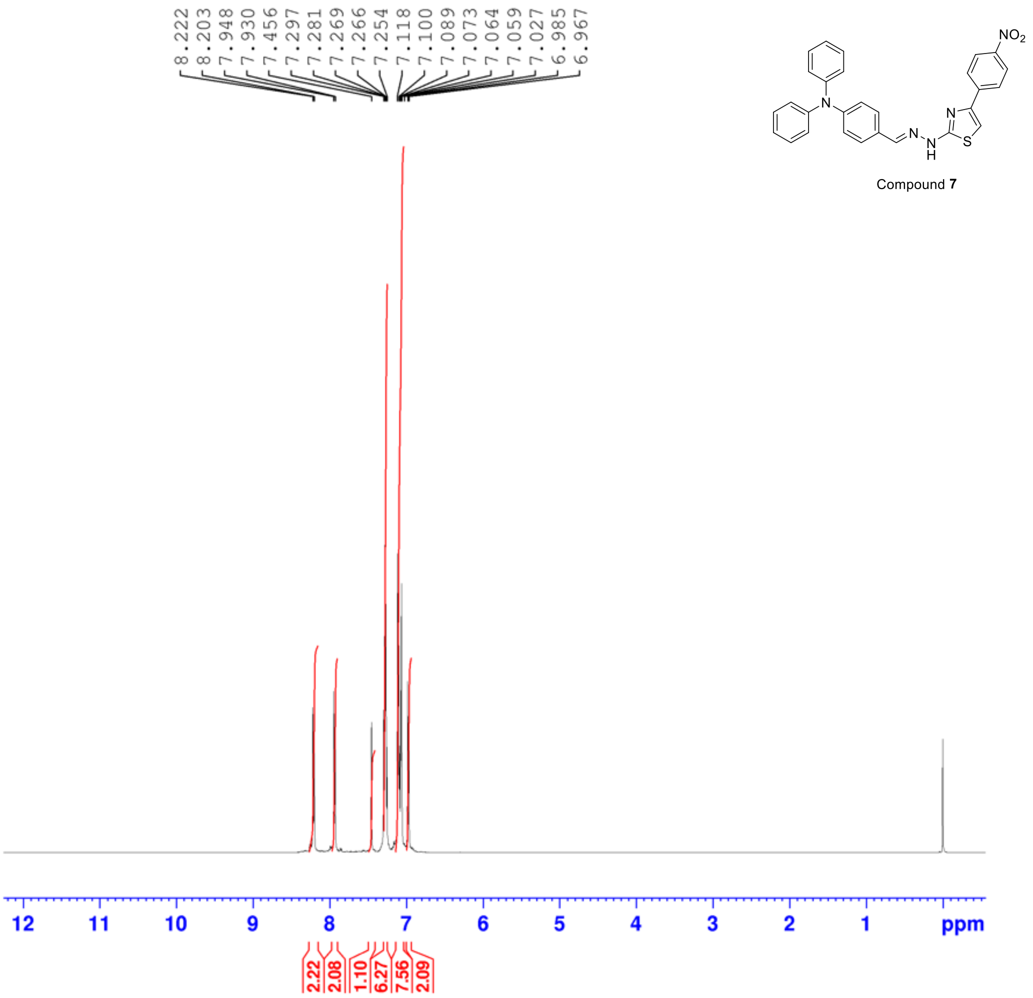

Compound **7**

Figure S23: ^{13}C NMR Spectrum of compound **7**

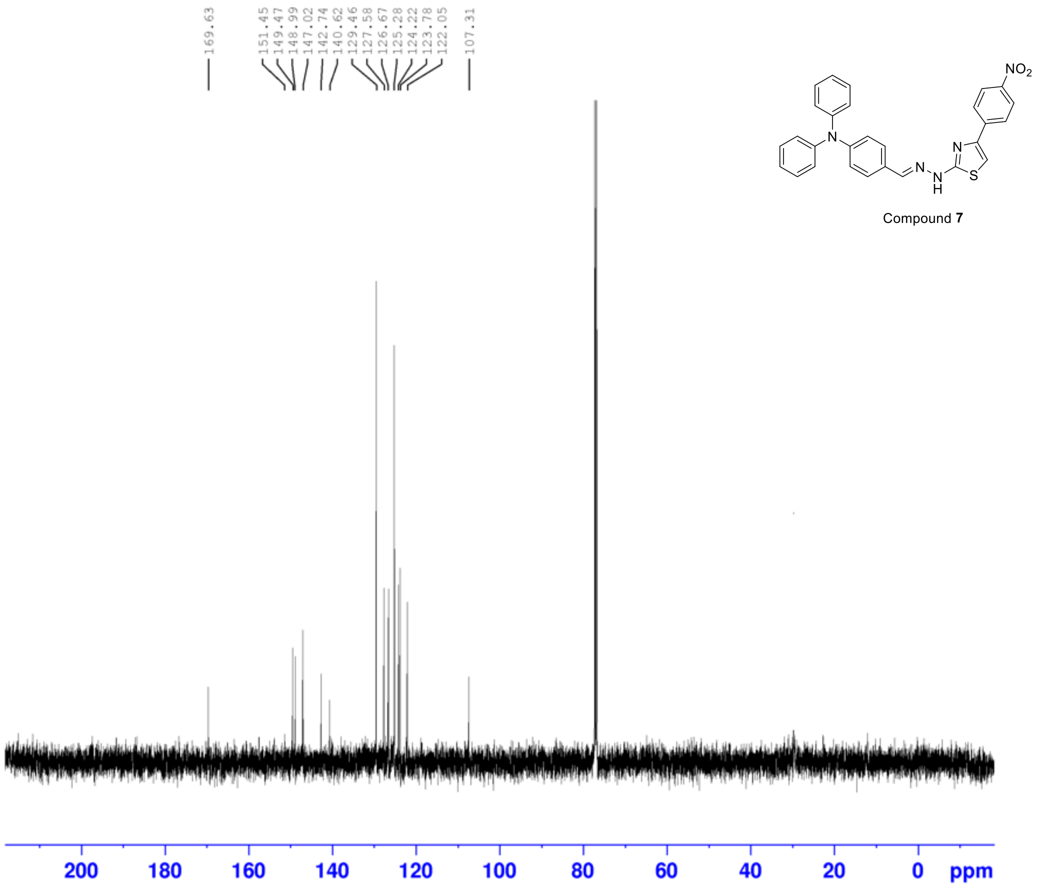

Figure S24: Mass Spectrum of compound **7**

Note : NBA
Inlet : Direct Ion Mode : FAB+
RT : 1.25 min Scan#: (4,8)
Elements : C 200/0, H 200/0, O 3/1, N 6/4, S 2/0
Mass Tolerance : 20ppm, 10mmu if m/z < 500, 20mmu if m/z > 1000
Unsaturation (U.S.) : -0.5 - 100.0

Observed m/z	Int%	Err[ppm / mmu]	U.S.	Composition
491.1395	92.0	+2.5 / +1.2	26.0	C 31 H 17 O 2 N 5
		-4.3 / -2.1	22.0	C 28 H 21 O 2 N 5 S
		-11.2 / -5.5	18.0	C 25 H 25 O 2 N 5 S 2
		+14.4 / +7.1	18.5	C 24 H 23 O 2 N 6 S 2
492.1447	74.2	-2.8 / -1.4	25.5	C 31 H 18 O 2 N 5
		-9.7 / -4.8	21.5	C 28 H 22 O 2 N 5 S
		+15.9 / +7.8	22.0	C 27 H 20 O 2 N 6 S
		-16.5 / -8.1	17.5	C 25 H 26 O 2 N 5 S 2
		+9.0 / +4.4	18.0	C 24 H 24 O 2 N 6 S 2

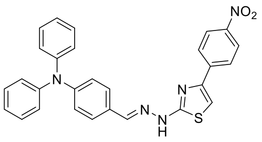

Compound **7**

[Theoretical Ion Distribution] Page: 1
Molecular Formula : C28 H21 O2 N5 S
(m/z 491.1416, MW 491.5730, U.S. 22.0)
Base Peak : 491.1416, Averaged MW : 491.5731(a), 491.5742(w)

m/z	INT.	
491.1416	100.0000	**
492.1445	34.1598	********************
493.1432	10.4864	******
494.1437	2.2196	*
495.1448	0.3476	
496.1462	0.0432	
497.1478	0.0044	
498.1496	0.0004	

Note : NBA
Inlet : Direct Ion Mode : FAB+
Spectrum Type : Normal Ion [EF-Linear]
RT : 1.25 min Scan# : (4,8)
BP : m/z 479.2472 Int. : 1.83
Output m/z range : 476.0000 to 530.0000 Cut Level : 0.00 %

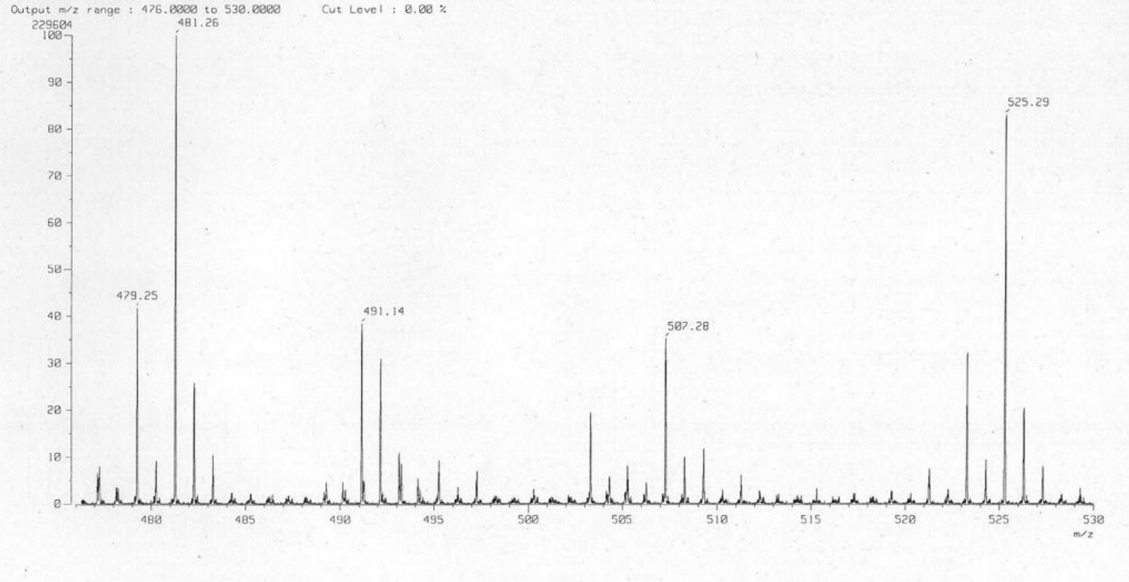

Figure S25: ^{1}H NMR Spectrum of compound **8**

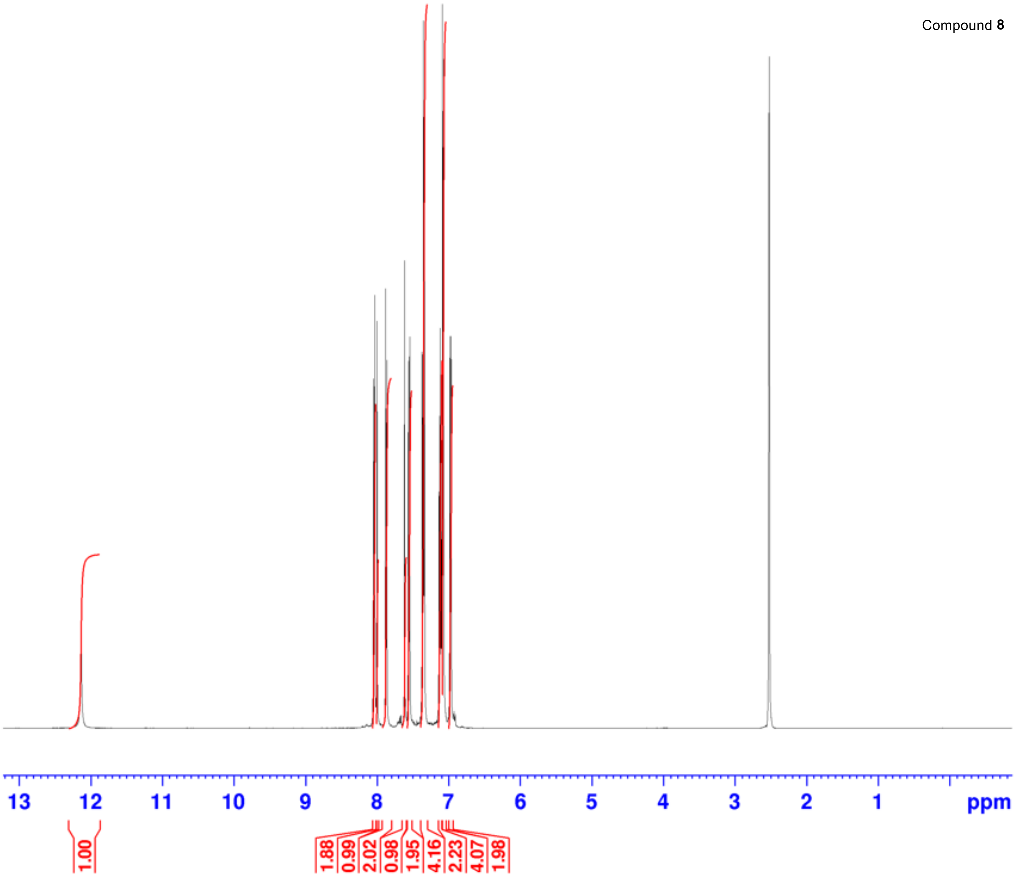

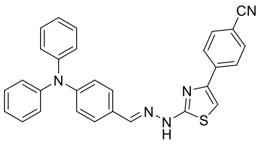

Compound **8**

Figure S26: ^{13}C NMR Spectrum of compound **8**

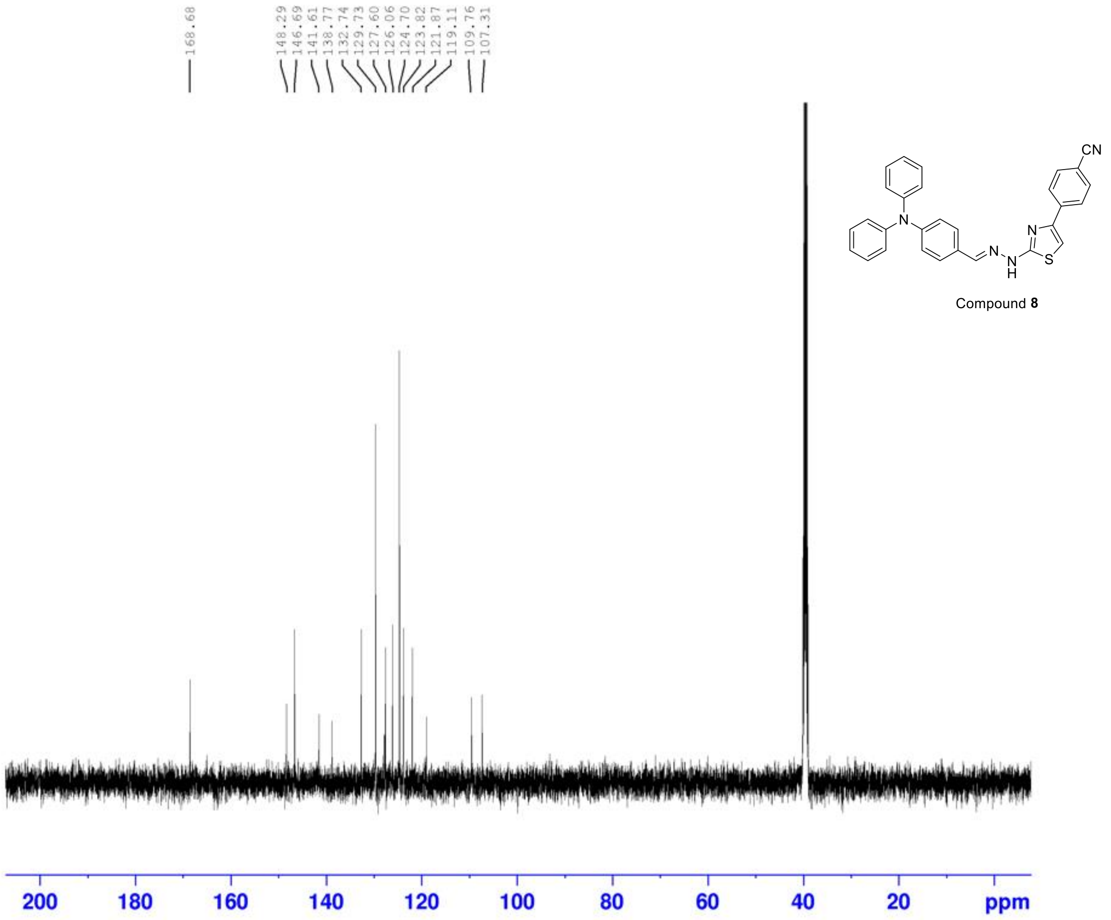

Figure S27: Mass Spectrum of compound **8**

```
Note : NBA
Inlet : Direct                          Ion Mode : FAB+
RT : 1.25 min                           Scan#: (4,8)
Elements : C 200/0, H 200/0, N 6/4, S 2/0
Mass Tolerance        : 20ppm, 10mmu if m/z < 500, 20mmu if m/z > 1000
Unsaturation (U.S.) : -0.5 - 100.0

Observed m/z Int%   Err[ppm / mmu]     U.S.   Composition
  471.1507   100.0     +4.9 /  +2.3    27.0   C 32 H 17 N 5
                       -2.3 /  -1.1    23.0   C 29 H 21 N 5 S
                       -9.4 /  -4.4    19.0   C 26 H 25 N 5 S 2
                      +17.3 /  +8.1    19.5   C 25 H 23 N 6 S 2

  472.1565    79.5     +0.6 /  +0.3    26.5   C 32 H 18 N 5
                       -6.5 /  -3.1    22.5   C 29 H 22 N 5 S
                      +20.1 /  +9.5    23.0   C 28 H 20 N 6 S
                      -13.6 /  -6.4    18.5   C 26 H 26 N 5 S 2
                      +13.0 /  +6.1    19.0   C 25 H 24 N 6 S 2
```

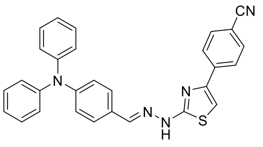

Compound **8**

```
[ Theoretical Ion Distribution ]                                   Page: 1
Molecular Formula : C29 H21 N5 S
                    (m/z   471.1518, MW   471.5852, U.S. 23.0)
Base Peak :    471.1518, Averaged MW :   471.5856(a),    471.5866(w)

    m/z        INT.
  471.1518 100.0000 ************************************************************
  472.1547  35.1959 ********************
  473.1534  10.4385 ******
  474.1536   2.1870 *
  475.1548   0.3283
  476.1562   0.0379
  477.1578   0.0035
  478.1596   0.0003
```

```
Note : NBA
Inlet : Direct                          Ion Mode : FAB+
Spectrum Type : Normal Ion [EF-Linear]
RT : 1.25 min          Scan# : (4,8)
BP : m/z 471.1507      Int.   : 7.67
Output m/z range : 432.0000 to 486.0000      Cut Level : 0.00 %
```

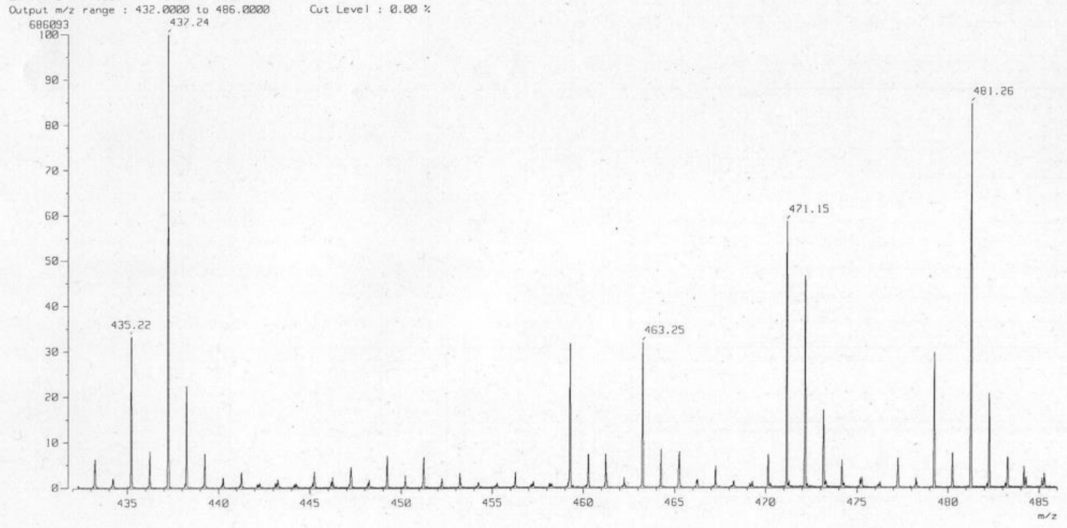

Figure S28: ^{1}H NMR Spectrum of compound **9**

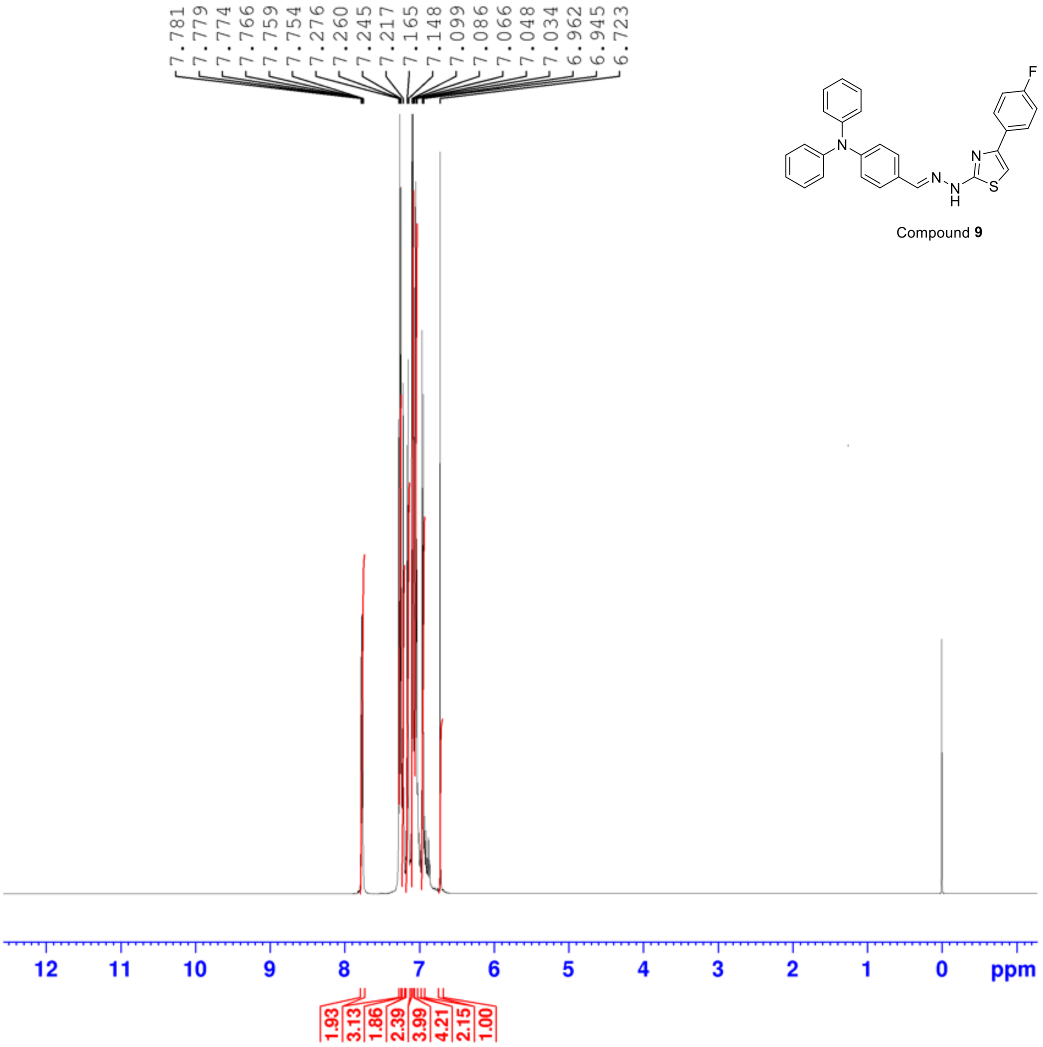

Compound **9**

Figure S29. ^{13}C NMR Spectrum of compound **9**

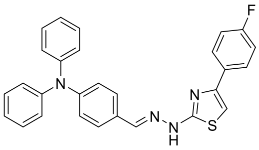

Compound **9**

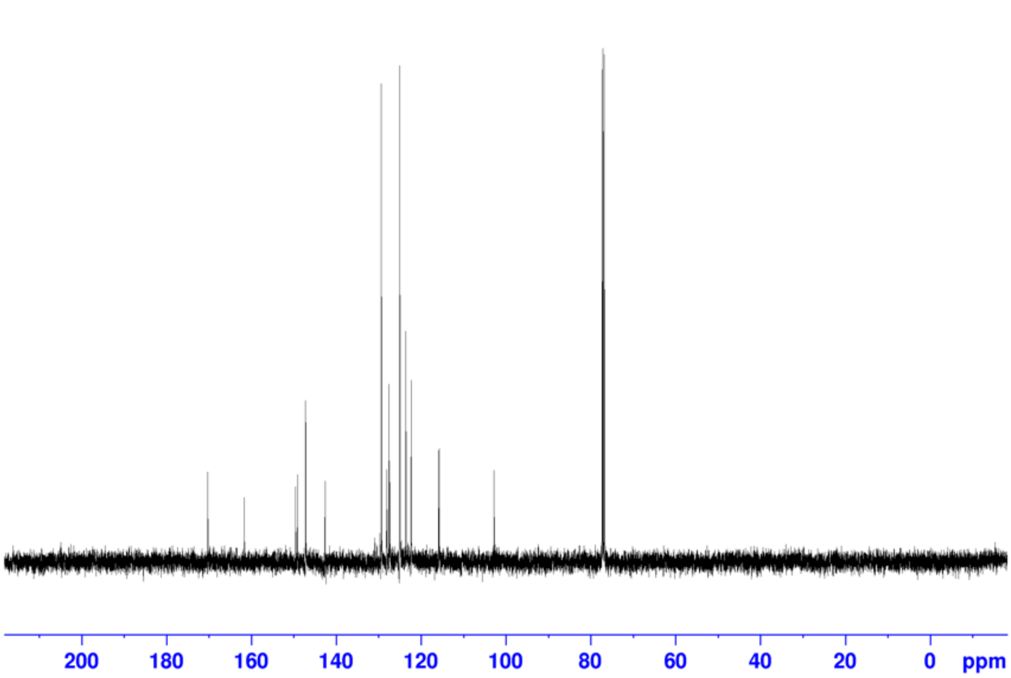

Figure S30. Mass Spectrum of compound **9**

Note : NBA
Inlet : Direct — Ion Mode : FAB+
RT : 0.88 min — Scan#: (3,6)
Elements : C 200/0, H 200/0, N 5/3, F 2/0, S 2/0
Mass Tolerance : 20ppm, 10mmu if m/z < 500, 20mmu if m/z > 1000
Unsaturation (U.S.) : -0.5 - 100.0

Observed m/z	Int%	Err[ppm / mmu]	U.S.	Composition
464.1473	99.7	-19.3 / -9.0	24.5	C 32 H 19 N 3 F
		+7.8 / +3.6	25.0	C 31 H 17 N 4 F
		+0.5 / +0.2	21.0	C 28 H 21 N 4 F S
		-6.7 / -3.1	17.0	C 25 H 25 N 4 F S 2
		+20.4 / +9.4	17.5	C 24 H 23 N 5 F S 2
465.1544	100.0	-20.8 / -9.7	24.0	C 32 H 20 N 3 F
		+6.2 / +2.9	24.5	C 31 H 18 N 4 F
		-1.0 / -0.5	20.5	C 28 H 22 N 4 F S
		-8.3 / -3.8	16.5	C 25 H 26 N 4 F S 2
		+18.8 / +8.7	17.0	C 24 H 24 N 5 F S 2

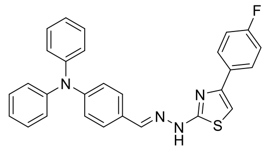

Compound **9**

[Theoretical Ion Distribution] — Page: 1
Molecular Formula : C28 H22 N4 F S
(m/z 465.1549, MW 465.5739, U.S. 20.5)
Base Peak : 465.1549, Averaged MW : 465.5742(a), 465.5753(w)

m/z	INT.	
465.1549	100.0000	**
466.1579	33.7313	********************
467.1564	9.9406	******
468.1567	2.0401	*
469.1579	0.2980	
470.1593	0.0334	
471.1609	0.0030	
472.1628	0.0002	

Note : NBA
Inlet : Direct — Ion Mode : FAB+
Spectrum Type : Normal Ion [EF-Linear]
RT : 0.88 min — Scan# : (3,6)
BP : m/z 465.1544 — Int. : 15.38
Output m/z range : 432.0000 to 486.0000 — Cut Level : 0.00 %

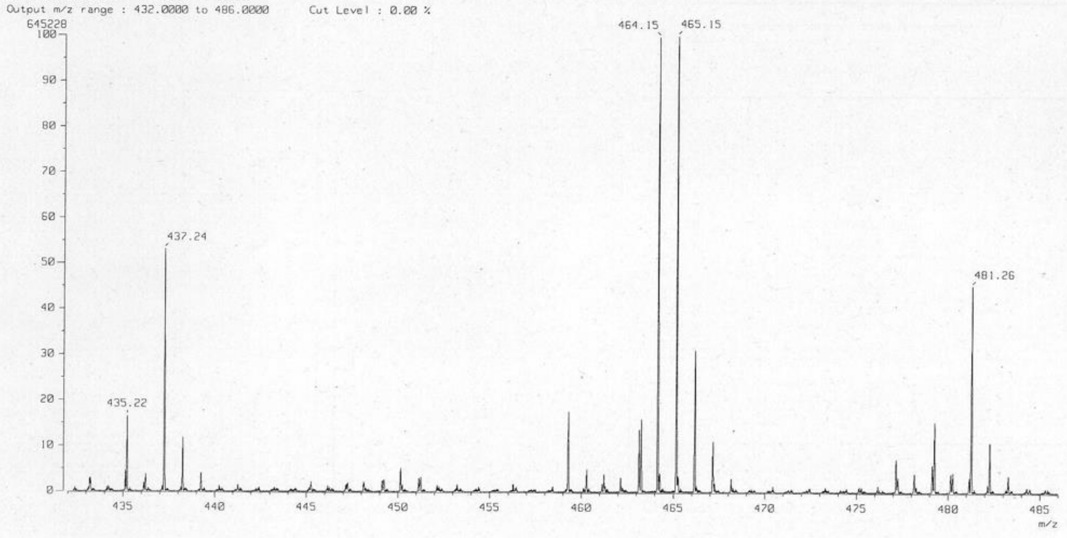

Figure S31: ^{1}H NMR Spectrum of compound **10**

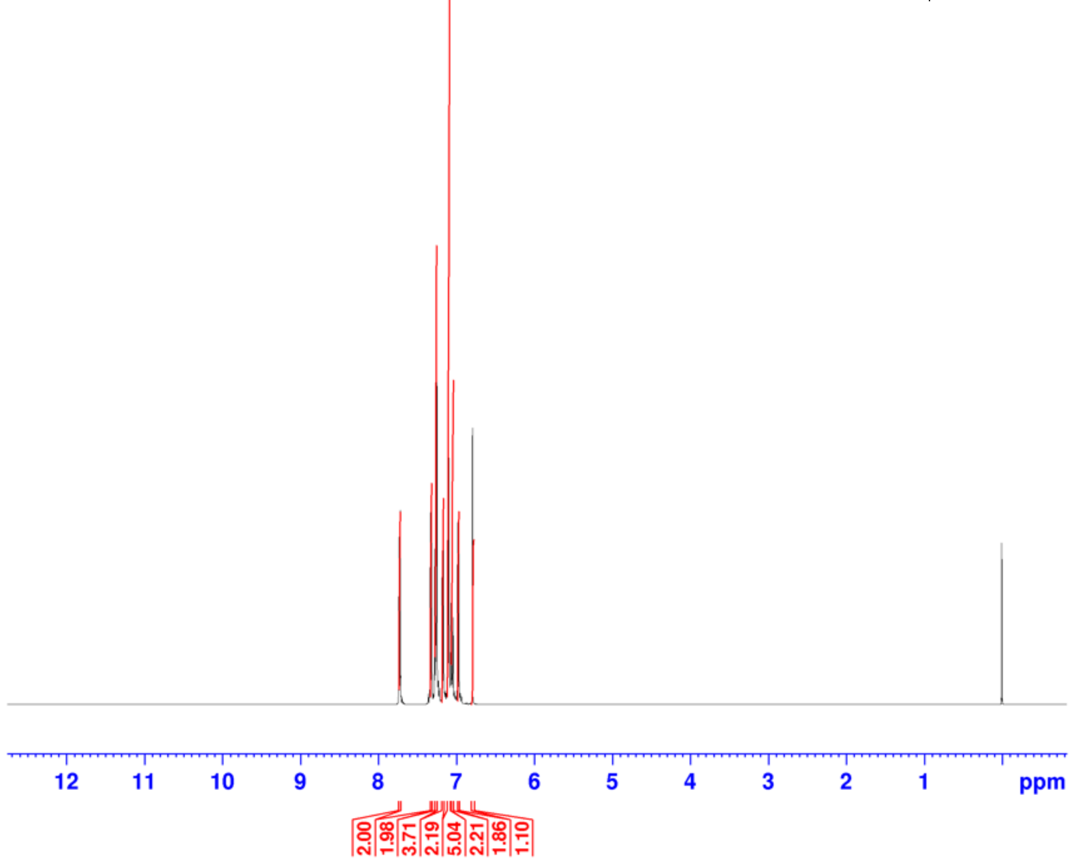

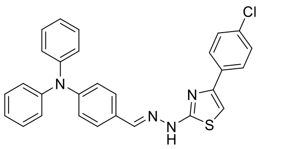

Compound **10**

Figure S32: ^{13}C NMR Spectrum of compound **10**

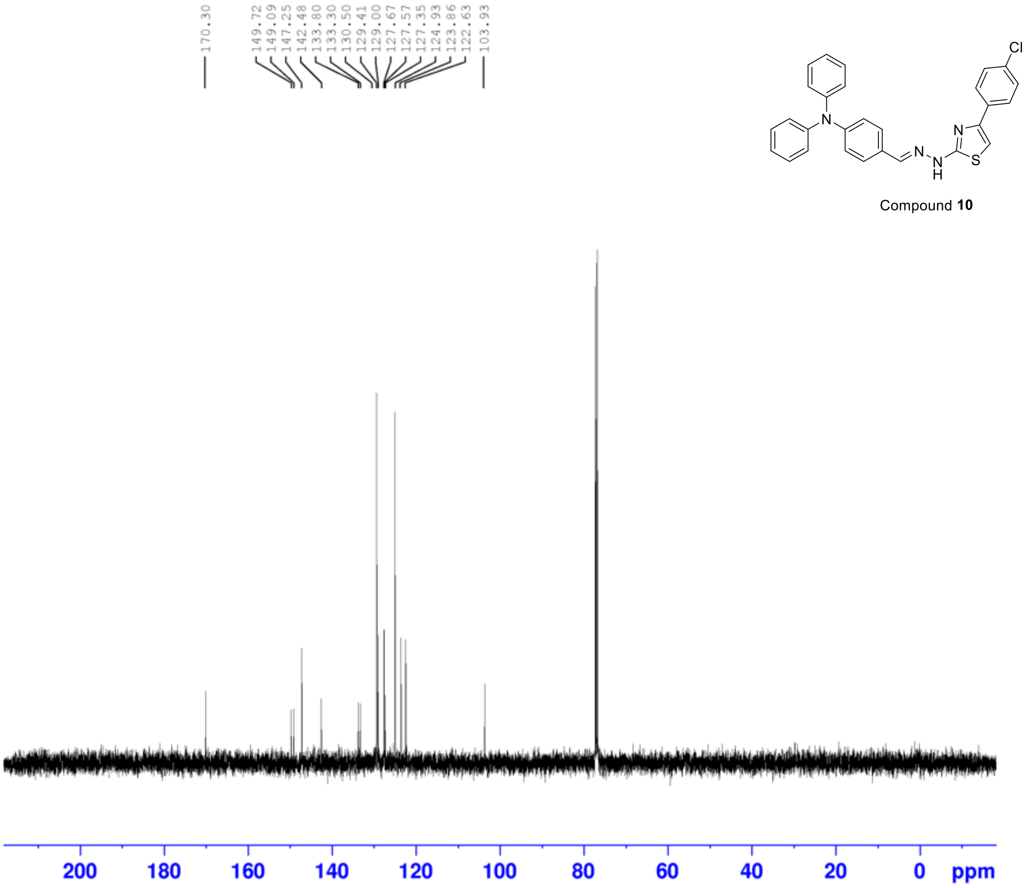

Compound **10**

Figure S33: Mass Spectrum of compound **10**

Note : NBA
Inlet : Direct   Ion Mode : FAB+
RT : 0.88 min   Scan#: (3,6)
Elements : C 200/0, H 200/0, N 5/3, Cl 2/0, S 2/0
Mass Tolerance : 20ppm, 10mmu if m/z < 500, 20mmu if m/z > 1000
Unsaturation (U.S.) : -0.5 - 100.0

Observed m/z	Int%	Err[ppm / mmu]	U.S.	Composition
481.1222	95.3	+0.5 / +0.2	24.5	C 31 H 18 N 4 Cl
		-6.5 / -3.1	20.5	C 28 H 22 N 4 Cl S
		+19.6 / +9.4	21.0	C 27 H 20 N 5 Cl S
		+15.8 / +7.6	16.0	C 26 H 25 N 3 Cl 2 S
		-13.6 / -6.5	16.5	C 25 H 26 N 4 Cl S 2
		+12.6 / +6.1	17.0	C 24 H 24 N 5 Cl S 2
		+8.8 / +4.2	12.0	C 23 H 29 N 3 Cl 2 S 2
480.1171	100.0	-16.3 / -7.8	29.5	C 33 H 14 N 5
		-20.1 / -9.7	24.5	C 32 H 19 N 3 Cl
		+6.1 / +2.9	25.0	C 31 H 17 N 4 Cl
		-0.9 / -0.5	21.0	C 28 H 21 N 4 Cl S
		-8.0 / -3.8	17.0	C 25 H 25 N 4 Cl S 2
		+18.2 / +8.8	17.5	C 24 H 23 N 5 Cl S 2
		+14.4 / +6.9	12.5	C 23 H 28 N 3 Cl 2 S 2

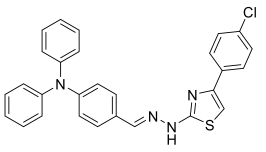

Compound **10**

[Theoretical Ion Distribution]   Page: 1
Molecular Formula : C28 H21 N4 Cl S
(m/z 480.1175, MW 481.0205, U.S. 21.0)
Base Peak : 480.1175, Averaged MW : 481.0205(a), 481.0231(w)

m/z	INT.	
480.1175	100.0000	**
481.1206	33.7163	********************
482.1156	41.9139	*************************
483.1179	12.8205	*******
484.1164	3.4749	**
485.1166	0.6853	
486.1178	0.0982	
487.1191	0.0109	
488.1207	0.0010	

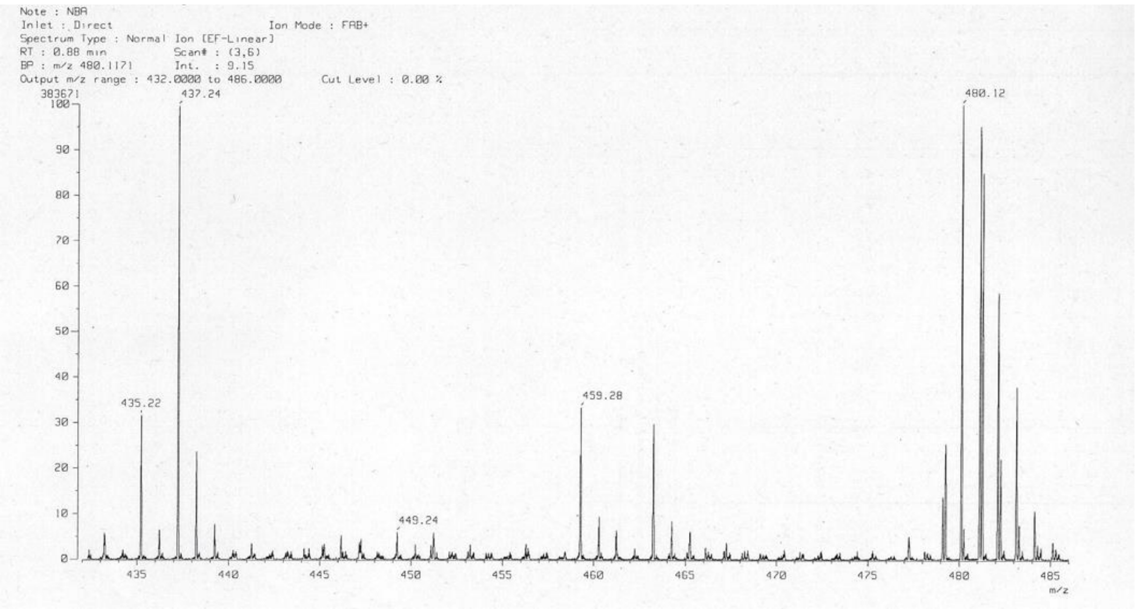

Figure S34: ^{1}H NMR Spectrum of compound **11**

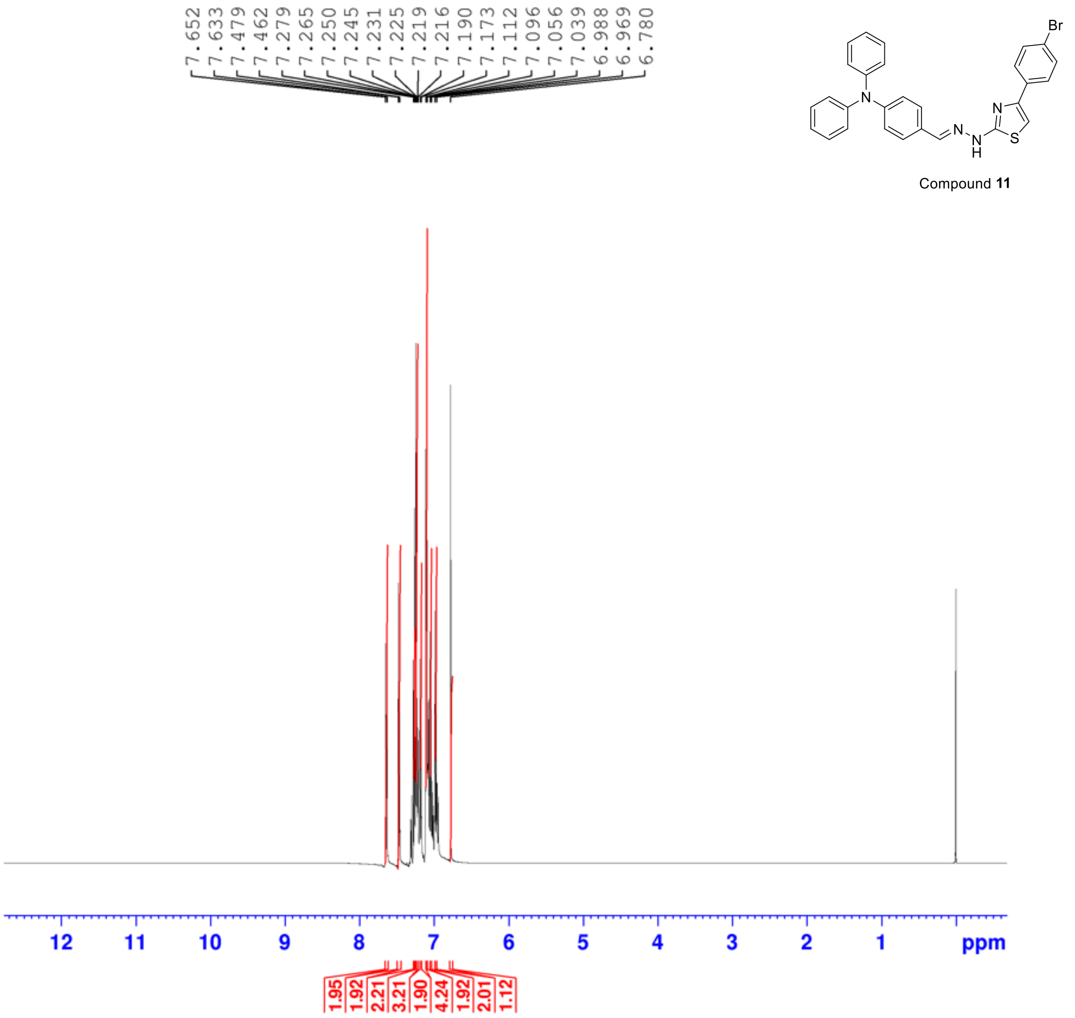

Figure S35: ^{13}C NMR Spectrum of compound **11**

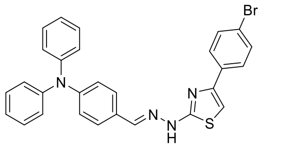

Compound **11**

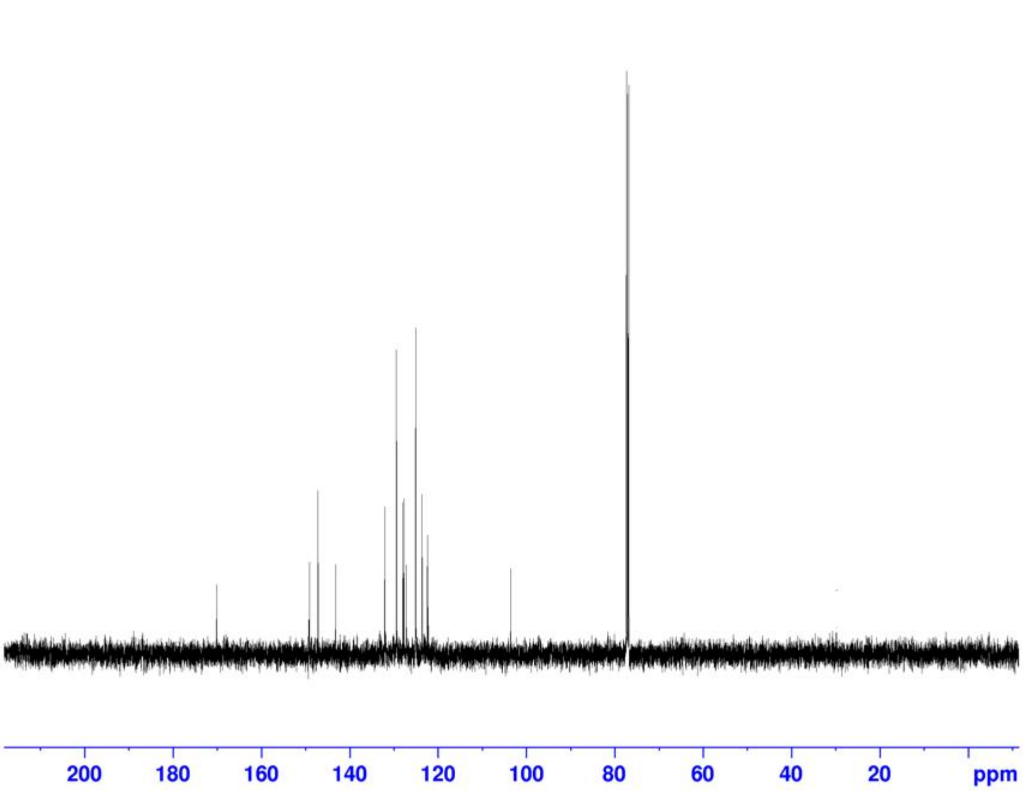

Figure S36: Mass Spectrum of compound **11**

Note : NBA
Inlet : Direct   Ion Mode : FAB+
RT : 0.88 min   Scan#: (3,6)
Elements : C 200/0, H 200/0, N 5/3, Br 2/0, S 2/0
Mass Tolerance : 20ppm, 10mmu if m/z < 500, 20mmu if m/z > 1000
Unsaturation (U.S.) : -0.5 - 100.0

Observed m/z	Int%	Err[ppm / mmu]	U.S.	Composition
524.0643	71.9	+1.3 / +0.7	25.0	C 31 H 17 N 4 Br
		-5.1 / -2.7	21.0	C 28 H 21 N 4 Br S
		+18.8 / +9.9	21.5	C 27 H 19 N 5 Br S
		-11.6 / -6.1	17.0	C 25 H 25 N 4 Br S 2
		+12.4 / +6.5	17.5	C 24 H 23 N 5 Br S 2
525.0697	81.7	-3.4 / -1.8	24.5	C 31 H 18 N 4 Br
		-9.8 / -5.2	20.5	C 28 H 22 N 4 Br S
		+14.1 / +7.4	21.0	C 27 H 20 N 5 Br S
		-16.2 / -8.5	16.5	C 25 H 26 N 4 Br S 2
		+7.7 / +4.1	17.0	C 24 H 24 N 5 Br S 2

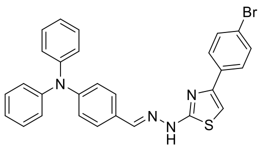

Compound **11**

[Theoretical Ion Distribution]   Page: 1
Molecular Formula : C28 H22 N4 Br S
(m/z 525.0749, MW 526.4795, U.S. 20.5)
Base Peak : 527.0731, Averaged MW : 526.4793(a), 526.4821(w)

m/z	INT.	
525.0749	93.2678	**
526.0779	31.4604	******************
527.0731	100.0000	**
528.0759	32.5067	*******************
529.0744	9.2969	*****
530.0747	1.8822	*
531.0758	0.2732	
532.0772	0.0305	
533.0788	0.0027	
534.0807	0.0002	

Note : NBA
Inlet : Direct   Ion Mode : FAB+
Spectrum Type : Normal Ion [EF-Linear]
RT : 0.88 min   Scan# : (3,6)
BP : m/z 526.0651   Int. : 3.38
Output m/z range : 476.0000 to 530.0000   Cut Level : 0.00 %

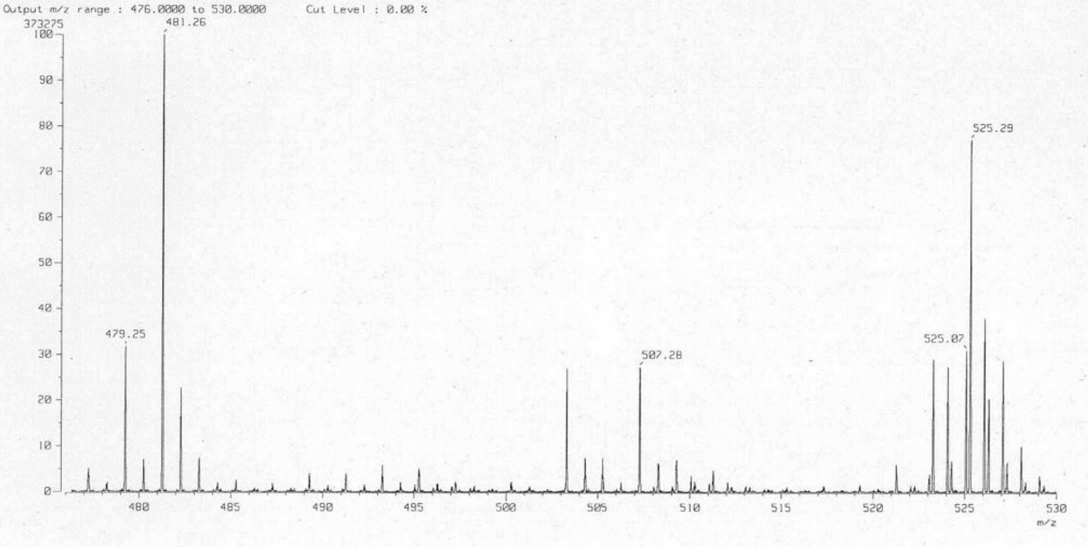

Figure S37: ^{1}H NMR Spectrum of compound **12**

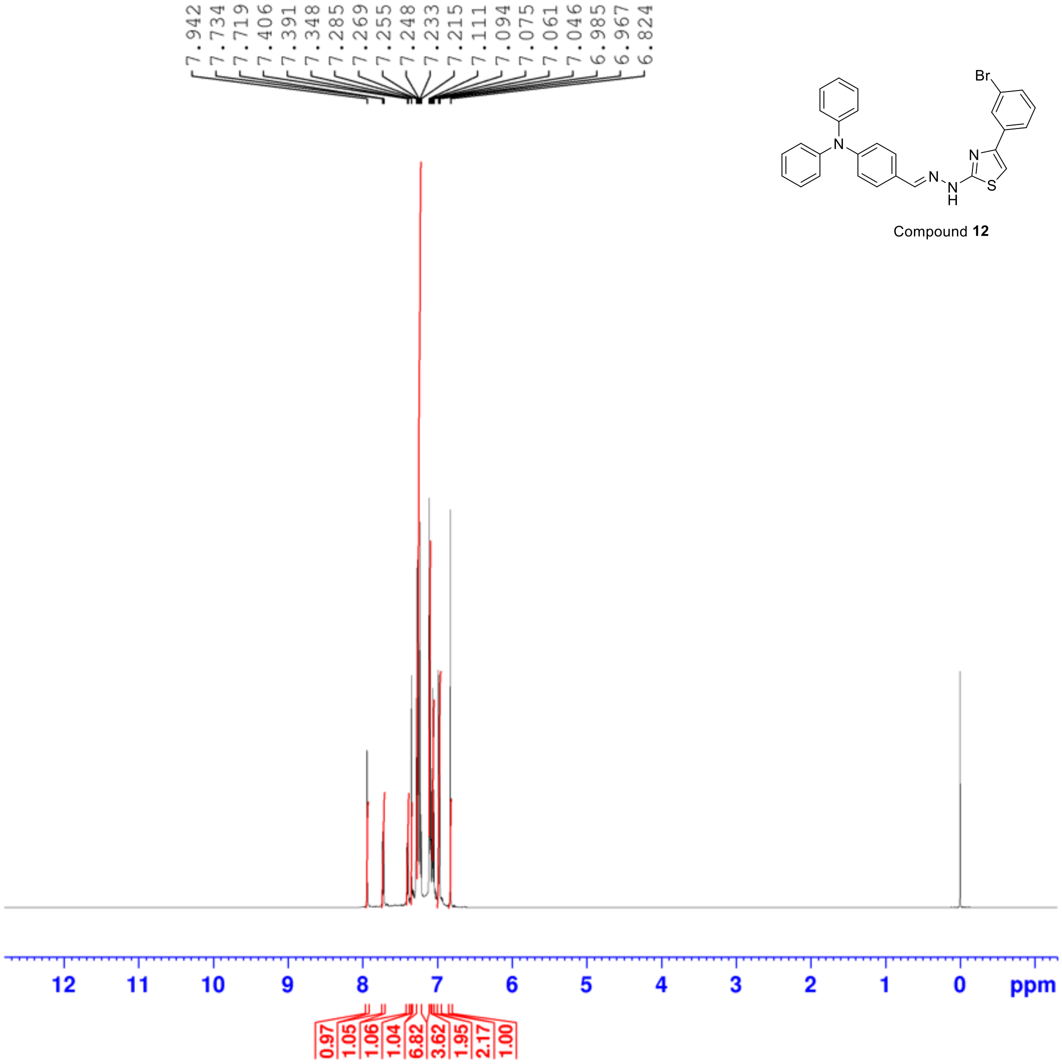

Figure S38: ^{13}C NMR Spectrum of compound **12**

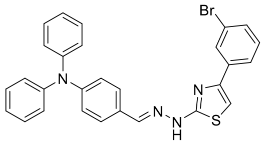

Compound **12**

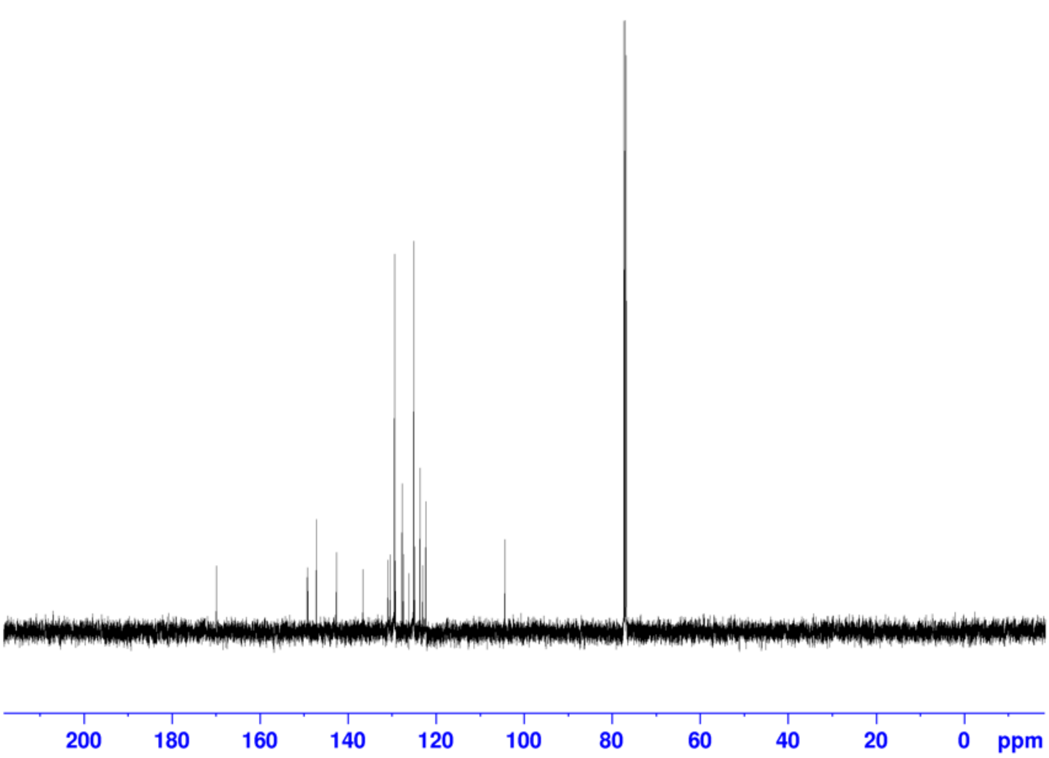

Figure S39: Mass Spectrum of compound **12**

```
Note : NBA
Inlet : Direct                                  Ion Mode : FAB+
RT : 0.75 min                                   Scan#: (2,6)
Elements : C 200/0, H 200/0, N 5/3, Br 2/0, S 2/0
Mass Tolerance       : 20ppm, 10mmu if m/z < 500, 20mmu if m/z > 1000
Unsaturation (U.S.) : -0.5 - 100.0

Observed m/z Int%    Err[ppm / mmu]     U.S.  Composition
  524.0653    82.2     +3.1 /  +1.6     25.0  C 31 H 17 N 4 Br
                       -3.3 /  -1.7     21.0  C 28 H 21 N 4 Br S
                       -9.7 /  -5.1     17.0  C 25 H 25 N 4 Br S 2
                      +14.3 /  +7.5     17.5  C 24 H 23 N 5 Br S 2

  525.0704    83.1     -2.1 /  -1.1     24.5  C 31 H 18 N 4 Br
                       -8.5 /  -4.5     20.5  C 28 H 22 N 4 Br S
                      +15.4 /  +8.1     21.0  C 27 H 20 N 5 Br S
                      -15.0 /  -7.9     16.5  C 25 H 26 N 4 Br S 2
                       +9.0 /  +4.7     17.0  C 24 H 24 N 5 Br S 2
```

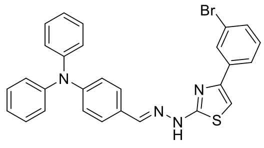

Compound **12**

```
[ Theoretical Ion Distribution ]                                    Page: 1
Molecular Formula : C28 H22 N4 Br S
                           (m/z   525.0749, MW   526.4795, U.S. 20.5)
Base Peak :    527.0731, Averaged MW :    526.4793(a),    526.4821(w)

     m/z          INT.
  525.0749   93.2678 ******************************************************
  526.0779   31.4604 ******************
  527.0731  100.0000 **********************************************************
  528.0759   32.5067 *******************
  529.0744    9.2969 *****
  530.0747    1.8822 *
  531.0758    0.2732
  532.0772    0.0305
  533.0788    0.0027
  534.0807    0.0002
```

```
Note : NBA
Inlet : Direct                              Ion Mode : FAB+
Spectrum Type : Normal Ion [EF-Linear]
RT : 0.75 min                Scan# : (2,6)
BP : m/z 479.2472            Int.   : 2.62
Output m/z range : 476.0000 to 530.0000       Cut Level : 0.00 %
```

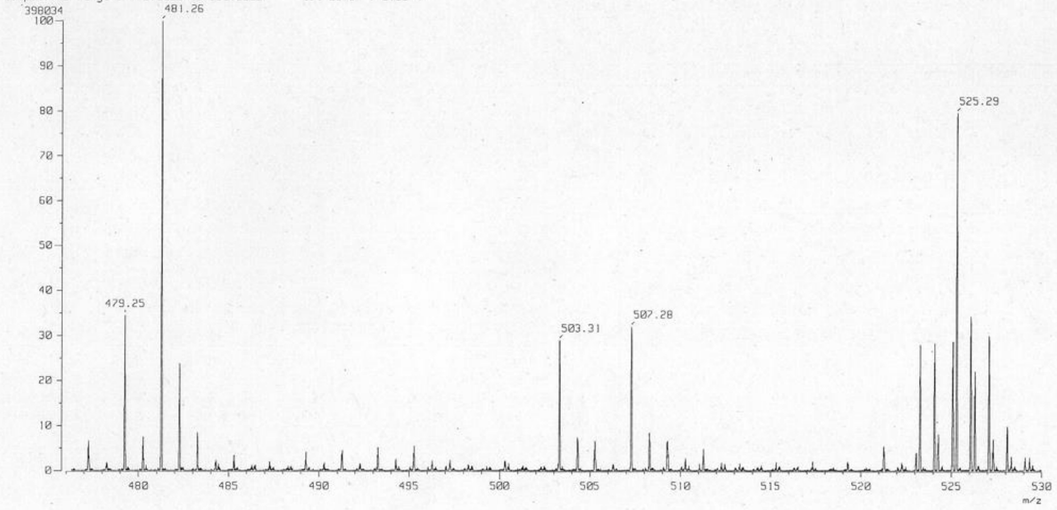

Supplement: Supplementary file 1 [file pharmaceuticals-19-00416-s001.zip › pharmaceuticals-4187590-supplementary.pdf]
